# Highly Efficient, Tripodal Ion-Pair Receptors for Switching Selectivity between Acetates and Sulfates Using Solid–Liquid and Liquid–Liquid Extractions

**DOI:** 10.3390/ijms21249465

**Published:** 2020-12-12

**Authors:** Marta Zaleskaya, Łukasz Dobrzycki, Jan Romański

**Affiliations:** Faculty of Chemistry, University of Warsaw, Pasteura 1, 02-093 Warsaw, Poland; mzaleskaya@chem.uw.edu.pl (M.Z.); dobrzyc@chem.uw.edu.pl (Ł.D.)

**Keywords:** anion recognition, cation recognition, ion pair receptors, host-guest interactions, squaramide, crown ether

## Abstract

A tripodal, squaramide-based ion-pair receptor **1** was synthesized in a modular fashion, and ^1^H NMR and UV-vis studies revealed its ability to interact more efficiently with anions with the assistance of cations. The reference tripodal anion receptor **2**, lacking a crown ether unit, was found to lose the enhancement in anion binding induced by presence of cations. Besides the ability to bind anions in enhanced manner by the “single armed” ion-pair receptor **3**, the lack of multiple and prearranged binding sites resulted in its much lower affinity towards anions than in the case of tripodal receptors. Unlike with receptors **2** or **3**, the high affinity of **1** towards salts opens up the possibility of extracting extremely hydrophilic sulfate anions from aqueous to organic phase. The disparity in receptor **1** binding modes towards monovalent anions and divalent sulfates assures its selectivity towards sulfates over other lipophilic salts upon liquid–liquid extraction (LLE) and enables the Hofmeister bias to be overcome. By changing the extraction conditions from LLE to SLE (solid–liquid extraction), a switch of selectivity from sulfates to acetates was achieved. X-ray measurements support the ability of anion binding by cooperation of the arms of receptor **1** together with simultaneous binding of cations.

## 1. Introduction

Molecular receptors that respond selectively to the presence of desired ionic species are a subject of intense study because of the fundamental role that ions play in many fields, such as monitoring environmental pollution, industrial, and biological processes, the regulation of ion transport in organisms and medical diagnostic methods [1,2,3,4]. However, due to the variety of ions and the environment in which they are found, their recognition by receptors requires an individual approach. The main limitation on the use of monotopic receptors capable of interacting with one type of ion, e.g., with cations or anions, is that a suitable lipophilic counterion is required for effective ion recognition. Otherwise, the so-called counterion effect occurs due to the high-energy costs of separating a single anion/cation or solvation sphere [5,6,7,8,9,10].

A solution to this problem may lie in the utilization of heteroditopic ion-pair receptors able to bind cations and anions simultaneously [5,11,12,13]. The geometric structure of the receptor also has a great impact on tuning the binding strength and its selectivity [14,15,16]. More complex receptive architectures, such as multimacrocyclic systems, offer extra stability and enhancement of guest binding. Despite their high efficiency, however, multimacrocyclic receptors pose problems in low-yield synthesis, which requires the use of high-dilution technique [5,17]. On the other hand, more accessible and tunable acyclic receptors are usually less selective than multimacrocyclic ones.

A sensible balance may be struck by tripodal receptors, which fall between cyclic and acyclic ligands in terms of their preorganization. This means that they are able to interact with ions more forcibly than analogous acyclic ones [18,19,20,21]. The tripodal molecular platform design ensures three arms to which ligating groups can be attached [22,23,24]. The selectivity of a tripodal receptor also depends on the directional orientation of binding groups, flexibility of its arms, and its cavity size. Besides the most frequently employed, flexible tris (2-aminoethyl) amine (TREN) platform [16,20], the 1,3,5-trisubstituted benzene rigid molecular scaffold is also well known and versatility used in the synthesis of tripodal receptors. The tripodal conjugation based on trisubstituted benzene is an attractive approach to the development of powerful ion-pair receptors in view of the alternate arrangement of the ethyl and aminomethylene groups. Such a template provides a rigid scaffold where all the three amino groups are on the same side of the aromatic ring [25,26,27]. Trisubstituted benzene based receptors mainly include ligands for anions; however, there have been a few reports on cation recognition [28,29,30]. Among the anion receptors, efficient complexation has been achieved by moieties such as amide [31], urea [32,33], thiourea [34], pyrrole [35,36,37], squaramide [23,38,39], and C-H^…^X^−^ hydrogen bonds [22,40,41]. At the same time, much attention has been focused on the sensing of explosives and the hydrolysis products of warfare agents (e.g., the nerve gas sarin), which are also strong environmental pollutants. Fluorescent tripodal anion sensors based on a 1,3,5-triethylbenzene scaffold display an aggregation-induced emission enhancement response to phosphonate and phosphate anions and can be used as optical sensors [42,43]. Tripod conjugates have also been shown to bind tricarboxylates [25], including citrate [44] and other carboxylates [45] with high association constants, even in an aqueous environment. As 1,3,5-trisubstituted benzene based receptors feature a well-dispersed but convergent three-dimensional (3D) array of functionalities, tripodal conjugation is an attractive approach for the development of effective anion transporters [26,34]. The trialkylbenzene core can also provide some degree of preorganization into a conical conformation, where all three anion binding arms point in one direction, such as dimeric capsular aggregates [46,47,48].

While myriads of tripodal anion receptors have been reported, to the best of our knowledge there is only one report on an ion-pair receptor built on TREN platform and utilizing amide and crown ether moieties to binds anions and cations, respectively [49]. On the other hand, currently there is increased interest in synthetic receptors containing a squaramide based anion binding domain [50,51,52,53]. Numerous studies by Costa have shown the versatility of squaramides, which have been utilized in the formation of self-assembly structures [54,55], hydrogel formation [56], binding and sensing an anions [38], peptidomimetic synthesis [57,58], and potential treatment of Chagas disease [59]. These compounds are able to form strong hydrogen bonds with anions, showing a greater binding affinity than the analogous urea or thiourea based receptors. Fabbrizzi’s group made a direct comparison of analogous compounds possessing urea derivatives and squaramide and found that diarylsquaramides are extremely effective receptors capable of interacting with anions in highly polar solvents [60]. Due to their low cytotoxicity and stable structures, squaramides provide a convenient recognition site for detecting and transporting anions across biological membranes [61,62]. Continual research by Gale and others has shown that squaramides are effective chloride transporters and can serve as potent drugs in the treatment of channellopathies [63,64,65,66]. Furthermore, work with Sessler shows that these types of transporters are responsible for cellular autophagy disruption and cell apoptosis, which could potentially be exploited for therapeutic use against cancer [67]. Squaramide functionalized amino acids were studied by the Jolliffe group, leading to effective and selective compounds capable of binding extremely hydrophilic anions such as sulfates [68,69].

**Figure 1:**
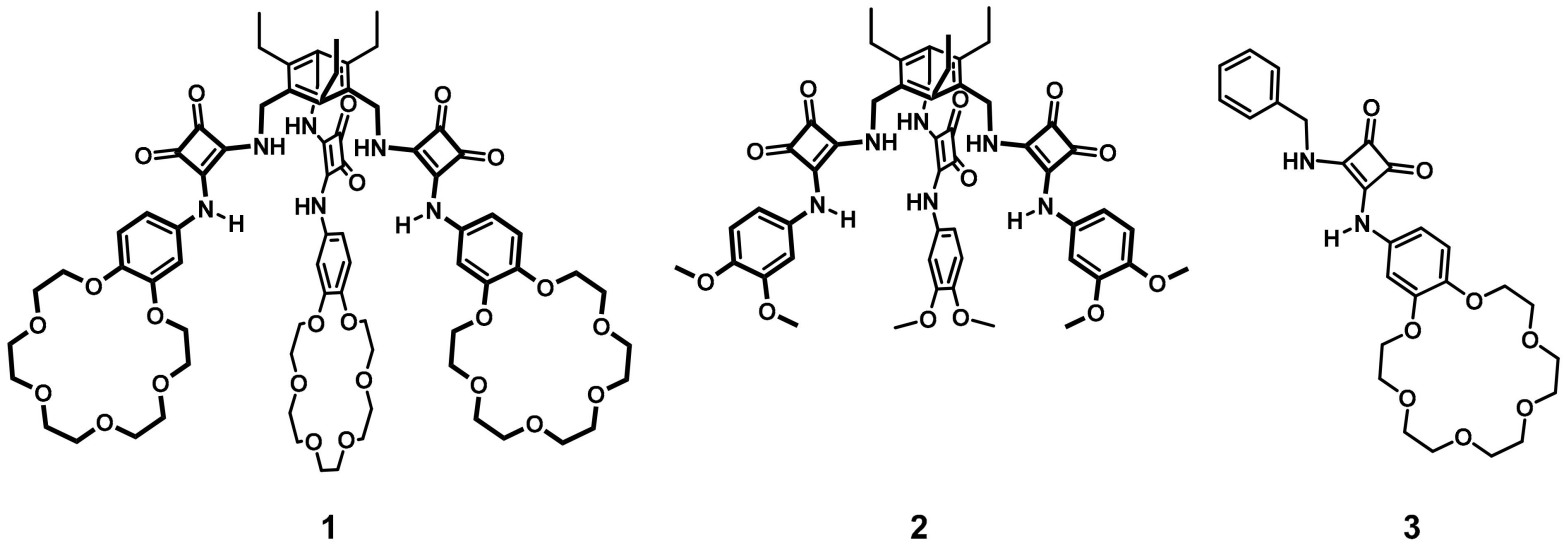
Structures of studied receptors **1**–**3**.

Compared to anion receptors, however, there are very few reports on squaramide based ion-pair receptors [70,71,72]. Our own contribution to this field reported evidence of higher affinity of squaramide based ion-pair receptors over their homotopic analogues (anion receptors), which led to ion-pair receptors and sensors able to recognize ionic species in highly competitive, aqueous media or even to extract or transport extremely hydrated anions in the form of alkali metal salts [53,73,74,75]. In the present paper, therefore, we proposed to combine all the superlatives, which came from three features: a tripodal platform, the binding abilities of squaramides and ion-pair receptors. We present a tripodal ion-pair receptor **1** built on a platform of 1,3,5-tris(aminomethyl)-2,4,6-triethylbenzene, which can form arrays of six hydrogen-bond donors by means of properly oriented squaramide units. Its action as ion-pair receptor was assured by the installation of cation binding site in close proximity to the anion binding site, which should result in anion binding reinforcement. In order to verify the design principle of receptor **1**, two reference receptors **2** and **3** were proposed. Receptor **2** was selected as a homotopic anion receptor lacking a cation binding site, while heteroditopic ion-pair receptor **3** was chosen to mimic a single arm of receptor **1** (Figure 1).

## 2. Results and Discussion

### 2.1. Receptor Design and Synthesis

**Scheme 1:**
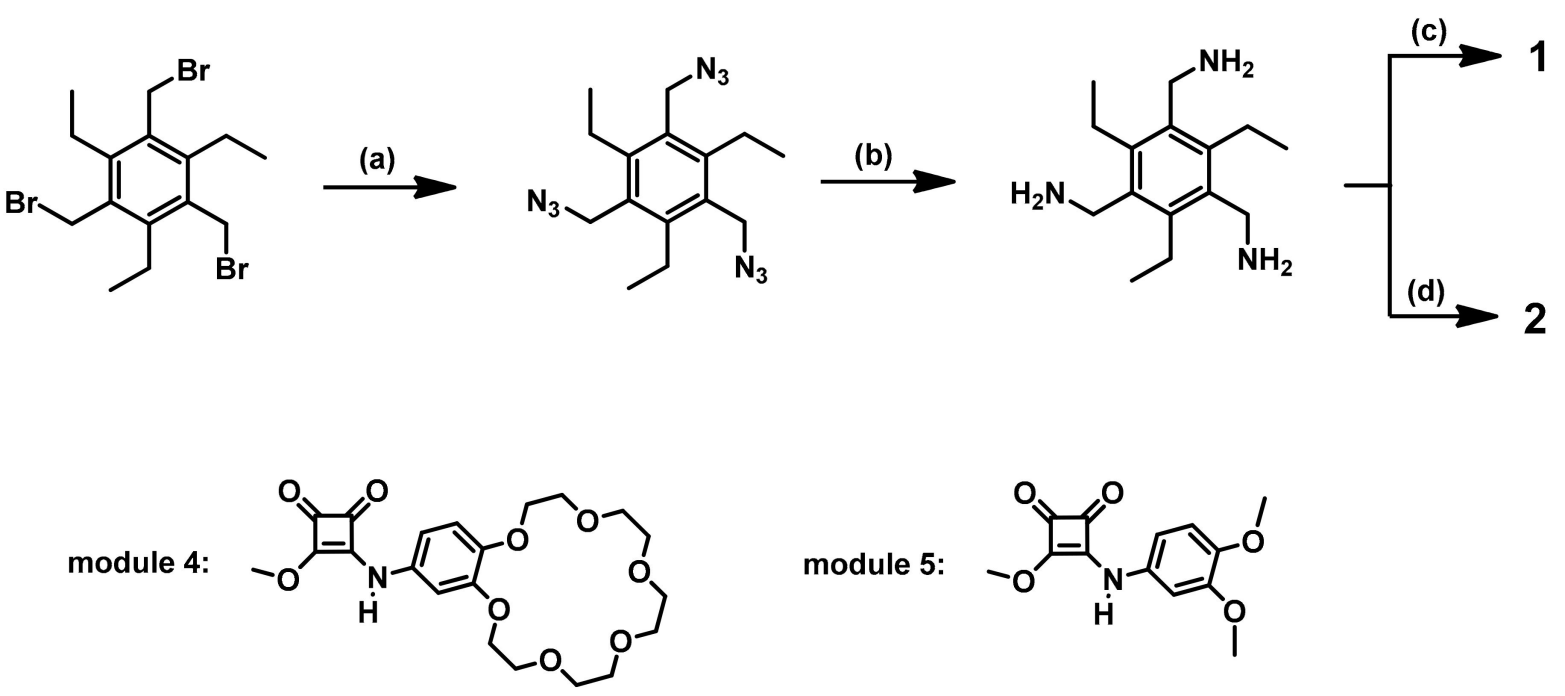
Reagent and conditions: (**a**) NaN_3_, DMF/DCM, H_2_O, overnight, 80 °C, 96%; (**b**) H_2_, Pd/C, THF/MeOH, overnight, r.t., quantitative; (**c**) 3 equiv. of module 4, MeOH, Et_3_N, overnight, r.t., 52%; (**d**) 3 equiv. of module 5, MeOH, Et_3_N, overnight, r.t., 60%.

We synthesized the ion-pair receptor **1**, displaying a three-dimensional molecular architecture, in a modular approach (Scheme 1). 1,3,5-tris(aminomethyl)-2,4,6-triethylbenzene is the key structural element that allows for the construction of receptor and was obtained by the two-step procedure described in the literature with small modifications [76,77]. Specifically, the nucleophilic displacement of commercially available 1,3,5-tris(bromomethyl)-2,4,6-triethylbenzene using sodium azide led to the formation of corresponding triazide derivative in 96% yield. The transformation of triazide into 1,3,5-tris(aminomethyl)-2,4,6-triethylbenzene was achieved by means of reduction, using hydrogen and heterogeneous catalyst Pd/C. Exploiting the ability of dimethyl squarate to undergo sequential amidation reactions, 4-aminobenzo-18-crown-6 ether and dimethyl squarate were firstly coupled, to afford monoester module **4.** The synthesis of the target receptor was accomplished by further reaction of three equivalents of module **4** with one equivalent of 1,3,5-tris(aminomethyl)-2,4,6-triethylbenzene in the presence of triethylamine. The reference receptor **2** without the crown ether moiety was synthesized according to the method described for the receptor **1** applying reaction of module **5** with 1,3,5-tris(aminomethyl)-2,4,6-triethylbenzene. Module **5** was used as a counterpart of **4** and possesses two electron donating alkoxylate substituents instead of crown ether unit. Ion-pair receptor **3** was designed as an analogue of the single arm of receptor **1** and was synthesized by amidation of monoester module **4** with benzylamine.

### 2.2. Binding Studies

**Figure 2:**
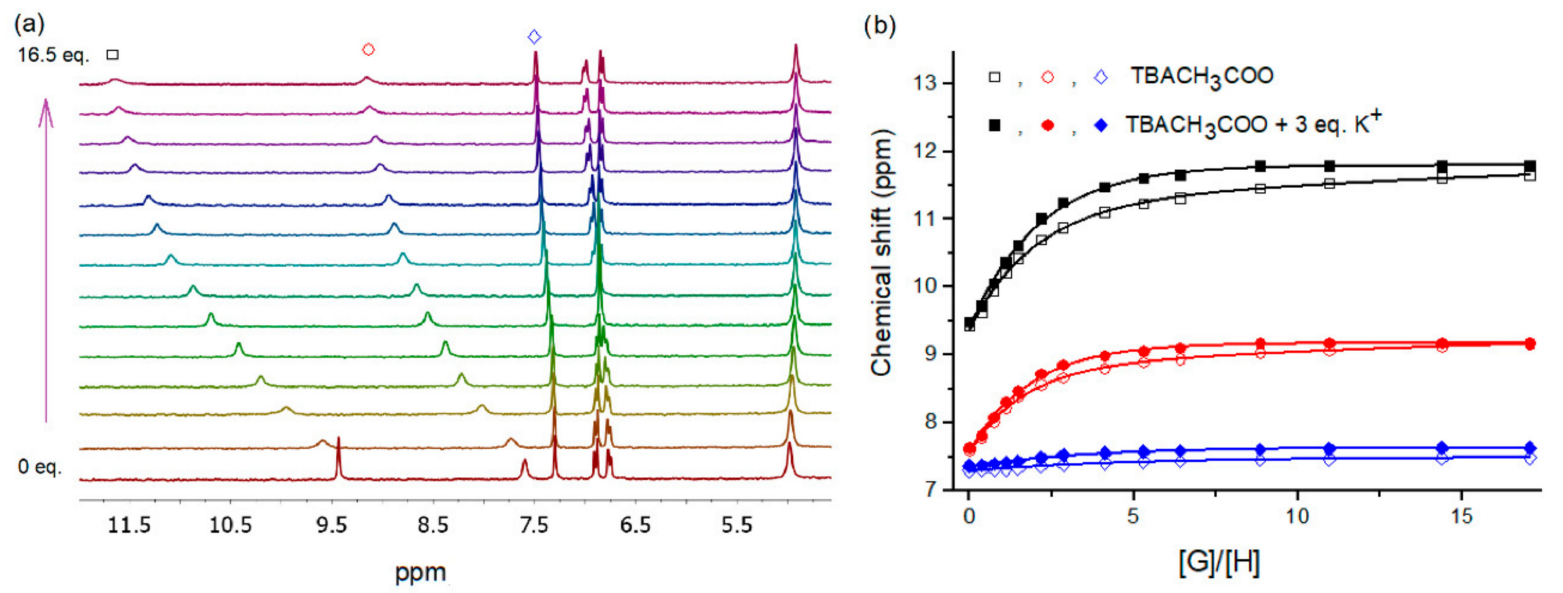
Representative stack plots of partial ^1^H NMR spectra of **1** upon addition of TBACH_3_COO in DMSO-*d_6_* (**a**) and selected binding isotherms of receptor **1** (**b**) for TBACH_3_COO alone and with the presence 3 equiv. KPF_6_.

The binding properties of receptor **1** towards selected anions in solution were studied via ^1^H NMR spectroscopy using DMSO-*d*_6_ as the solvent. The ^1^H NMR titration experiments were performed by adding anions (as their tetrabutylammonium salts) to the receptor solution, including Cl^−^, Br^−^, NO_3_^−^, NO_2_^−^, SO_4_^2−^, H_2_PO_4_^−^, CH_3_COO^−^, PhCOO^−^. Fitting of the resulting data to the 1:1 binding isotherm gave the apparent stability constants (Ka) reported in Table 1 (Supplementary Materials, Figures S10–S30). Exposure of receptor **1** to Cl^−^, Br^−^, NO_2_^−^ anions, as a result of the formation of hydrogen bonds with the anions, caused a downfield shift of both the squaramide NH proton signals (Δδ up to 0.89 ppm); small perturbations of aromatic CH signals were also observed. The least pronounced changes in resonances were observed for the signal attributed to the methylene protons directly connected with the squaramide unit. Signals corresponding to the ethyl group were unchanged upon titration, which confirms that, as we had assumed, the three ethyl substituents are located at the opposite side of the phenyl plane to the arms bearing binding domains and do not interact with complexed anions. Addition of CH_3_COO^−^ and PhCOO^−^ anions to receptor **1** caused more distinct downfield shifts for squaramide protons (Δ*δ* up to 2.18 ppm); one aromatic proton moved upfield while the other two aromatic protons shifted downfield (Figure 2). Analyses of these data resulted in the highest stability constants for complexes of **1** with carboxylate anions among all the anions tested. On the other hand, the addition of NO_3_^−^ anions to receptor **1** solution in DMSO-*d_6_* resulted in very low changes in the resonances of NH signals and others, which precludes the accurate determination of a stability constant for this complex in such competitive media. Interestingly, we could not fit the data collected from the titration of receptor **1** with the dihydrogen phosphate or sulfate anions to an appropriate model and found that upon additions of these anions the signals corresponding to the NH protons, as well as aromatic ones, were changed inconsistently. In such cases, we found that the signal corresponding to the methylene protons neighboring squaramide unit was also shifted, which suggests that this group is engaged in the binding event, which was not the case for the other salts tested. Upon a gradual addition of sulfate anions, the signals corresponding to both NH protons apart from a downfield shift were initially broadened, and after approximately 2.5 equivalents of anions was exceeded, they were sharpened. The binding isotherms obtained by tracking the squaramide as well as aromatic protons showed a two-step profile and we were unable to fit the obtained data to an appropriate model and calculate a stability constant. Interestingly, at the early stage of addition of sulfate anions to the receptor **1** solution, an additional signal in the range of 9.61–9.67 ppm appears. The same behavior was noted for the titration of **2** with sulfates. We concluded that such a signal came from a molecule of water, which is involved in the binding event. To verify this assumption, we conducted additional analyses. ROESY confirmed the exchange coupling between the water molecule (Δ *δ* = 3.31 ppm) and the investigated signal. HSQC showed no correlation between this proton with any carbon. Furthermore, DOSY experiments demonstrated that the diffusion coefficient of this species (D = 0.96 × 10^−9^ m^2^ s^−1^) is very close to the diffusion coefficient of water (D = 1.04 × 10^−9^ m^2^ s^−1^) and almost one magnitude of order higher than the diffusion coefficient of the receptor (D = 0.14 × 10^−9^ m^2^ s^−1^), (Supplementary Materials, Figures S31, S32, S62–S64).

While conducting the ^1^H NMR experiments, slow exchange processes were observed during the titration of **1** with dihydrogen phosphate anions. This observation can be rationalized by a three-step binding mechanism involving the change of the binding mode. The initial step was finished after approximately 0.4 equivalent of dihydrogen phosphate anions was added, at which point a new set of signals corresponding to amide, aromatic, and methylene protons appeared, with the continuous vanishing of the initial signals up to the addition of approximately 1 equivalent of anions. Specifically, both sharp signals of squaramide protons, which initially resonated at 7.60 and 9.44 ppm were successively changed into broad signals at 8.80 and 10.70 ppm, respectively (Supplementary Materials, Figures S33 and S34). Similarly, the signals corresponding to aromatic and methylene protons at 7.30 and 4.98 moved to 7.35 and 4.90 ppm, respectively. Exceeding the addition of one equivalent of hydrogen phosphate caused further, consistent changes without evidence of a slow exchange process, which may arise from rapid equilibration of multiple binding modes.

To check whether the presence of the crown ether unit in the receptor **1** structure encourages simultaneous binding of ion pairs and may enhance anion binding upon assistance of cation, we carried out analogous titration experiments in the presence of 3 equivalents K^+^ or Na^+^ (added as potassium hexafluorophosphate or sodium perchlorate). Indeed, the apparent stability constant for in situ generated sodium or potassium chloride complexes with receptor **1** were fairly higher than for complex of **1** with chloride anions, indicating moderate cooperation in ion pair binding. As expected for benzo-18-crown-6 ether based receptors, the higher enhancement was observed for potassium rather than sodium chloride association. For this reason, an extension of the ion pair binding study was carried out for complexes of **1** with in situ generated potassium salts. Inspection of Table 1 shows that the enhancement factor, defined as K_KX_/K_TBAX_, reached the highest value of 2.5 for bromide recognition and the least pronounced enhancement was observed for carboxylates. When cation was added (as KPF6 salt) to the solution of **1** in DMSO-*d_6_* the signals corresponding to the aromatic and crown ether protons were perturbed and the signals corresponding to squaramide protons were slightly shifted downfield from 7.59 and 9.43 ppm to 7.63 and 9.50 ppm, respectively. This confirms that cation complexation induces electron density charge of the nearby anion binding site on the phenyl ring, triggering an increase in anion binding [53]. Then, we examined reference receptor **2**, lacking a crown ether unit, and found that it was able to recognize chloride anions at comparable strength to receptor **1** with an apparent association constant of K_TBACl_ = 292 M^−1^. However, the absence of a cation binding domain in the structure of receptor **2** caused a serious limitation in the binding of ion pairs. Specifically, the addition of three equivalents of KPF_6_ to the solution of **2** in DMSO-*d_6_* did not cause any perturbations in the signals corresponding to squaramide protons in ^1^H NMR spectrum, and the data collected from titration with chloride anions upon assistance of potassium cations clearly indicate the disability of receptor **2** to bind anions in enhanced manner. On the other hand, the single-armed receptor **3** was found to bind chloride anion approximately 2.3 times less tightly than **1** or **2.** This comparison clearly demonstrates the cooperative action of three anion binding sites of tripodal receptors. Although ion-pair receptor **3** is prone to recognize anions in an enhanced manner, in the presence of cations it is still less effective in potassium chloride recognition even than tripodal anion receptor **2**. This supports the idea that two cooperative effects are harnessed in the action of receptor **1**, namely cooperative binding by multiple and properly reorganized binding sites and enhancement in anion binding by co-bounded cations.

A further technique used to analyze the interactions of receptors with ion pairs and to support the aforementioned findings was UV-vis spectroscopy. Because of the low value of the stability constants obtained during titration of receptors in DMSO, the binding studies were performed in less competitive media. Besides the expected higher values of Ka’s suitable for UV-vis measurements, the changing of the solvent to acetonitrile was also aimed at verifying whether receptor **1** is able to recognize nitrate ions, which was not affordable in DMSO. First, we carried out titrations of receptors **1**–**3** with chloride anions (TBACl) and in situ generated potassium and sodium chlorides (mixtures of TBACl and NaClO_4_ or KPF_6_). Upon addition of incremental equivalents of salts into the receptor solutions, a bathochromic shift in the absorption band was observed, which allowed the stability constant to be determined (Table 2, Supplementary Materials, Figures S45–S61). We found that receptor **1** is able to bind chloride anions with stability constants of K_TBACl_ = 3.68 × 10^4^ M^−1^ while anion receptor **2** recognized this anion more effectively with K_TBACl_ = 4.09 × 10^4^ M^−1^. In fact, both of the receptors possess electron donating substituents (-O-alkyl) on the phenyl rings directly linked with the squaramide, so the higher ability to recognize anions by receptor **2** can mostly be rationalized in terms of steric effect, which is less pronounced in receptor **2**, possessing methoxy substituents. As expected, least effective in this series is the single-armed receptor **3**, which recognizes chloride anions almost three times less strongly than receptor **1** with K_TBACl_ = 1.30 × 10^4^ M^−1^. Experiments conducted in the assistance of potassium cations revealed that only ion-pair receptors **1** and **3** can recognize chloride anions in enhanced manner (Table 2), while drop in the value of association constant for receptor **2** lacking crown ether units was noted (K_TBACl_ = 4.09 × 10^4^ M^−1^ vs. K_KCl_ = 3.58 × 10^4^ M^−1^ for receptor **2**). Interestingly, the association constant for the ditopic receptor **3** increased as much as three times upon assistance of potassium cation (K_KCl_ = 3.99 × 10^4^ M^−1^), but **3** is still less effective at ion par recognition than **1**. Furthermore, we found that receptor **1** is also less effective at sodium chloride rather than potassium chloride recognition (K_NaCl_ = 3.94 × 10^4^ M^−1^ vs. K_KCl_ = 4.60 × 10^4^ M^−1^), thus, an extension of the UV-vis binding study was carried out for complexes of **1** with other potassium salts. Specifically we found that **1** can recognize most of the anions in an enhanced manner (including nitrate salts), with cooperativity factor (K_K+_/K_TBA+_) in the range 1.07–1.65. As in the case of titration ^1^H NMR, we have not been able to calculate stability constants for complexes of **1** with sulfates or dihydrogen phosphates due to an inability to fit the titration obtained data to an appropriate binding model.

### 2.3. Extraction Studies

**Figure 3:**
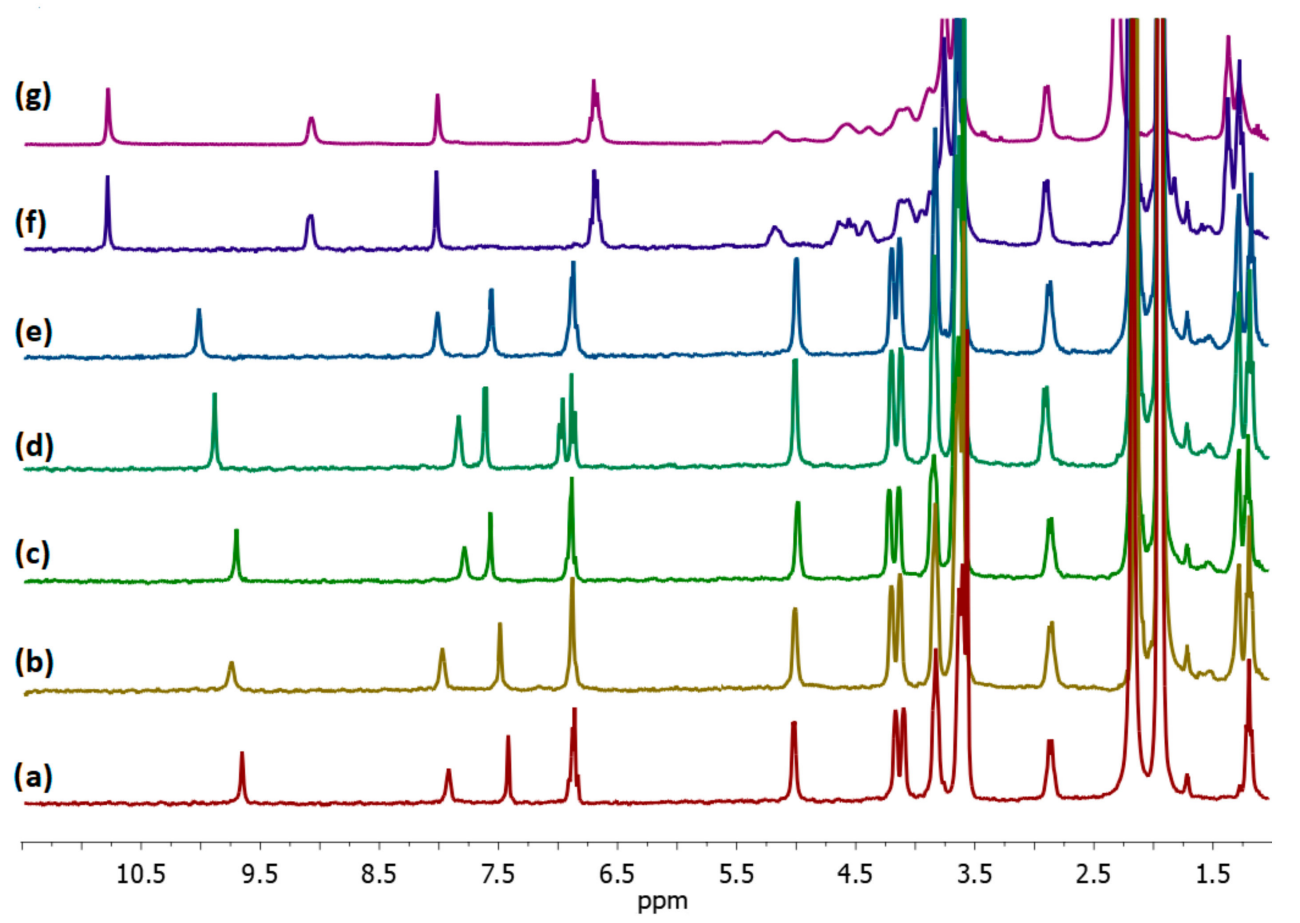
^1^H NMR spectra of receptor **1** recorded in CD_3_CN before (**a**) and after addition of solid salts: (**b**) K_2_SO_4_, (**c**) KN0_3_, (**d**) KBr, (**e**) KCl, (**f**) CH_3_COOK, (**g**) mixture of tested potassium salts.

Quite recently, we found and reported that disparities in binding modes between receptors and selected salts may lead to their selective recognition and extraction [74,75]. Assuming differing stoichiometry of the complexes formed by receptor **1** with sulfates or phosphates and other salts tested, we conducted extraction tests to verify whether the ion-pair receptor **1** can operate under interfacial conditions and is able to differentiate between these salts. Initial reconnaissance in extraction experiments were carried out in solid–liquid extractions (SLE) for combinations of individual salts (KCl, KBr, KNO_3_, CH_3_COOK, PhCOOK, K_2_SO_4_, KH_2_PO_4_). Solution of receptor **1** (5 mM) in CD_3_CN was shaken overnight separately with each solid salt. This process gave rise to new anion complexes in the organic phase, as supported by ^1^H NMR spectroscopic analyses (Figure 3). The ^1^H NMR signals corresponding to complexes are clearly differentiated by the chemical shift of the NH assigned to squaramide protons (Δ δ up to 1.17 ppm) and besides signals corresponding to aromatic protons, those belonging to the crown ether protons were also perturbed, suggesting that potassium cation occupies the crown ether cavity. The most pronounced changes in ^1^H NMR spectrum were noted after SLE using potassium acetate. Specifically, the signals corresponding to the NH squaramide protons, which initially resonated at 9.66 and 7.93 ppm, were shifted downfield to 10.79 and 9.10 ppm, respectively, together with the evident change in the signal pattern of the crown ether protons. After potassium dihydrogen phosphate extraction the signals in ^1^H NMR spectrum were broadened, the signals were in unchanged position and became less intensive, which together with the observation of additional solid found in solution may suggest that the complex formed was not soluble in acetonitrile and precipitated. Interestingly, the ^1^H NMR spectrum recorded after extraction of mixture of tested salts (KCl, KBr, KNO_3_, K_2_SO_4_, KH_2_PO_4_, and CH_3_COOK) with receptor **1** in acetonitrile clearly resembles the spectrum obtained after CH_3_COOK extraction, thus suggesting selectivity under interfacial conditions (SLE) towards this salt. Unfortunately, the overlapping signals corresponding to the CH_3_ group of acetate anion and the residual CH_3_CN solvent prevented determination of the extraction efficiency based on the signal integration. Thus to test the receptor **1** selectivity in SLE experiments and estimate the extraction efficiency for all salts tested, the ion chromatography technique was applied. The solution of receptor **1** (5 mM) in CD_3_CN after vigorous shaking overnight with the salt mixture was filtered, diluted 20-fold with deionized water and analyzed. The obtained data were compared with analogous extraction experiments with pure acetonitrile in which potassium bromide and nitrate are noticeably soluble (Supplementary Materials, Figure S65). The analysis revealed the selectivity of receptor **1** towards potassium acetate, which was extracted with 62% efficiency. The extraction yield promoted by the presence of receptor **1** in acetonitrile solution for other salts did not exceed 5%. Since it was previously reported that a squaramide based ion-pair receptor could solubilize carboxylate salts, we also tested the single armed receptor **3** in SLE conditions and quantified this process [70]. We found that the single armed receptor **3** is less effective and selective in SLE than **1**, what corresponds with the data from titration experiments. Receptor **3** was able to extract potassium acetate and bromide with 30 and 16% yield, respectively.

Then we shifted our attention to liquid–liquid extraction (LLE), tracking the loss of particular anions in the aqueous phase. We carried out extraction experiments using 1 mM of aqueous solution mixture consisting of KCl, KBr, KNO_3_, K_2_SO_4_, CH_3_COOK (1 mM each), and 5 mM solution of receptor **1** in chloroform. After vigorously shaking the two layers for 2 h, the phases were separated and the aqueous phase was analyzed using the ion chromatography technique. Interestingly, under LLE conditions, receptor **1** was found to be selective towards extremely hydrophilic potassium sulfate, for which the drop in concentration was found to be 72%. On the other hand, more lipophilic salts such as acetate, chloride, bromide, and nitrate were extracted less effectively and the drop in concentration in aqueous phase was found to be 38%, 4%, 24%, and 13%, respectively. Control extraction studies for receptors **2** and **3** were then carried out using conditions analogous to those employed for receptor **1**. Receptors **2** and **3** proved to be relatively ineffective as extractants for any of the salts considered in the present study (Supplementary Materials, Table S1). On this basis, we concluded that the combined use of ion pair binding subunits in a three-dimensional molecular architecture lead to a system able to effectively and selectively extract oxyanions. The lack of any of the key features of receptor, such as heteroditopic or tripodal nature, disfavor its effective action under interfacial conditions. Depending on the technique used, SLE or LLE experiments, selective extraction of acetates or sulfates can be achieved, even in the presence of other lipophilic salts, thus overcoming the Hofmeister bias.

### 2.4. Crystallization and Single-Crystal X-ray Diffraction

**Figure 4:**
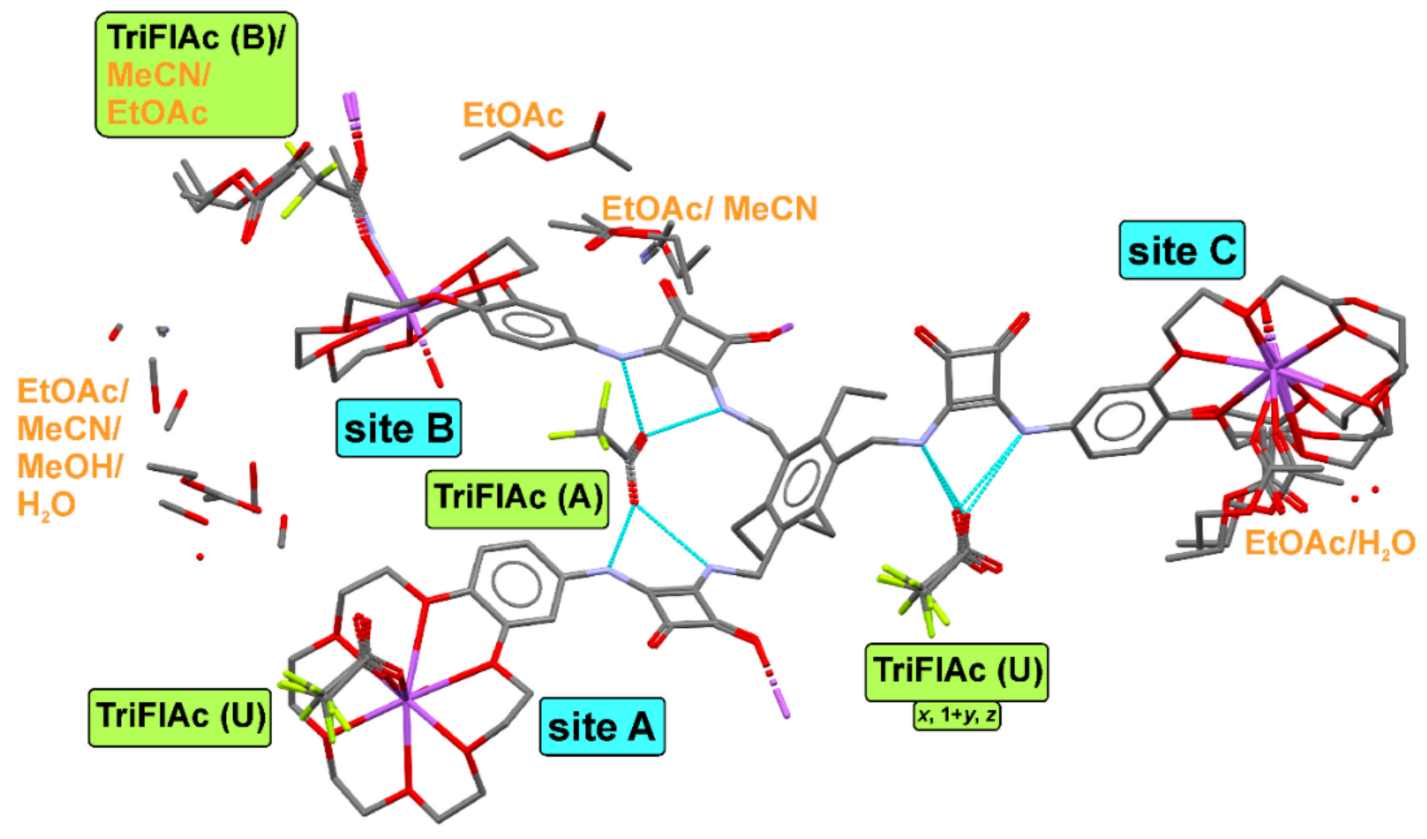
The asymmetric part of the unit cell of the 1 + **Na_triFlAc**, depicting the shape of the ligand, presenting complexation of the anions and showing mixed sites moieties. H atoms omitted for clarity.

Although more light could be shed on the concept of ion pair binding by tripodal receptor **1** and the simultaneous action of its particular arms by single crystal X-ray diffraction experiments, unfortunately all of our attempts to obtain complexes of **1** with the tested salts, including sulfates for which no clear binding mode was established, failed. We were able to obtain the complex (**1** × CF_3_COONa), hereafter abbreviated as **1** + **Na_triFlAc,** as a failed attempt to obtain potassium sulfate complex in acidic conditions. However, the structure is severely disordered; moreover, it contains a non-stoichiometric amount of the salt. The structure crystallizes in the *P*1 space group and in the asymmetric part of the unit cell, it contains one molecule of the tripodal ligand, approximately 2.3 molecules of sodium trifluoroacetate, and number of solvent species, some in non-stoichiometric amount. A general view of all the moieties in the structure is presented in Figure 4.

**Figure 5:**
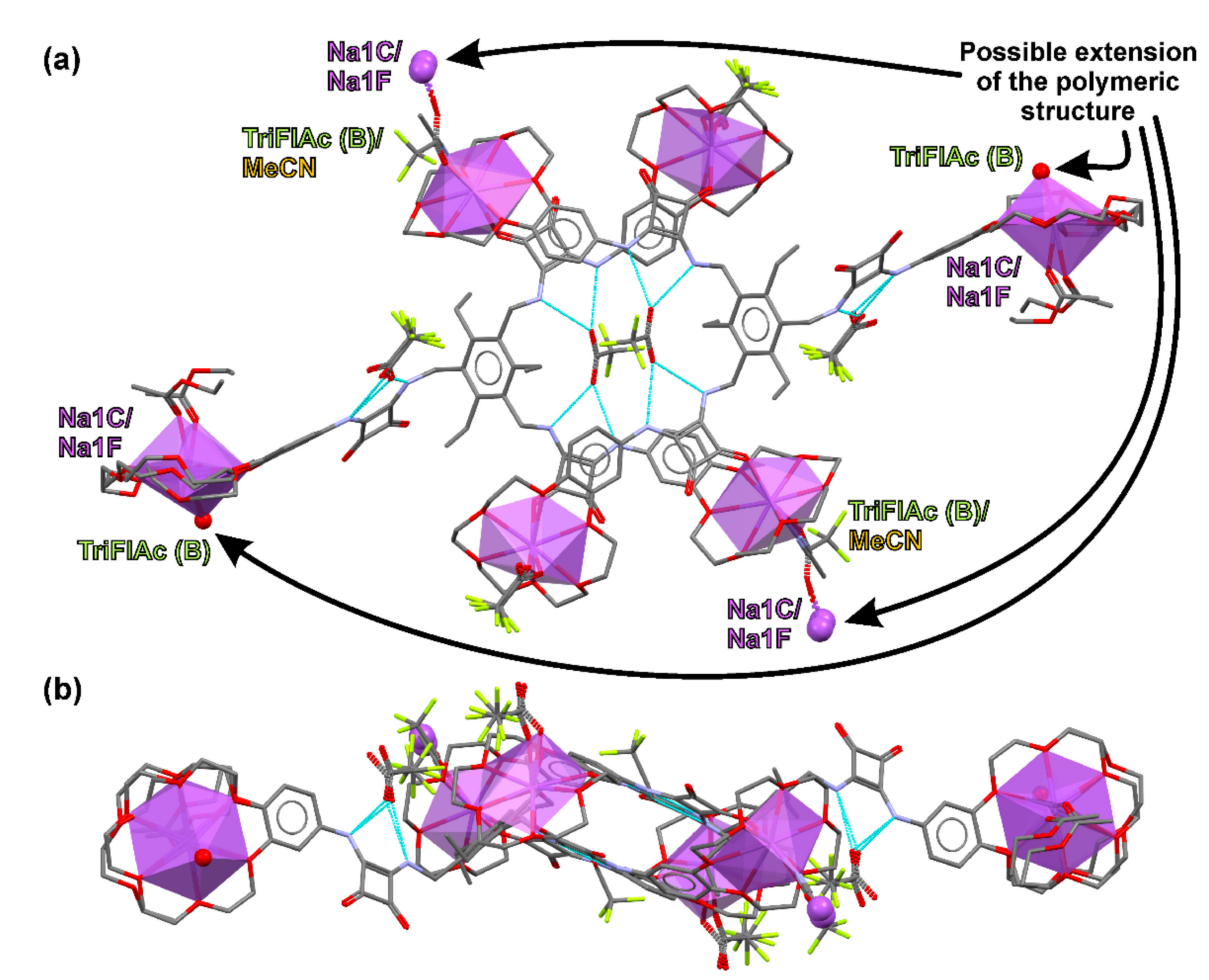
Centrosymmetric dimer in the structure of **1** + **Na_triFlAc**, view from the top (**a**) and rotated view along longer axis of the dimer (**b**). Violet polyhedra represent the coordination of Na^+^ cations by crown ether rings. Hydrogen bonds marked as cyan dashed lines. Unbound solvent molecules and H atoms omitted for clarity.

The ligand coordinates one of the trifluoroacetate anions (TriFlAc (A)) by means of the amide groups located on the two of its branches named “site A” and “site B” in a cooperative way. The other ligand—TriFlAc (U)—is caught by the amide domain of “site C”. This could be an explanation for the partial (2.3 instead of 3) amount of Na + TriFlAc ionic pairs in the structure. Amide binding domains are saturated with ligands; however, the presence of excess sodium in the solution forces the additional complexation of the cation by the crown ether moiety. This excess has to be balanced by some extra amount of TriFlAc anion in the crystal lattice. As there is no space around the amide binding sites, the anion in non-stoichiometric amount (TriFlAc (B)) coordinates Na^+^ cation located in the crown ether ring. The composition of the crystal differs from the 1:1 complexation observed in the solution, where the anion is captured by three arms of the ligand. In the solid state, however, such a complex with planar COO^−^ acceptor group would be rather labile, so formation of more stable open-shaped ligand complexing two anions is achieved. The partial occupancy TriFlAc (B) anion coordinates both the full occupancy Na^+^ cation located at “site B” and the non-stoichiometric amount of sodium ion siting in the “site C” of the neighboring ligand. Such a distribution of ions in the structure results in substitutional disorder. Thus at “site B” Na^+^ cation is coordinated either by 0.3 of TriFlAc (B) or 0.7 of acetonitrile moieties, and is accompanied by a presence of disordered ethyl acetate. Concurrently, at “site C” crown ether ring houses 0.3 of the Na^+^ cation, which is coordinated by the carbonyl group of disordered ethyl acetate moiety. The fractional occurrence of Na^+^ ion at “site C” results in disorder of the aliphatic chain of the crown ether, which is, alternatively, distributed over three locations. In the event that the sodium cation is absent (70% probability), the crown ether ring is bent and is partly filled with methyl groups of the disordered ethyl acetate. In addition, such an empty ring at “site C” also contains a methyl group of the acetonitrile solvent—the one coordinating Na^+^ ion at “site B”. As the ligand contains 18-crown-6 units which are slightly too big for accurate complexation of sodium ligands, the cation moieties are bound not centrally in the ring and the aliphatic parts of the crown ether are somewhat corrugated. Indeed, in the rings containing full occupancy and ordered Na^+^ cations, distances between O and the central ion are 2.58 Å, 2.60 Å, 2.62 Å, 2.63 Å, 2.73 Å, 2.77 Å, and 2.59 Å, 2.59 Å, 2.66 Å, 2.67 Å, 2.80 Å, 2.80 Å for “site A” and “sit-e B”, respectively. All the cations are additionally coordinated by other species (solvent, TriFlAc moieties, or carbonyl groups of neighboring ligand) from both sides of the crown ether rings. Such a coordination by the carbonyl groups of the squaramide moiety leads to the formation of centrosymmetric dimers of ligands. The ligands are additionally supported by parallel π-π interactions between aromatic and squaramide rings, as is presented in Figure 5a,b. In the crystal lattice, the dimers are linked together in higher dimensional structures due to intermolecular hydrogen bonds and bifurcated coordination of Na^+^ cations by TriFlAc anions.

**Figure 6:**
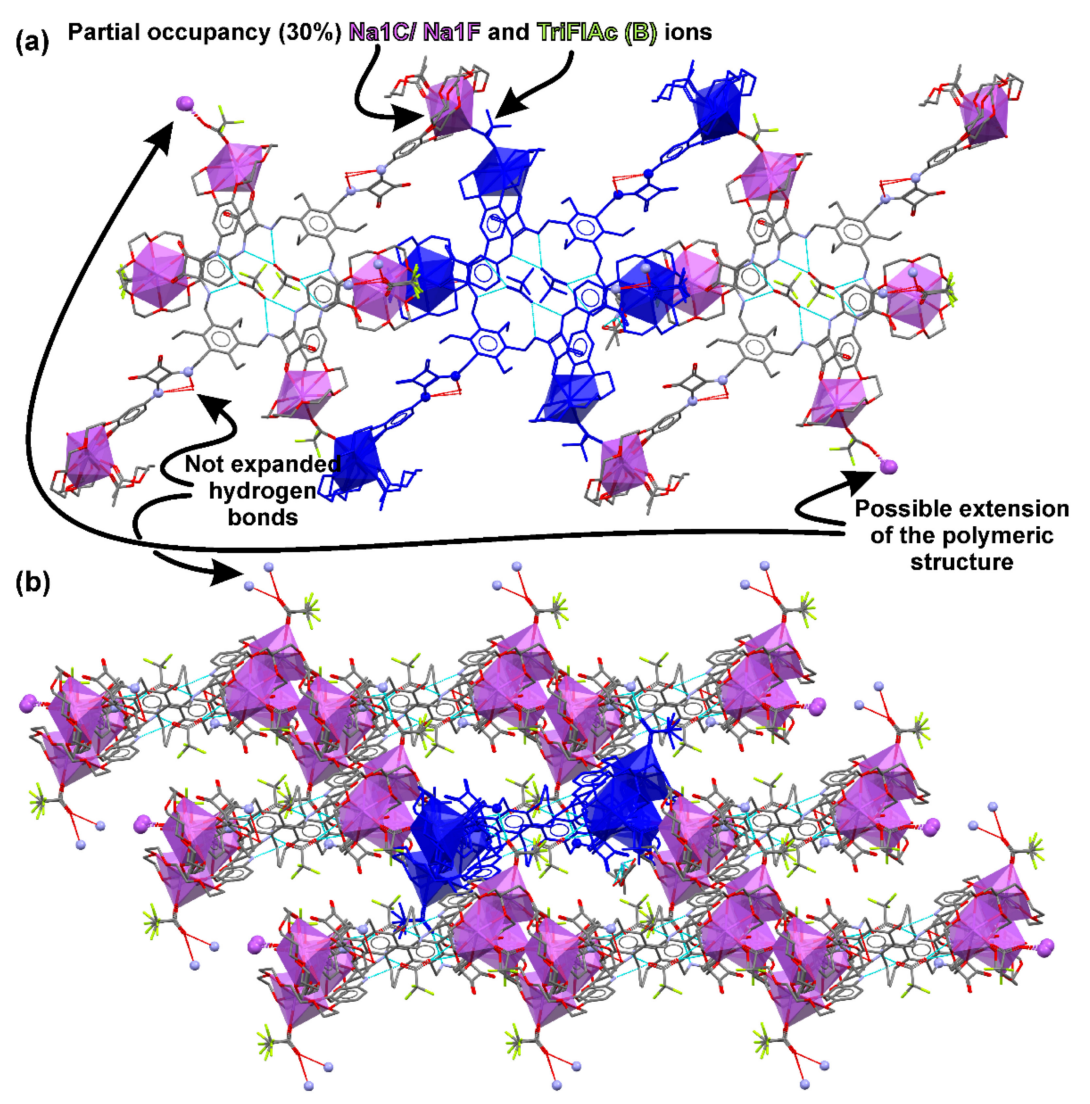
Polymeric structure formation in crystals of **1** + **Na_triFlAc** (**a**) and its extension to two-dimensional (2D) layers thanks to hydrogen bond (**b**). The atoms colored in blue correspond to the centrosymmetric dimer presented in Figure 5. Violet polyhedra represent the coordination of Na^+^ cations by crown ether rings. Hydrogen bonds with displayed complete moieties marked as cyan dashed lines, not expanded hydrogen bonds represented as red dashed lines. Unbound solvent molecules and H atoms omitted for clarity. The presented model assumes 100% presence of TriFlAc (B) anion. In the real case of a non-stoichiometric crystal, the polymeric structure would probably not have been formed due to presence of 70% acetonitrile at mixed site TriFlAc (B)/ CH_3_CN.

However, the extension of the polymeric structure via Na^+^ coordination is only possible in a presence of TriFlAc (B) moiety, which has partial occupancy. Such an option with moieties polymerized in 1-D ribbons along the (110) direction and further grouped together by N-H…O hydrogen bonds in layers parallel to the (001) lattice plane is presented in Figure 6a,b, respectively. In the real, mixed sites, structure the polymeric expansion might be stopped at some nodes due to lack of partial occupancy Na^+^ and TriFlAc ions, which are randomly distributed in the crystal lattice. The TriFlAc (A) anion located between two squaramide domains within one ligand is almost equally bound by the four amide groups with the N…O distances varying in the range 2.80 Å–2.82 Å. This is not the case for the slightly disordered TriFlAc (U) ion, with only one O carbonyl moiety engaged in bifurcated hydrogen bonds. Here the N…O distances are in the range of 2.70 Å–2.90 Å. Another carbonyl oxygen moiety of this anion is engaged in the complexation of the Na^+^ cation trapped in crown ether ring at “site A”.

## 3. Materials and Methods

### 3.1. General Methods

Unless specifically indicated, all other chemicals and reagents used in this study were purchased from commercial sources and used as received. If necessary, purification of products was performed using column chromatography on silica gel (Merck Kieselgel 60, 230–400 mesh) with mixtures of chloroform/methanol. Thin-layer chromatography (TLC) was performed on silica gel plates (Merck Kieselgel 60 F254).

^1^H and ^13^C NMR spectra used in the characterization of products were recorded on Bruker 300 spectrometer (Bruker Corporation, Billerica, MA, USA) using a residual protonated solvent as internal standard. ^1^H NMR DOSY experiments were conducted at 298 K on Varian VNMRS-600 instrument (Varian Inc., Palo Alto, CA, USA) with a residual solvent signal as an internal standard.

High-resolution mass spectra (HRMS) were measured on a LCT (TOF) Micromass unit (Waters Corporation, Milford, CT, USA) using electrospray ionization (ESI) technique.

High performance ion chromatography (HPIC) analyses were performed using a 930 Compact IC Flex apparatus (Metrohm AG, Herisau, Switzerland).

### 3.2. Synthetic Details

**Preparation of (1,3,5-tris(azidomethyl)-2,4,6-triethylbenzene)**: 1,3,5-Tris(azidomethyl)-2,4,6-triethylbenzene was synthesized according to the literature procedure [76] with small modifications. Commercial 1,3,5-tris(bromomethyl)-2,4,6-triethylbenzene (2 g, 4.5 mmol) was dissolved in 20 mL of a DMF/DCM mixture (5:2). A slurry of NaN_3_ (5.3 g, 8.2 mmol) in 5 mL water was added through the top of the condenser to the mixture (the condenser was rinsed with water) which was heated at 80 °C overnight. At this time organic solvents was removed through rotary evaporation, 30 mL of water and 30 mL DCM were added to the crude mixture. The organic phase was separated and washed with water (30 mL). Water phases were combined and washed with DCM (30 mL). Organic phases were combined and dried with Na_2_SO_4_. Evaporation of the dried DCM solution resulted in a gel-like residue which was purified by silica gel column chromatography (25% ethyl acetate in hexane) to give a title compound as a white solid (1.4 g, 0.43 mmol, 96% yield).

HRMS (ESI): calculated for C_15_H_21_N_9_ (M + H) ^+^ 327.1909, found: 327.1915.
^1^H NMR (300 MHz, CDCl_3_) δ 4.50 (s, 6H), 2.84 (q, 6H), 1.25 (t, 9H).
^13^C NMR (75 MHz, CDCl_3_) δ 145.0, 129.8, 47.9, 23.2 15.8.

**Preparation of 1,3,5-tris(aminomethyl)-2,4,6-triethylbenzene.** To a degassed solution of 1,3,5-tris(azidomethyl)-2,4,6-triethylbenzene (1.4 g, 0.43 mmol) in 40 mL of a THF/MeOH mixture (1:4), 15 mg of 10% Pd/C was added. The reaction mixture was kept under a H_2_ atmosphere (balloon pressure) at room temperature overnight. The mixture was filtered quickly through celite and the filtrate was evaporated close to dryness under reduced pressure to give the crude product in near quantitative yield (1.0g, 0.42 mmol).

HRMS (ESI): calculated for C_15_H_27_N_3_ (M + H) ^+^ 250.2283, found: 250.2290.
^1^H NMR (300 MHz, CDCl_3_) δ 3.85 (s, 6H), 2.80 (q, 6H), 1.40 (s, 6H), 1.25 (t, 9H).
^13^C NMR (75 MHz, CDCl_3_) δ 140.4, 137.5, 39.7, 22.5, 16.8.

**Preparation of module 4.** A 10% palladium on carbon catalyst 25 mg was added to a degassed solution of 4-nitrobenzo-18-crown-6 ether (2.05 g, 5.74 mmol) in 70 mL of a THF/MeOH mixture (1:4). The reaction mixture was stirred under a H_2_ atmosphere (balloon pressure) at room temperature overnight. The catalyst was removed by filtration through a pad of Celite and washed with MeOH (30 mL). The filtrate was concentrated under reduced pressure to give the crude product in a quantitative yield (1.87 g, 5.72 mmol). The obtained 4-aminobenzo-18-crown-6 ether was used in the next step without further purification. To the 4-aminobenzo-18-crown-6 ether solution of (1.87 g, 5.72 mmol) in MeOH (20 mL), 3,4-dimethoxy-3-cyclobutene-1,2-dione (0.81 g, 5.72 mmol) was added. The reaction mixture was stirred at room temperature overnight. The resulting precipitate was isolated by filtration and obtained solid material was washed several times with methanol. The obtained white solid was dried in vacuo to give desired product (1.75 g, 4.01 mmol, 70% yield).

HRMS (ESI): calculated for C_21_H_27_NO_9_Na (M + Na)^+^: 460.1584, found: 460.1588.
^1^H NMR (300 MHz, DMSO-*d_6_*) δ 10.64 (s, 1H), 7.18–6.96 (m, 1H), 6.96–6.84 (m, 1H), 6.83–6.78 (m, 1H), 4.34 (s, 3H), 4.10–3.95 (m, 4H), 3.80–3.65 (m, 4H), 3.62–3.44 (m, 12H).
^13^C NMR (75 MHz, DMSO-*d_6_*) δ 189.3, 184.1, 178.9, 169.1, 148.9, 145.6, 132.0, 114.0, 112.1, 106.7, 70.5, 70.3, 70.2, 69.6, 69.4, 69.1, 68.7, 61.0.

**Preparation of module 5.** To a solution of the 3,4-dimethoxy-3-cyclobutene-1,2-dione (0.93 g, 6.5 mmol) in MeOH (10 mL) were added 3,4-dimetoxyaniline (1 g, 6.5 mmol) and DIPEA (1.7 mL, 9.75 mmol), the mixture was stirred overnight at ambient temperature. Then the reaction mixture was concentrated and purified by silica gel column chromatography (2% methanol in chloroform) to give module 5 as a yellow solid (1.3g, 5.0 mmol, 77% yield).

HRMS (ESI): calculated for C_13_H_13_NO_5_Na (M + Na)^+^: 286.0692, found: 286.0702.
u^1^H NMR (300 MHz, DMSO-*d_6_*) δ 10.63 (s, 1H), 7.20–6.65 (m, 3H), 4.37 (s, 3H), 3.85–3.65 (m, 6H).
^13^C NMR (75 MHz, DMSO-*d_6_*) δ 189.1, 183.8, 178.6, 169.2, 149.4, 146.2, 131.8, 112.6, 111.9, 105.3, 60.9, 56.2, 55.9.

**Preparation of receptor 1.** To a solution of 1,3,5-tris(aminomethyl)-2,4,6-triethylbenzene (270 mg, 1.1 mmol) in MeOH (15 mL), module 4 (1.49 g, 3.4 mmol), Et_3_N (0.68 mL, 5 mmol) were added. After it was stirred overnight at ambient temperature, the reaction mixture was concentrated and purified by silica gel column chromatography (5% to 20% methanol in chloroform) to give receptor 1 as a yellow solid (0.84 g, 0.57 mmol, 52% yield).

HRMS (ESI): calculated for C_75_H_96_N_6_O_24_Na (M + Na)^+^:1487.6373, found: 1487.6333.
^1^H NMR (300 MHz, DMSO-*d_6_*) δ 9.43 (s, 1H), 7.59 (s, 1H), 7.30 (s, 1H), 6.92–6.73 (m, 2H), 4.98 (s, 2H), 4.12–4.00 (m, 4H), 3.80–3.72 (m, 4H), 3.62–3.50 (m, 12H), 2.85 (s, 2H), 1.19 (m, 3H).
^13^C NMR (75 MHz, DMSO-*d_6_*) δ 183.8, 180.7, 168.5, 163.9, 148.6, 144.4, 144.0, 133.1, 132.5, 113.3, 110.0, 104.3, 70.0, 69.9, 69.0, 68.8, 67.9, 67.7, 65.4, 42.0, 23.1, 16.8.

**Preparation of receptor 2:** 1,3,5-Tris(aminomethyl)-2,4,6-triethylbenzene (220 mg, 0.88 mmol) was reacted with module 5 (695 mg, 2.64 mmol) according to procedure described for the receptor **1** to yield receptor **2** as a white solid (499 mg, 0.53 mmol, 60% yield).

HRMS (ESI): calculated for C_51_H_54_N_6_O_12_Na (M + Na)^+^: 965.3698, found: 965.3712.
^1^H NMR (300 MHz, DMSO-*d_6_*) δ 9.44 (s, 1H), 7.59 (s, 1H), 7.31 (s, 1H), 6.94-6.76 (m, 2H), 4.99 (s, 2H), 3.79–3.68 (m, 6H), 2.88–2.84 (m, 2H), 1.19 (m, 3H).
^13^C NMR (75 MHz, DMSO-*d_6_*) δ 183.7, 180.7, 168.4, 163.9, 149.8, 145.1, 144.3, 133.1, 132.6, 113.1, 109.9, 103.9, 56.3, 55.9, 42.0, 23.1, 16.8.

**Preparation of receptor 3.** To a solution of benzylamine (49 mg, 0.46 mmol) in MeOH (5 mL), module 4 (200 mg, 0.46 mmol) was added and the mixture was stirred at room temperature overnight. Then the reaction mixture was filtered, and the collected solid material was washed with MeOH. The obtained white solid was dried in vacuo to give the desired receptor (170 mg, 0.33 mmol, 72% yield).

HRMS (ESI): calculated for C_27_H_32_N_2_O_8_Na (M + Na)^+^: 535.2056, found: 535.2043.
^1^H NMR (300 MHz, DMSO-*d_6_*) δ 9.56 (s, 1H), 7.90 (s, 1H), 7.45–7.30 (m, 5H), 7.23 (s, 1H), 7.00–6.80 (m, 1H), 6.80–6.71 (m, 1H), 4.80 (s, 2H), 4.12–3.95 (m, 4H), 3.79–3.68 (m, 4H), 3.66–3.49 (m, 12 H).
^13^C NMR (75 MHz, DMSO-*d_6_*) δ 183.9, 181.2, 168.9, 164.2, 149.2, 144.6, 139.0, 133.1, 129.2, 128.1, 128.1, 114.5, 110.4, 105.1, 70.3, 69.2, 69.1, 69.0, 68.4, 47.6.

### 3.3. NMR Titration Procedure

The ^1^H NMR titration was conducted at 298K in DMSO-*d_6_*. In each case, a 500 μL of freshly prepared 1.7 mM solution of receptor **1** (1.4 mM of receptor **2**; 2.0 mM of receptor **3**) was added to a 5 mm NMR tube. In the case of ion pair, titration receptor was firstly pretreated with one or three equivalents of NaClO_4_ or KPF_6_. Then small aliquots of solution of TBAX, containing receptor at constant concentration, were added and a spectrum was acquired after each addition. The resulting titration data were analyzed using BindFit (v0.5) package, available online at http://supramolecular.org.

### 3.4. UV-Vis Titration Procedure

UV-vis titration experiments were performed on in CH_3_CN solution at 298K. To 10 mm cuvette was added 2.5 mL of freshly prepared (receptor **1**: c = 1.2 × 10^−5^ M, receptor **2**: c = 1.4 × 10^−5^ M, receptor **3**: c = 2.1 × 10^−5^ M) solution of studied receptor and in case of ion pair binding studies 1 mol equivalent of cation (KPF_6_ or NaClO_4_) was added prior titrations. Small aliquots of ca. 1.1 × 10^−3^ M TBAX solution containing receptor **1**, receptor **2** or receptor **3** at the same concentration as in cuvette, were added and a spectrum was acquired after each addition. The resulting titration data were analyzed using BindFit (v0.5) package, available online at http://supramolecular.org.

### 3.5. Crystallographic Measurements

The crystal was measured on the Bruker D8 Venture diffractometer (Bruker AXS, Inc., Madison, WI, USA) at 130.0(5) K [78,79,80]. The structure was solved and refined using SHELXTL Software Package (Bruker AXS, Inc., Madison, WI, USA) [81,82] based on atomic scattering factors taken from the International Tables [83]. The Molecular graphics was prepared using Mercury CSD 2020 program [84]. The details concerning X-ray diffraction experiment and crystal structure refinement are located in Table 3. Cambridge Crystallographic Data Centre (CCDC) 2038741 contains the supplementary crystallographic data for this paper. The data can be obtained free of charge from The Cambridge Crystallographic Data Centre via www.ccdc.cam.ac.uk/structures.

## 4. Conclusions

To summarize, a series of molecular receptors able to interact with ions were synthesized and studied using standard spectroscopic protocols as well as by single X-ray crystal diffraction analysis. By utilizing the spatial preorganization of the binding sites, the high potency of squaramide units in anion binding, as well as the enhancement of anion binding by the presence of cations, a highly effective and selective tripodal ion-pair receptor **1** was obtained. Comparative binding studies of reference receptors **2** and **3** demonstrated the indispensability of individual structural elements of receptor **1**, i.e., its tripodal and heteroditopic features. The lack of heteroditopic character in receptor **2** or tripodal arrangement in receptors **3** structures resulted in inefficiency in operation under interfacial liquid–liquid conditions. The high potency of receptor **1** to interact with ion pairs was utilized in liquid–liquid extraction experiments, and demonstrated its ability to extract extremely hydrophilic sulfate anions from aqueous to organic phase, and do so selectively in the presence of other lipophilic anions, thus, overcoming Hofmeister series. Changing the extraction conditions from LLE to SLE (solid–liquid extraction) resulted in switching the selectivity of **1** from sulfates towards acetates.

## Figures and Tables

**Table 1:** Association constants (Ka) for interactions between receptor **1** and selected anions in the absence or presence of 3 equivalent of KPF_6_^(a)^.

	1	1 + 3 Equivalent K+	K_K+_/K_TBA-_
**Cl^−^**	283	373	1.32
**Br^−^**	40	100	2.50
**NO_2_^−^**	59	101	1.71
**NO_3_^− (b)^**	−	−	-
**PhCOO^−^**	415	500	1.20
**CH_3_COO^−^**	400	453	1.13
**SO_4_^2− (c)^**	−	-	-
**H_2_PO_4_^− (c)^**	-	-	-

^(a) 1^H NMR, solvent DMSO-*d6*, temperature 298 K, c = 1.7 × 10^−3^ M; anions added as TBA salts ∼0.6 M; data fitted to 1:1 binding model. Errors <10%, see Supplementary Materials. Stability constants for receptor **1**: K = 324 M^−1^ for Cl^−^ in the presence of 3 equivalents of Na^+^. Stability constants for receptor **2** (c = 1.4 × 10^−3^ M): K = 292 M^−1^ for Cl^−^; K = 277 M^−1^ for Cl^−^ in the presence of 3 equivalents of K^+^. Stability constants for receptor **3** (c = 2.0 × 10^−3^ M): K = 123 M^−1^ for Cl^−^; K = 184 M^−1^ for Cl^−^ in the presence of 1 equivalent K^+^. ^(b)^ Low changes in NMR spectrum upon titration to determine Ka. ^(c)^ The data obtained could not be fitted to appropriate binding model.

**Table 2:** Association constants (Ka) for interactions between receptor **1** and selected anions in the absence or presence of 3 equivalent of KPF_6_^(a)^.

	1	1 + 3 equiv. K+	K_K+_/K_TBA−_
**Cl^−^**	3.68 × 10^4^	4.60 × 10^4^	1.25
**Br^−^**	2.27 × 10^4^	3.61 × 10^4^	1.60
**NO_2_^−^**	2.16 × 10^4^	3.55 × 10^4^	1.65
**NO_3_^−^**	5.26 × 10^3^	7.84 × 10^3^	1.49
**PhCOO^−^**	8.40 × 10^4^	9.02 × 10^4^	1.07
**CH_3_COO^−^**	6.14 × 10^4^	7.59 × 10^4^	1.24
**SO_4_^2− (b)^**	-	-	-
**H_2_PO_4_^− (b)^**	-	-	-

^(a)^ UV-vis, Solvent CH_3_CN, temperature 298 K, c = 1.2 × 10^−5^ M; anions added as TBA salts ∼1.1 × 10^−3^ M, data fitted to 1:1 binding model. Errors <10%, see Supplementary Materials. Stability constants for receptor **1**: K = 3.94 × 10^4^ M^−1^ for Cl^−^ in the presence of 3 equivalents of Na^+^. Stability constants for receptor **2** (c = 1.4 × 10^−5^ M): K = 4.09 × 10^4^ M^−1^ for Cl^−^. K = 3.58 × 10^4^ M^−1^ for Cl^−^ in the presence of 3 equivalents of K^+^. Stability constants for receptor **3** (c = 2.1 × 10^−5^ M): K = 1.30 × 10^4^ M^−1^ for Cl^−^; K = 3.99 × 10^4^ M^−1^ for Cl^−^ in the presence of 1 equivalent of K^+^. ^(b)^ The data obtained could not be fitted to appropriate binding model.

**Table 3:** Data collection and structure refinement for **1 + Na_triFlAc**.

Formula	C_102.01_H_140.02_F_6.91_N_7.59_Na_2.30_O_39.28_, corresponding to approx.: 1 + 2.30 × (sodium trifluoroacetate) + 4.61 × (ethyl acetate) + 1.58 × acetonitrile + 0.80 × methanol * + 0.65 × H_2_O*
***M_x_*/g∙mol^−1^**	2288.94
***T*/K**	130.0(5)
***λ*/Å**	0.71073
**Crystal size**	0.086 × 0.307 × 0.569
**Space group**	*P* 1
**Unit cell dimensions**	*a* = 13.9030(10) Å *α* = 106.491(2)° *b* = 19.1752(13) Å *β* = 98.390(2)° *c* = 24.2017(16) Å *γ* = 105.570(2)°
***V*/Å^3^, *Z***	5784.7(7), 2
***D_x_*/g∙cm^−3^**	1.312
***μ*/mm^−1^**	0.114
***F*(000)**	2414
***θ_min_*, *θ_max_***	2.86°, 25.05°
**Index ranges (merged data)**	−16 ≤ *h* ≤ 16, −23 ≤ *k* ≤ 22, 0 ≤ *l* ≤ 29
**Reflections collected/independent**	208133/ 21096 ** R_int_ = 0.0674 **
**Completeness**	99.1%
**Absorption correction**	Multi-Scan
***T_max_*, *T_min_***	0.990, 0.938
**Structure solution technique**	direct methods
**Refinement method**	Full-matrix LSQ on *F*^2^
**Data/restraints/parameters**	20322/603/1807
**GOF on *F*^2^**	1.051
**Final *R* indices**	13630 data; *I* > 2*σ*(*I*) *R*1 = 0.0699, *wR*2 = 0.1894 all data *R*1 = 0.1085, *wR*2 = 0.2084
**Extinction coefficient**	0.0013(4)
**Δ*ρ_max_*, Δ*ρ_min_***	0.397, −0.396 e∙Å^−3^

* Oxygen moieties with no hydrogen atoms assigned. ** Values based on data scaling corresponding to 2*θ* = 50.96°.

## Supplementary Materials

Supplementary materials can be found at https://www.mdpi.com/1422-0067/21/24/9465/s1.

## References

[1] Evans N.H., Beer P.D. (2014). Advances in Anion Supramolecular Chemistry: From Recognition to Chemical Applications. Angew. Chem. Int. Ed..

[2] Busschaert N., Caltagirone C., Van Rossom W., Gale P.A. (2015). Applications of supramolecular anion recognition. Chem. Rev..

[3] Carter K.P., Young A.M., Palmer A.E. (2014). Fluorescent sensors for measuring metal ions in living systems. Chem. Rev..

[4] Gale P.A. (2011). From anion receptors to transporters. Acc. Chem. Res..

[5] He Q., Seung G.I.V., Kim H., Kim S.K., Sessler J.L. (2019). Macrocycles as Ion Pair Receptors. Chem. Rev..

[6] Kneeland D.M., Ariga K., Lynch V.M., Huang C.Y., Anslyn E.V. (1993). Bis(Alkylguanidinium) Receptors for Phosphodiesters—Effect of Counterions, Solvent Mixtures, and Cavity Flexibility on Complexation. J. Am. Chem. Soc..

[7] Shukla R., Kida T., Smith B.D. (2000). Effect of competing alkali metal cations on neutral host’s anion binding ability. Org. Lett..

[8] Arduini A., Brindani E., Giorgi G., Pochini A., Secchi A. (2002). Anion effects on the recognition of ion pairs by calix[4]arene-based heteroditopic receptors. J. Org. Chem..

[9] Bohmer V., Cort A.D., Mandolini L. (2001). Counteranion effect on complexation of quats by a neutral calix[5]arene receptor. J. Org. Chem..

[10] Berthault P., Desvaux H., Wendlinger T., Gyejacquot M., Stopin A., Brotin T., Dutasta J.P., Boulard Y. (2010). Effect of pH and Counterions on the Encapsulation Properties of Xenon in Water Soluble Cryptophanes. Chem. Eur. J..

[11] Kim S.K., Sessler J.L. (2010). Ion pair receptors. Chem. Soc. Rev..

[12] McConnell A.J., Beer P.D. (2012). Heteroditopic receptors for ion-pair recognition. Angew. Chem. Int. Ed..

[13] Zdanowski S., Romański J. (2015). Ion pair binding by an L-tyrosine-based polymerizable molecular receptor. N. J. Chem..

[14] Gale P.A., Busschaert N., Haynes C.J.E., Karagiannidis L.E., Kirby I.L. (2014). Anion receptor chemistry: Highlights from 2011 and 2012. Chem. Soc. Rev..

[15] Gale P.A., Caltagirone G. (2015). Anion sensing by small molecules and molecular ensembles. Chem. Soc. Rev..

[16] Martínez-Máñez R., Sancenón F. (2003). Fluorogenic and Chromogenic Chemosensors and Reagents for Anions. Chem. Rev..

[17] Pletnev I., Gloe K. (2005). Macrocyclic Chemistry: Current Trends and Future Perspectives.

[18] Kuswandi B., Nuriman N., Verboom W., Reinhoudt D.N. (2006). Tripodal Receptors for Cation and Anion Sensors. Sensors.

[19] Berocal M.J., Cruz A., Badr I.H.A., Bachas L.G. (2000). Tripodal ionophore with sulphate recognition properties for anion-selective electrode. Anal. Chem..

[20] Ravikumar I., Ghosh P. (2012). Recognition and separation of sulfate anions. Chem. Soc. Rev..

[21] Dey S.K., Basu A., Chutia R., Das G. (2016). Anion coordinated capsules and pseudocapsules of tripodal amide, urea and thiurea scaffolds. RSC Adv..

[22] Sato K., Arail S., Yamagishi T.A. (1999). New tripodal anion receptor with C-H—X hydrogen bonding. Tetrahedron Lett..

[23] Ballester P., Costa A., Deya P.M., Vega M., Morey J. (1999). Influence of remote intramolecular hydrogen bonds on the thermodynamics of molecular recognition of *cis*-1,3,5-cyclohexanetricarboxylic acid. Tetrahedron Lett..

[24] Fan A.L., Hong H.K., Valiyaveettil S., Vittal J.J. (2002). A urea-incorporated receptor for aromatic carboxylate anion recognition. J. Supramol. Chem..

[25] Schmuck C., Schwegmann M. (2005). A Molecular Flytrap for the Selective Binding of Citrate and Other Tricarboxylates in Water. J. Am. Chem. Soc..

[26] Li Z., Yu X.-H., Chen Y., Yuan D.-Q., Chen W.-H. (2017). Synthesis, Anion Recognition, and Transmembrane Anionophoric Activity of Tripodal Diaminocholoyl Conjugates. J. Org. Chem..

[27] Hennrich G., Anslyn E.V. (2002). Novel C_3_-Symmetric Molecular Scaffolds with Potential Facial Differentiation. Chem. Eur. J..

[28] Jonah T.M., Mathivathanan L., Morozov A.N., Mebel A.M., Raptis R.G., Kavallieratos K. (2017). Remarkably selective NH4+ binding and fluorescence sensing by tripodal tris(pyrazolyl) receptors derived from 1,3,5-triethylbenzene: Structural and theoretical insights on the role of ion pairing. N. J. Chem..

[29] Schulze M.M., Koch N., Seicheter W., Mazik M. (2018). Crystalline ammonium complexes of Trimethyl- and triethylbenzene-based tripodal compounds bearing pyrazole and indazole groups. Eur. J. Org. Chem..

[30] Tomas S., Prohens R., Vega M., Rotger M.C., Deya P.M., Ballester P., Costa A. (1996). Squaramido-based receptors: Design, synthesis, and application to the recognition of tetraalkylammonium compounds. J. Org. Chem..

[31] Hao J., Hiratani K., Kameta N., Oba T. (2009). Synthesis of a novel tripodal having 3-hydroxy-2-naphthoeic amide groups and its anion recognition ability. J. Incl. Phenom. Macrocycl. Chem..

[32] Turner D.R., Paterson M.J., Steed J.W. (2006). A conformationally flexible, urea-based tripodal anion receptor: Solid-state, solution, and theoretical studies. J. Org. Chem..

[33] Pandurangan K., Kitchen J.A., Blasco S., Boyle E.M., Fitzpatrick B., Feeney M., Kruger P.E., Gunnlaugsson T. (2015). Unexpected self-sorting self-assembly formation of a [4:4] sulfate: Ligand cage from a preorganized tripodal urea ligand. Angew. Chem. Int. Ed..

[34] Valkenier H., Dias C.M., Porter Goff K.L., Jureček O., Puttreddy R., Rissanen K., Davis A.P. (2015). Sterically geared tris-thiureas; transmembrane chloride transporters with unusual activity and accessibility. Chem. Commun..

[35] Oh J.H., Kim J.H., Kim D.S., Han H.J., Lynch V.M., Sessler J.L., Kim S.K. (2019). Synthesis and Anion Recognition Features of a Molecular Cage Containing Both Hydrogen Bond Donors and Acceptors. Org. Lett..

[36] Hong S.-J., Yoo J., Jeong S.-D., Lee C.-H. (2010). Convenient synthesis of tripodal-pyrrole receptor and anion binding properties. J. Incl. Phenom. Macrocycl. Chem..

[37] Bill N.L., Kim D.S., Kim S.K., Park J.S., Lynch V.M., Young N.J., Hay B.P., Yang Y., Anslyn E.V., Sessler J.L. (2012). Oxoanion recognition by benzene-based tripodal pyrrolic receptors. Supramol. Chem..

[38] Tomàs S., Prohens R., Deslongchamps G., Ballester P., Costa A. (1999). An effective fluorescent sensor for choline-containing phospholipids. Angew. Chem. Int. Ed..

[39] Soberats B., Martínez L., Sanna E., Sampedro A., Rotger C., Costa A. (2012). Janus-like Squaramide-based hosts: Dual mode of binding and conformational transitions driven by ion-pair recognition hosts dual mode of binding and conformational transitions driven by ion-pair recognition. Chem. Eur. J..

[40] Jain A., Jain Y., Gupta R., Agarwal M. (2018). Trifluoromethyl group containing C3 symmetric coumarin-triazole based fluorometric tripodal receptors for selective fluoride ion recognition: A theoretical and experimental approach. J. Fluor. Chem..

[41] Brynatsev V.S., Hay B.P. (2005). Influence of substituents on the strength of aryl C-H···Anion hydrogen bonds. Org. Lett..

[42] Esipenko N.A., Koutnik P., Minami T., Mosca L., Lynch V.M., Zyryanov G.V., Anzenbacher P. (2013). First supramolecular sensors for phosphonate anions. Chem. Sci..

[43] Minami T., Liu Y., Akdeniz A., Koutnik P., Esipenko N.A., Nishiyabu R., Kubo Y., Anzenbacher P. (2014). Interamolecular indicator displacement assay for anions: Supramolecular sensor for glyphosate. J. Am. Chem. Soc..

[44] Metzger A., Anslyn E.V. (1998). A Chemosensor for Citrate in Beverages. Angew. Chem. Int. Ed..

[45] Lee M., Zali-Boeini H., Li F., Lindoy L.F., Jolliffe K.A. (2013). Synthesis of tris-(azacrown) ethers for carboxylic acid recognition. Tetrahedron.

[46] Turner D.R., Paterson M.J., Steed J.W. (2008). Conformational control by ‘zipping-up’ an anion-binding unimolecular capsule. Chem. Commun..

[47] Arunachalam M., Ghosh P. (2009). Recognition and complexation of hydrated fluoride anion: F_2_(H_2_O)_6_^2-^ templated formation of a dimeric capsule of a tripodal amide. Chem. Commun..

[48] Arunachalam M., Ghosh P. (2009). Formation of a nitrate zipped dimeric capsule and un-zipping by chloride doping. Chem. Commun..

[49] Beer P.D., Hopkins P.K., McKinney J.D. (1999). Cooperative halide, perrhenate anion-sodium cation binding and pertechnetate extraction and transport by a novel tripodal tris(amido benzo-15-crown-5) ligand. Chem. Commun..

[50] Marchetti L.A., Kumawat L.K., Mao N., Stephens J.C., Elmes R.B.P. (2019). Versatility of Squaramides: From Supramolecular Chemistry to Chemical Biology. Chem.

[51] Wurm F.R., Klok H.-A. (2013). Be squared: Expanding the Horizon of Squaric Acid-Mediated Conjugations. Chem. Soc. Rev..

[52] Storer R.I., Aciro C., Jones L.H. (2011). Squaramides: Physical Properties, Synthesis and Applications. Chem. Soc. Rev..

[53] Jagleniec D., Siennicka S., Dobrzycki Ł., Karbarz M., Romański J. (2018). Recognition and extraction of sodium chloride by a squaramide-based ion pair receptor. Inorg. Chem..

[54] Rotger M.C., Piña M.N., Frontera A., Martorell G., Ballester P., Deya P.M., Costa A. (2004). Conformational preferences and self-template macrocyclization of squaramide-based foldable modules. J. Org. Chem..

[55] Rotger C., Soberats B., Quiñonero D., Frontera A., Ballester P., Benet-Buchholz J., Deya P.M., Costa A. (2008). Crystallographic and theoretical evidence of anion–p and hydrogen-bonding interactions in a squaramide–nitrate salt. Eur. J. Org. Chem..

[56] López C., Ximenis M., Orvay F., Rotger C., Costa A. (2017). Supramolecular hydrogels based on minimalist amphiphilic squaramide–squaramates for controlled release of zwitterionic biomolecules. Chem. Eur. J..

[57] Martínez-Crespo L., Escudero-Adán E.C., Costa A., Rotger C. (2018). The role of N-methyl squaramides in a hydrogenbonding strategy to fold peptidomimetic compounds. Chem. Eur. J..

[58] Martínez L., Martorell G., Sampedro A., Ballester P., Costa C., Rotger C. (2015). Hydrogen bonded squaramide-based foldable module induces both b- and a-turns in hairpin structures of a-peptides in water. Org. Lett..

[59] Olmo F., Rotger C., Ramírez-Macías I., Martínez L., Marín C., Carreras L., Urbanová K., Vega M., Chaves-Lemaur G., Sampedro A. (2014). Synthesis and biological evaluation of N,N’-squaramides with high in vivo efficacy and low toxicity: Toward a lowcost drug against Chagas disease. J. Med. Chem..

[60] Amendola V., Bergamaschi G., Boiocchi M., Fabbrizzi L., Milani M. (2010). The Squaramide versus Urea Contest for Anion Recognition. Chem. Eur. J..

[61] Cai X.-J., Li Z., Chen W.-H. (2018). Synthesis, Anion Recognition and Transmembrane Anion-Transport Properties of Squaramides and Their Derivatives. Mini-Rev. Org. Chem..

[62] Gale P.A., Davis J.T., Quesada R. (2017). Anion transport and supramolecular medicinal chemistry. Chem. Soc. Rev..

[63] Yang Y., Wu X., Busschaert N., Furuta H., Gale P.A. (2017). Dissecting the Chloride-Nitrate Anion Transport Assay. Chem. Commun..

[64] Busschaert N., Kirby I.L., Young S., Coles S.J., Horton P.N., Light M.E., Gale P.A. (2012). Squaramides as Potent Transmembrane Anion Transporters. Angew. Chem. Int. Ed..

[65] Cai X.-J., Li Z., Chen W.-H. (2017). Tripodal Squaramide Conjugates as Highly Effective Transmembrane Anion Transporters. Bioorganic Med. Chem. Lett..

[66] Wu X., Judd L.W., Howe E.N.W., Withecombe A.M., Soto-Cerrato V., Li H., Busschaert N., Valkenier H., Pérez-Tomás R., Sheppard D.N. (2016). Nonprotonophoric Electrogenic Cl^−^ Transport Mediated by Valinomycin-like Carries. Chem.

[67] Busschaert N., Park S.-H., Baek K.-H., Choi Y.P., Park J., Howe E.N.W., Hiscock J.R., Karagiannidis L.E., Marques I., Félix V. (2017). A Synthetic Ion Transporter that Disrupts Autophagy and Induces Apoptosis by Perturbing Cellular Chloride Concentrations. Nat. Chem..

[68] Elmes R.B.P., Yuen K., Jolliffe K.A. (2014). Sulfate-Selective Recognition by Using Neutral Dipeptide Anion Receptors in Aqueous Solution. Chem. Eur. J..

[69] Qin L., Hartley A., Turner P., Elmes R.B.P., Jolliffe K.A. (2016). Macrocyclic squaramides: Anion receptors with high sulfate binding affinity and selectivity in aqueous media. Chem. Sci..

[70] Frontera A., Orell M., Garau C., Quiñonero D., Molins E., Mata I., Morey J. (2005). Preparation, Solid-State characterization, and Computational Study of a Crown Ether Attached to a Squaramide. Org. Lett..

[71] Załubiniak D., Zakrzewski M., Piątek P. (2016). Highly Effective Ion-Pair Receptors Based on 2,2-Bis(aminomethyl)-propionic Acid. Dalton Trans..

[72] Zdanowski S., Piątek P., Romański J. (2016). An Ion Pair Receptor Facilitating the Extraction of Chloride Salt from the Aqueous to the Organic Phase. N. J. Chem..

[73] Zaleskaya M., Jagleniec D., Karbarz M., Dobrzycki Ł., Romański J. (2020). Squaramide based ion pair receptors possessing ferrocene as a signalling unit. Inorg. Chem. Front..

[74] Zaleskaya M., Karbarz M., Wilczek M., Dobrzycki Ł., Romański J. (2020). Cooperative Transport and Selective Extraction of Sulfates by a Squaramide-Based Ion Pair Receptor: A Case of Adaptable Selectivity. Inorg. Chem..

[75] Jagleniec D., Dobrzycki Ł., Karbarz M., Romański J. (2019). Ion-pair induced supramolecular assembly formation for elective extraction and sensing of potassium sulfate. Chem. Sci..

[76] Cabell L.A., Best M.D., Lavigne J.J., Schneider S.E., Perreault D.M., Monahan M.-K., Anslyn E.V. (2001). Metal triggered fluorescence sensing of citrate using a synthetic receptor. J. Chem. Soc. Perkin Trans..

[77] Capitán-Vallvey L.F., Arroyo-Guerrero E., Fernández-Ramos M.D., Santoyo-Gonzalez F. (2005). Disposable Receptor-Based Optical Sensor for Nitrate. Anal. Chem..

[78] (2019). APEX3 V2019.

[79] (2019). SAINT V8.40A.

[80] (2019). TWINABS V2012/1.

[81] Sheldrick G.M. (2015). SHELXT—Integrated Space-Group and Crystal-Structure Determination. Acta Crystallogr. Sect. A Found. Adv..

[82] Sheldrick G.M. (2015). Crystal Structure Refinement with SHELXL. Acta Crystallogr. Sect. C Struct. Chem..

[83] Cowley J.M., Wilson A.J.C. (1992). International Tables for Crystallography. Volume C: Mathematical, Physical and Chemical Tables.

[84] Macrae C.F., Sovago I., Cottrell S.J., Galek P.T.A., McCabe P., Pidcock E., Platings M., Shields G.P., Stevens J.S., Towler P.A. (2020). Wood Mercury 4.0: From visualization to analysis, design and prediction. J. Appl. Cryst..

