# Supplementary material for: Highly Efficient, Tripodal Ion-Pair Receptors for Switching Selectivity between Acetates and Sulfates Using Solid–Liquid and Liquid–Liquid Extractions
Source: Int J Mol Sci. 2020 Dec 12;21(24):9465. doi: 10.3390/ijms21249465 (PMC7764408; doi:10.3390/ijms21249465)
Supplement: Supplementary file 1 [file ijms-21-09465-s001.pdf]

Supporting Information

Highly efficient, tripodal ion-pair receptor for switching selectivity between acetates and sulfates using solid-liquid and liquid-liquid extractions

Marta Zaleskaya [1], Łukasz Dobrzycki [1] and Jan Romański [1,*]

[1] Faculty of Chemistry, University of Warsaw, Pasteura 1, PL 02-093 Warsaw, Poland

* Correspondence: E-mail: jarom@chem.uw.edu.pl

Table of Contents

1. **General information** **S1**
2. **NMR spectra** **S2**
3. **NMR titration experiments** **S6**
4. **UV-vis titration experiments** **S26**
5. **DOSY, ROESY and HSQC experiments** **S35**
6. **Extraction experiments** **S36**
7. **Crystal data** **S41**
8. **References** **S47**

1. General information

Unless specifically indicated, all other chemicals and reagents used in this study were purchased from commercial sources and used as received. If necessary purification of products was performed using column chromatography on silica gel (Merck Kieselgel 60, 230-400 mesh) with mixtures of chloroform/methanol. Thin-layer chromatography (TLC) was performed on silica gel plates (Merck Kieselgel 60 F254).

^{1}H and ^{13}C NMR spectra used in the characterization of products were recorded on Bruker 300 spectrometer using a residual protonated solvent as internal standard. DOSY experiments were conducted at 298 K on Varian VNMRS 600 MHz instruments with a residual solvent signal as an internal standard.

Mass spectra were measured on Quattro LC Micromass or Shimadzu LCMS-IT-TOF unit.

UV-vis analyses were performed using Thermo Spectronic Unicam UV500 Spectrophotometer.

High performance ion chromatography (HPIC) analyses were performed using a 930 Compact IC Flex apparatus – Metrohm AG.

2. NMR spectra

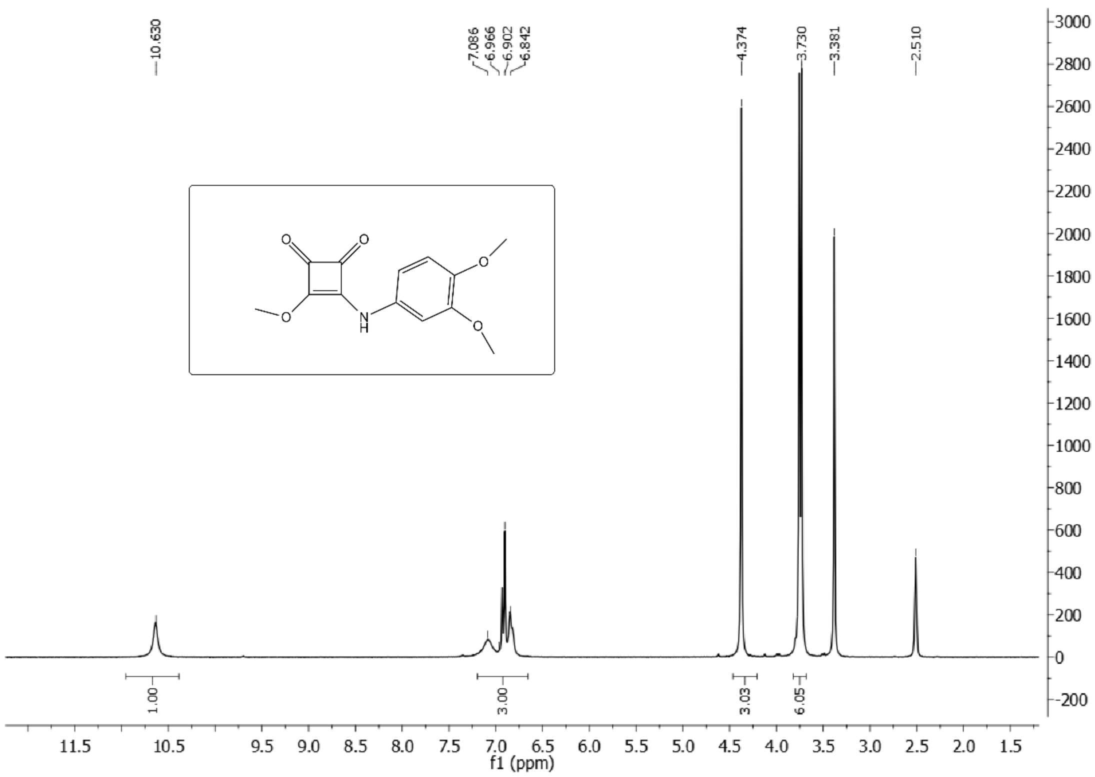

Fig. S1. ^{1}H NMR spectrum of module **5** in DMSO-d_6.

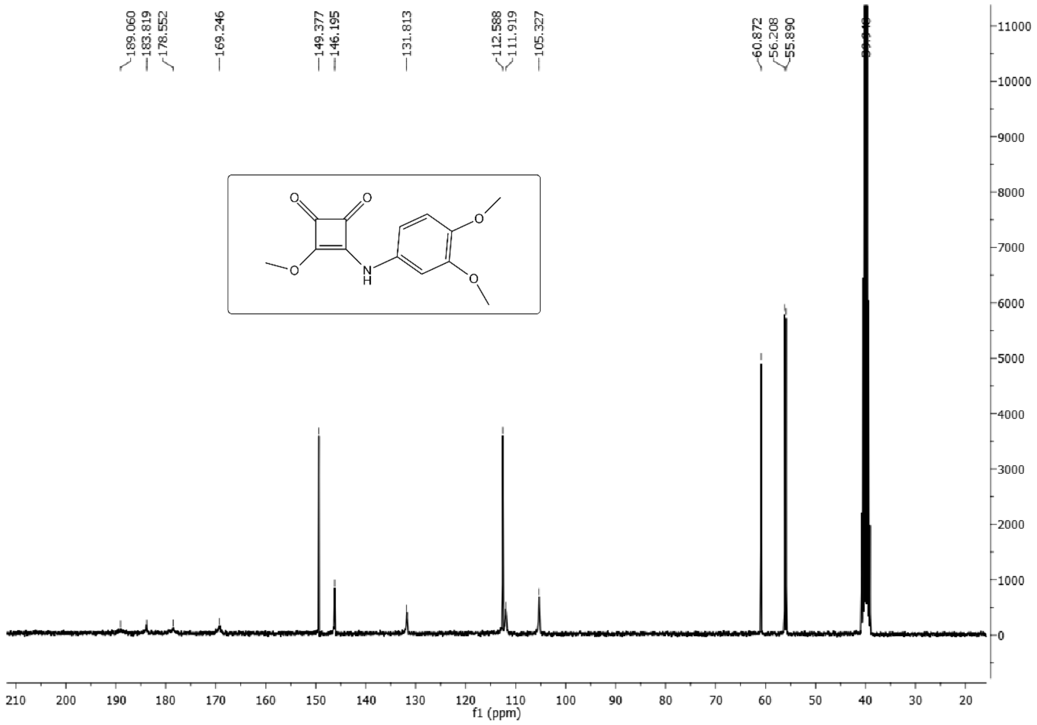

Fig. S2. ^{13}C NMR spectrum of module **5** in DMSO-d_6.

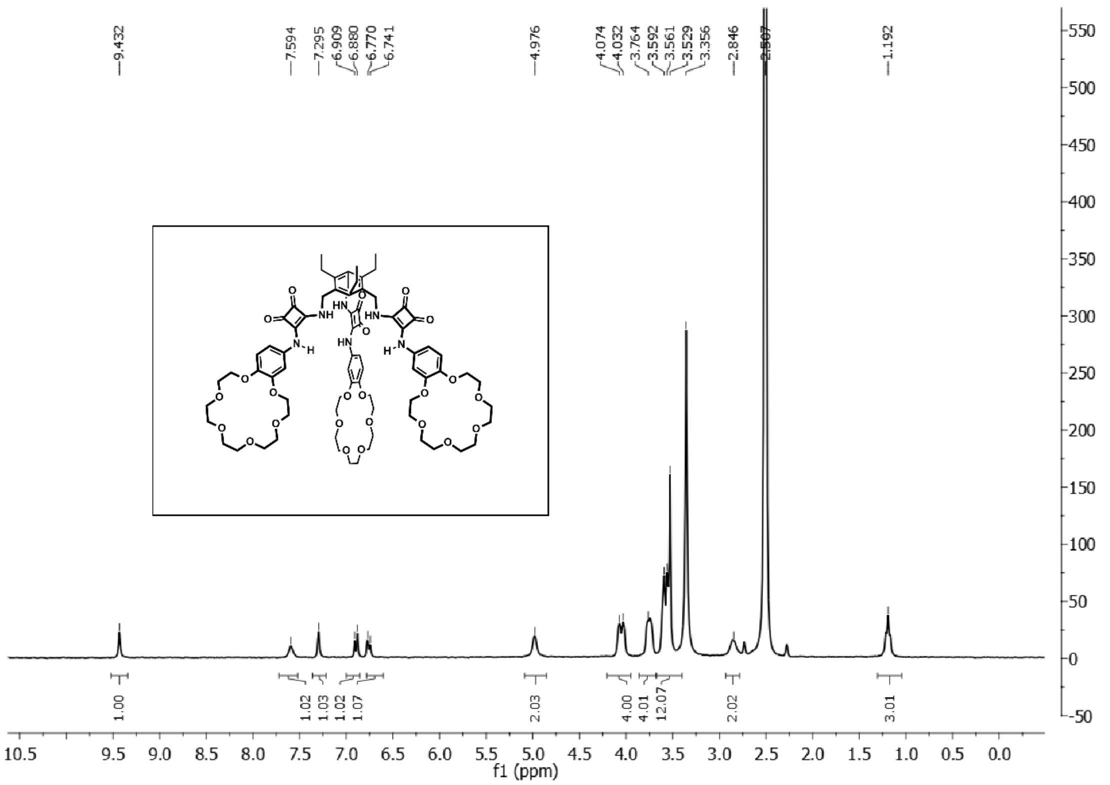

Fig. S3. ^{1}H NMR spectrum of Receptor **1** in DMSO-d_6.

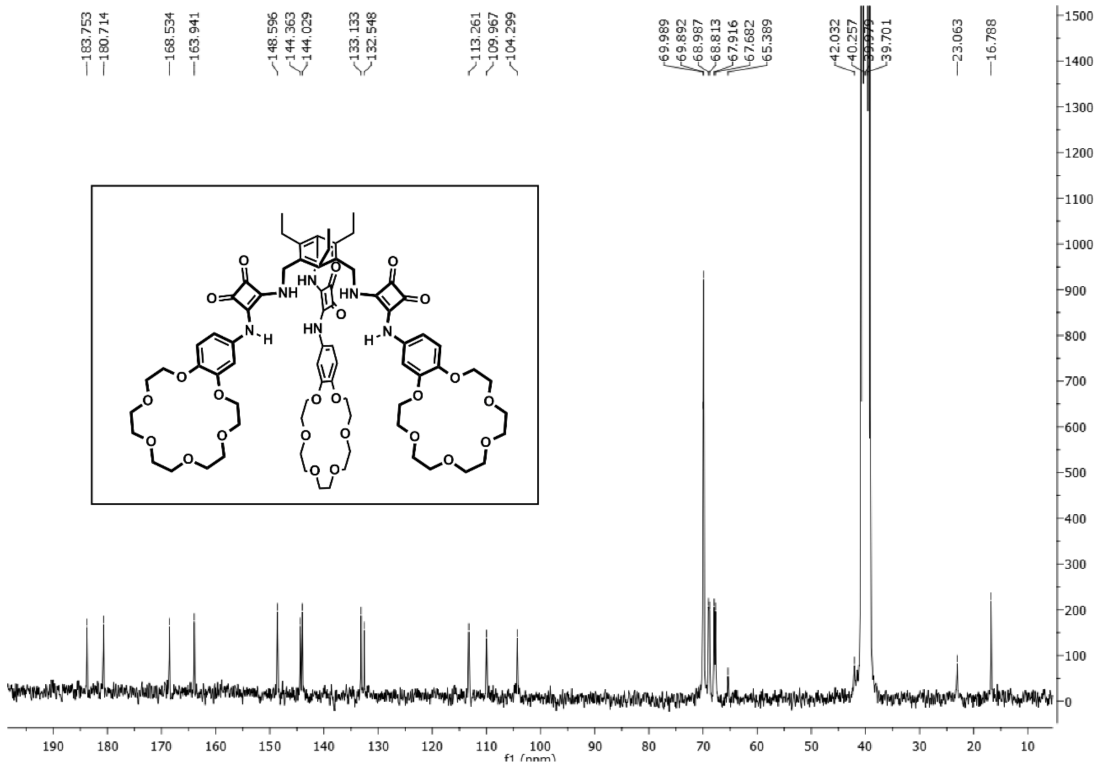

Fig. S4. ^{13}C NMR spectrum of Receptor **1** in DMSO-d_6.

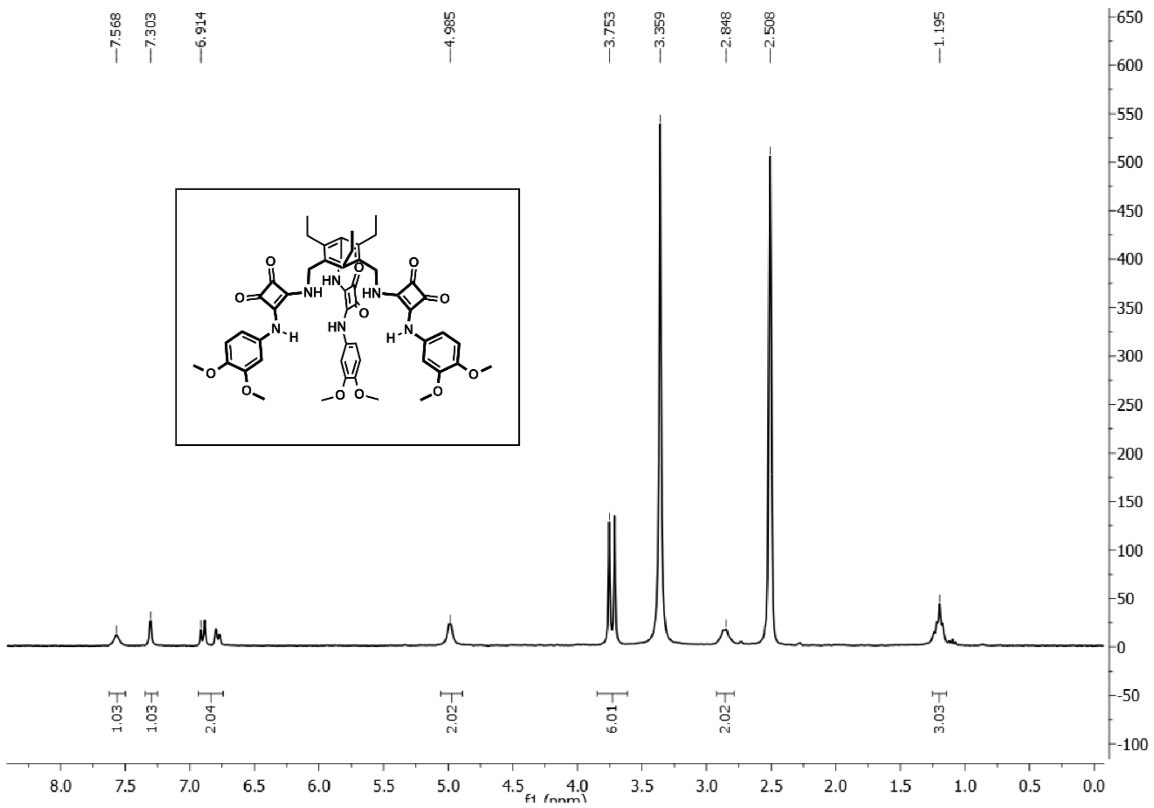

Fig. S5. ^{1}H NMR spectrum of Receptor **2** in DMSO-d_6.

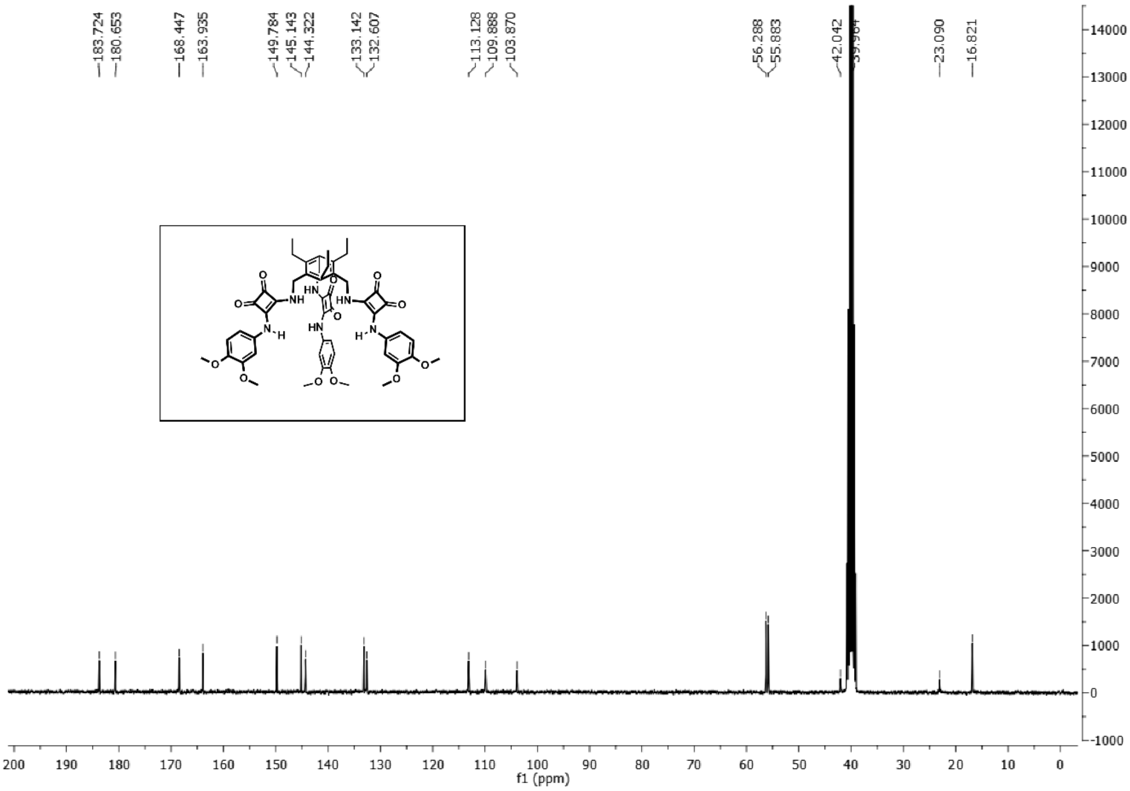

Fig. S6. ^{13}C NMR spectrum of Receptor **2** in DMSO-d_6.

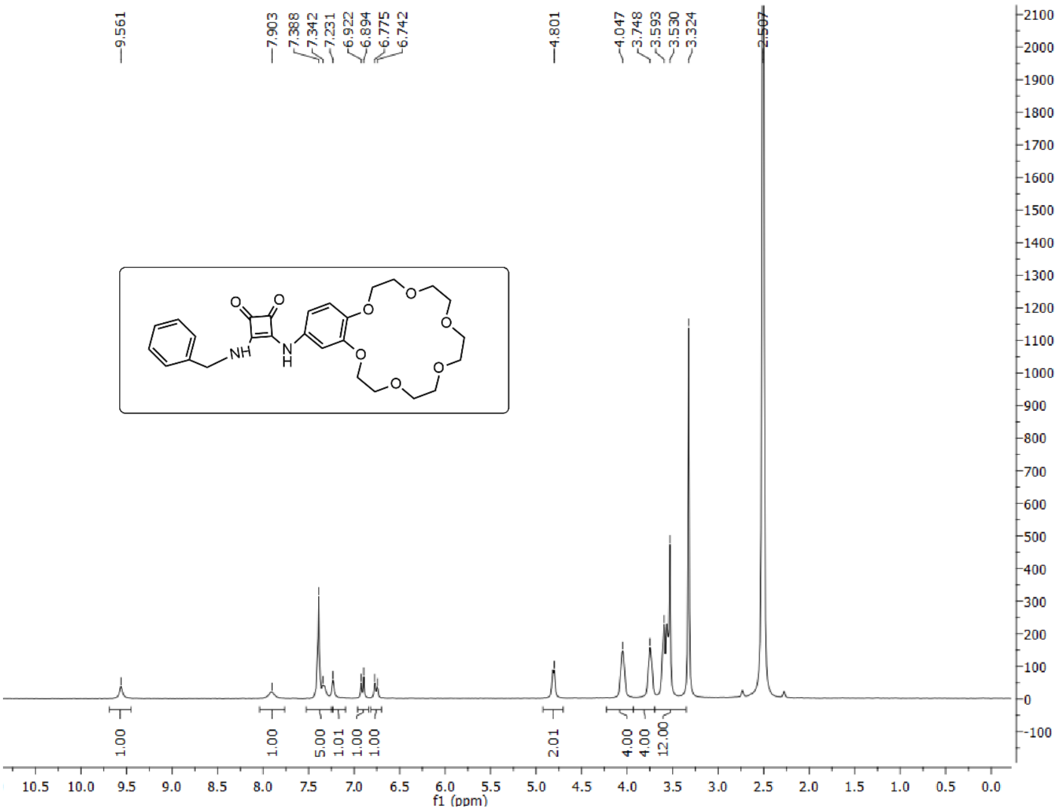

Fig. S7. ^{1}H NMR spectrum of Receptor **3** in DMSO-d$_6$.

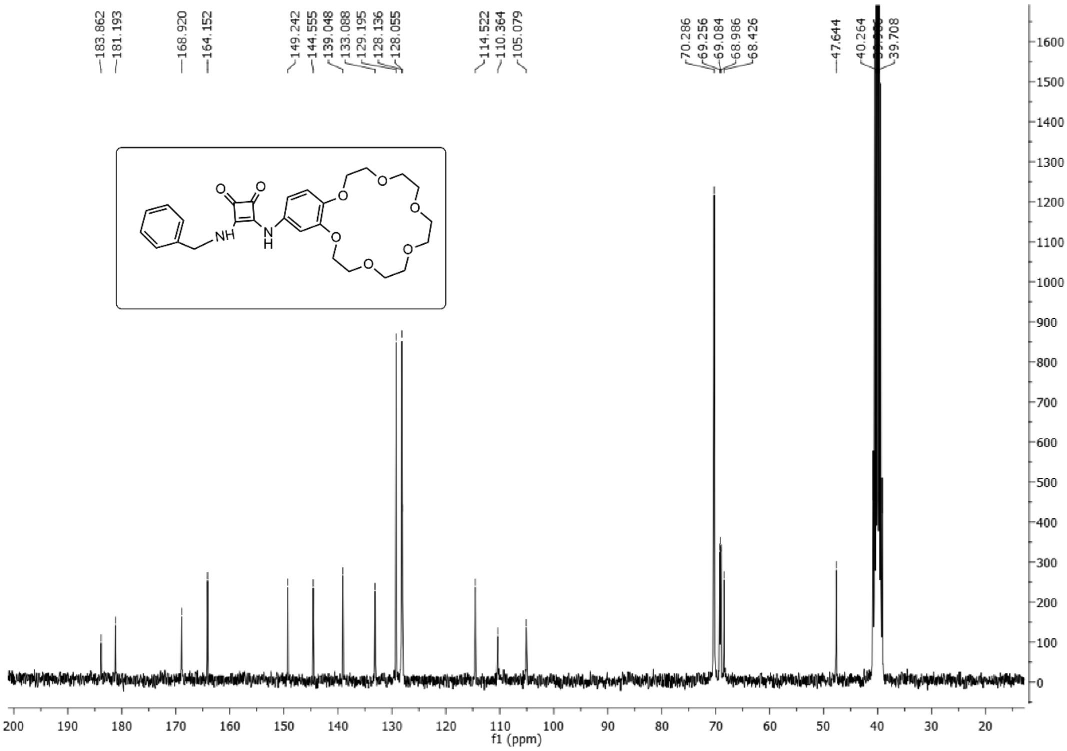

Fig. S8. ^{13}C NMR spectrum of Receptor **3** in DMSO-d$_6$.

3. NMR titration experiments

The ^{1}H NMR titration was performed on a Bruker 300 spectrometer, at 298K in DMSO-d$_6$. In each case, a 500 μL of freshly prepared 1.7 mM solution of receptor **1** (1.4 mM of receptor **2**; 2.0 mM of receptor **3**) was added to a 5 mm NMR tube. In the case of ion pair titration receptor was firstly pretreated with one or three equivalent of $NaClO_4$ or KPF_6 . Then small aliquots of solution of TBAX, containing receptor at constant concentration, were added and a spectrum was acquired after each addition. The resulting titration data were analyzed using BindFit (v0.5) package, available online at http://supramolecular.org.

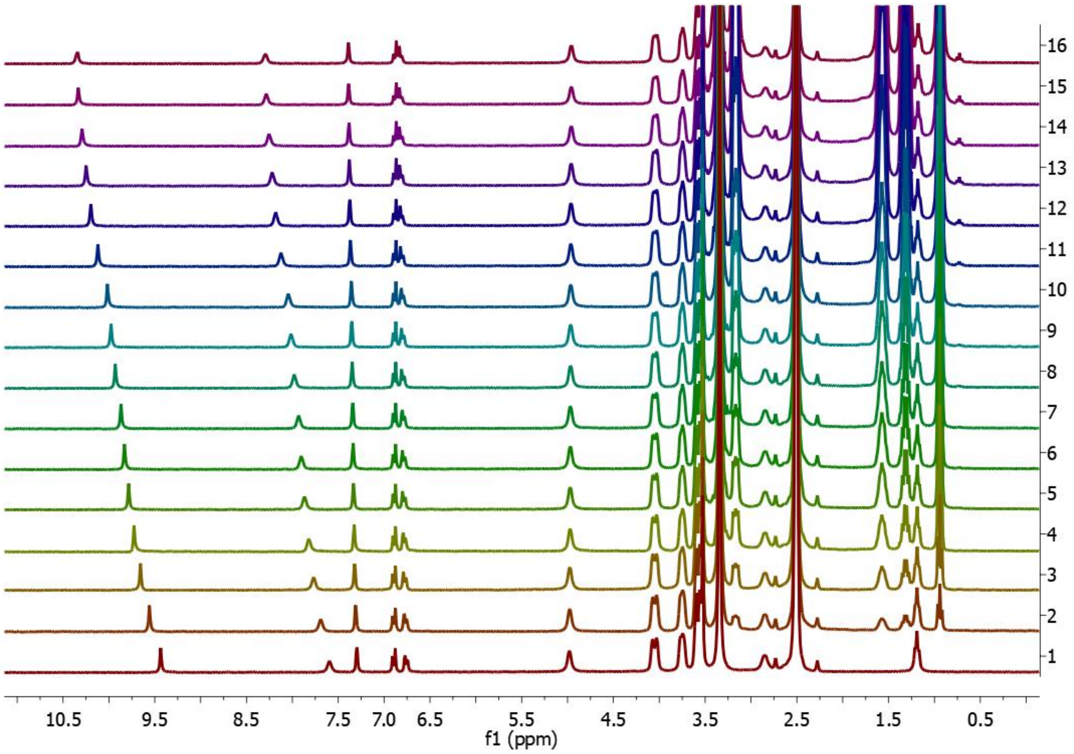

Fig. S9. ^{1}H NMR spectra recorded upon titration of receptor **1** in DMSO-d$_6$ with TBACl.

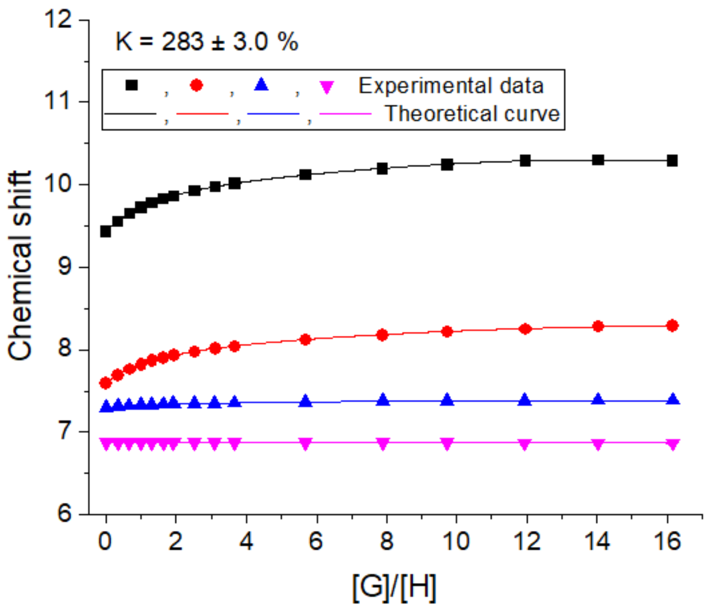

Fig. S10. ^{1}H NMR titration binding isotherms of receptor **1** in DMSO-d_6 upon addition of increasing amounts of TBACl.

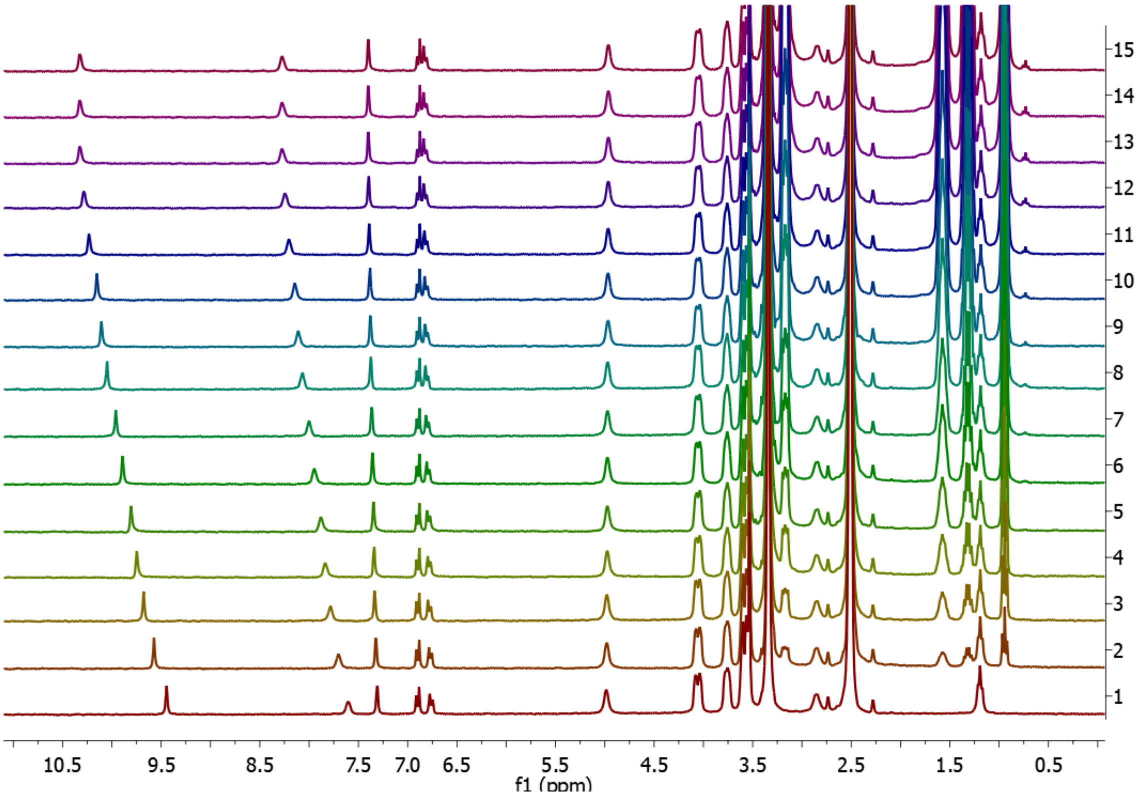

Fig. S11. ^{1}H NMR spectra recorded upon titration of receptor **1** in DMSO-d_6 with TBACl in the presence of 3 eq. $NaClO_4$.

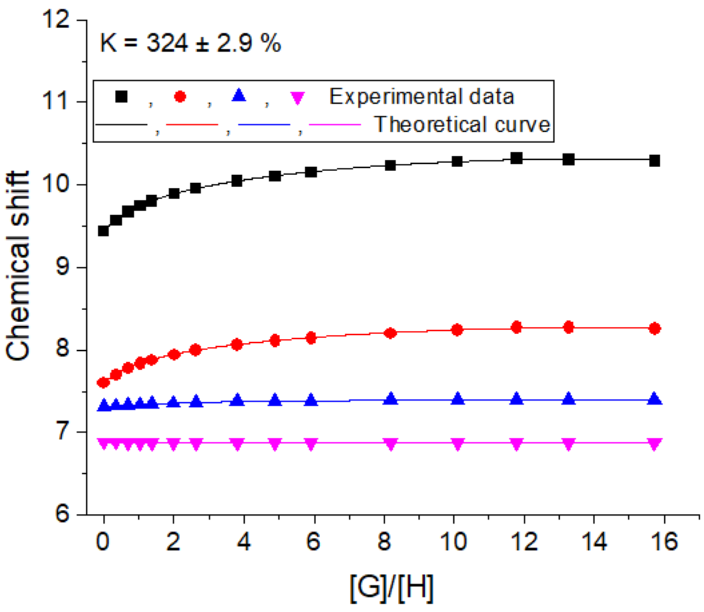

Fig. S12. ^{1}HNMR titration binding isotherms of receptor **1** in DMSO-d$_6$ upon addition of increasing amounts of TBACl in the presence of 3 eq. $NaClO_4$.

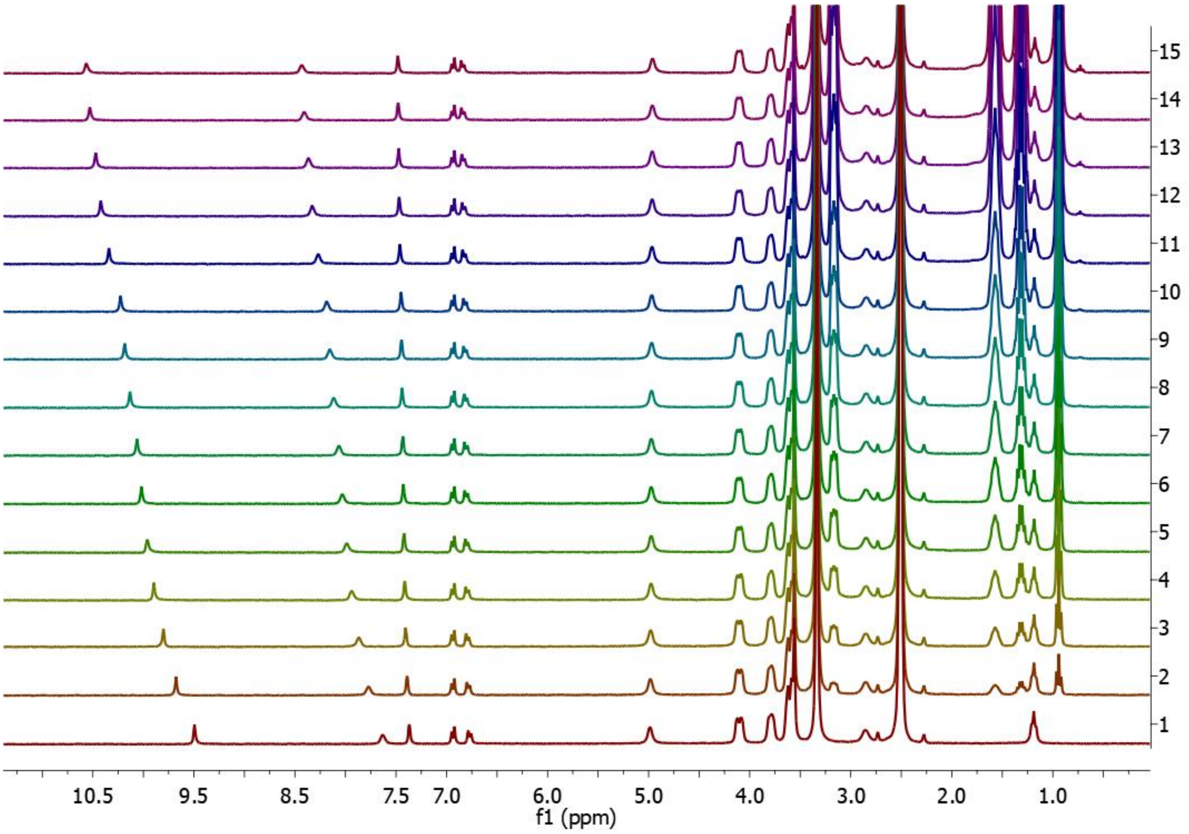

Fig. S13. ^{1}H NMR spectra recorded upon titration of receptor **1** in DMSO-d$_6$ with TBACl in the presence of 3 eq. KPF_6.

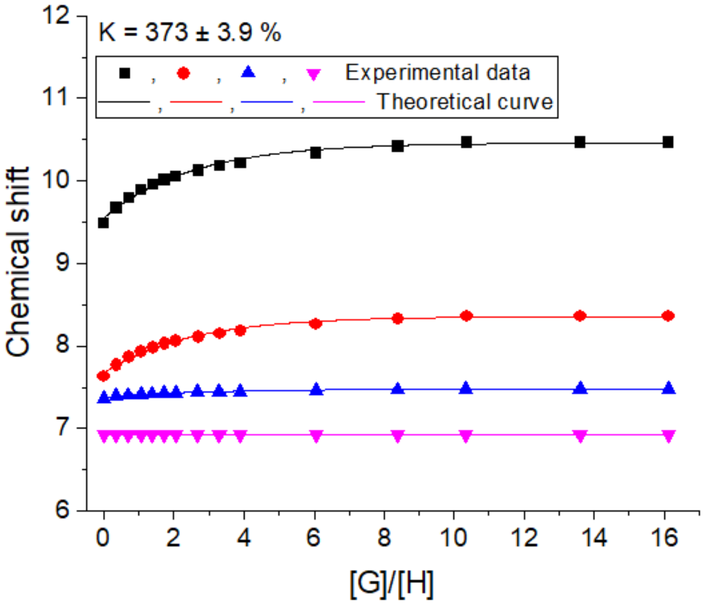

Fig. S14. ^{1}HNMR titration binding isotherms of receptor **1** in DMSO-d_6 upon addition of increasing amounts of TBACl in the presence of 3 eq. KPF_6.

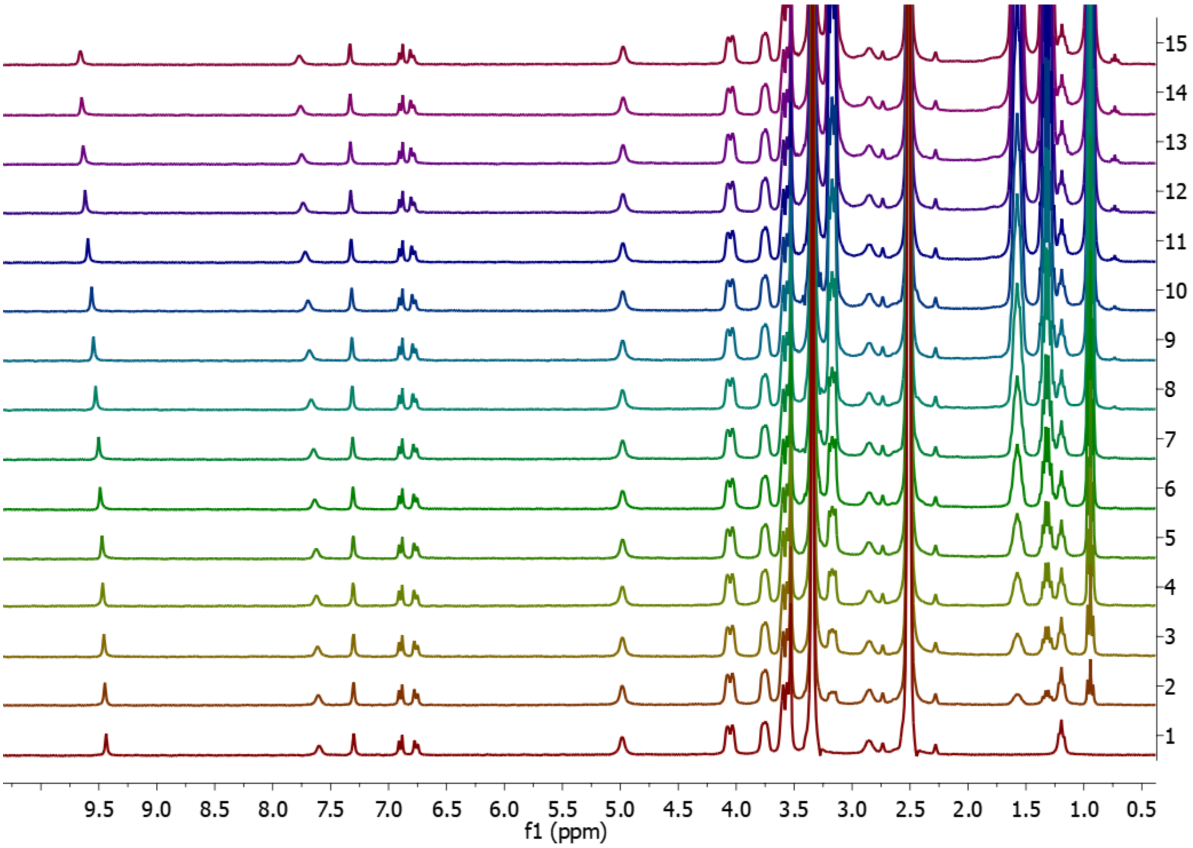

Fig. S15. ^{1}H NMR spectra recorded upon titration of receptor **1** in DMSO-d_6 with TBABr.

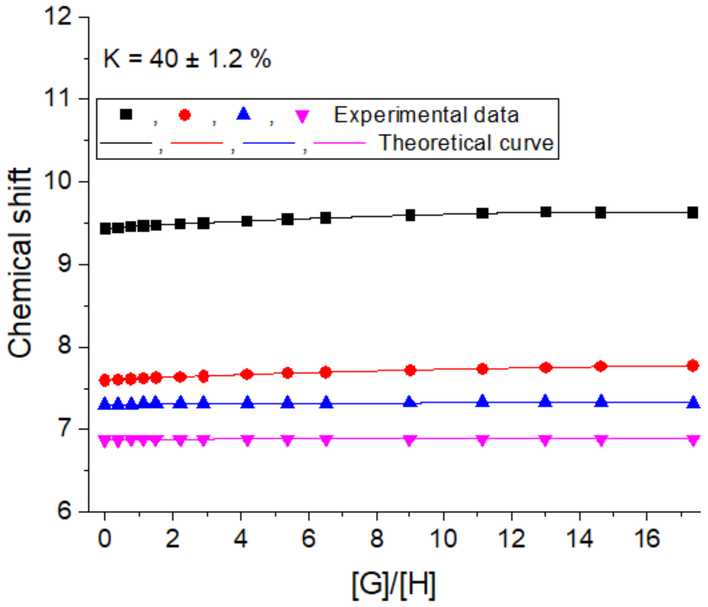

Fig. S16. ^{1}H NMR titration binding isotherms of receptor **1** in DMSO-d$_6$ upon addition of increasing amounts of TBABr.

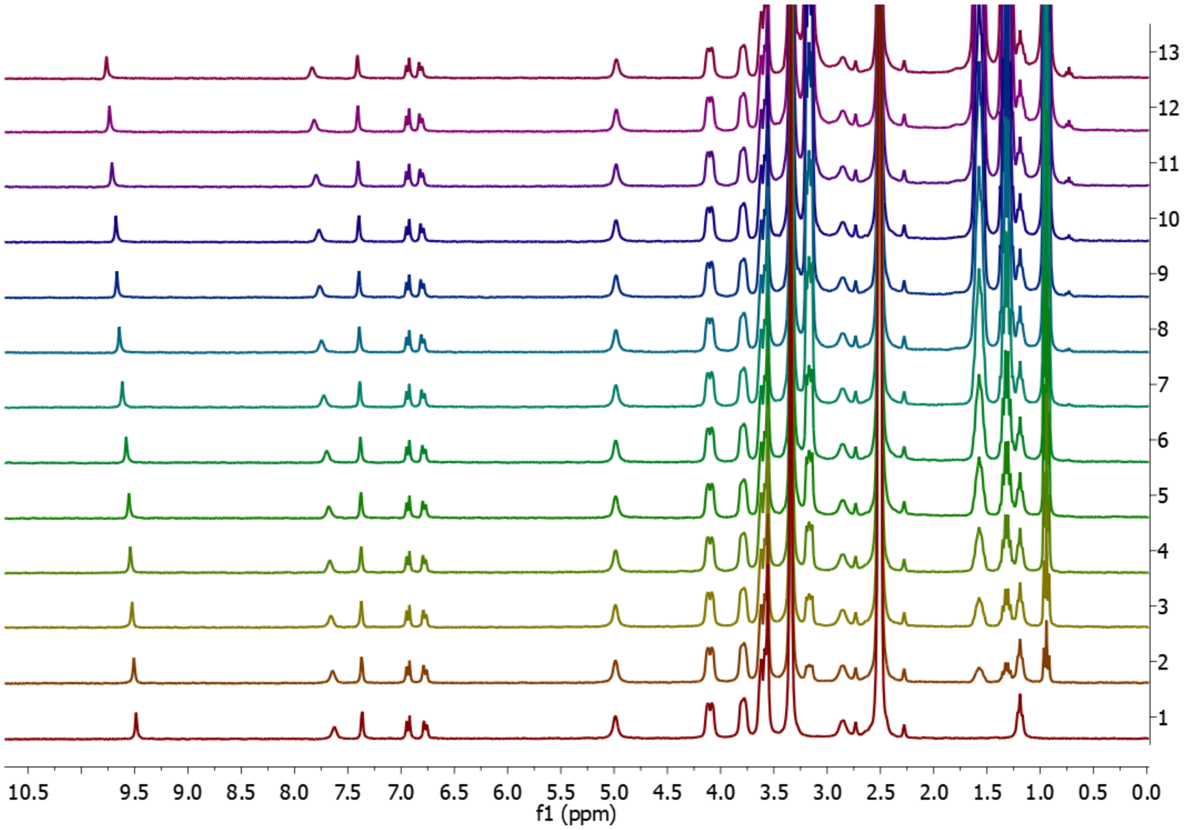

Fig. S17. ^{1}H NMR spectra recorded upon titration of receptor **1** in DMSO-d$_6$ with TBABr in the presence of 3 eq. KPF_6.

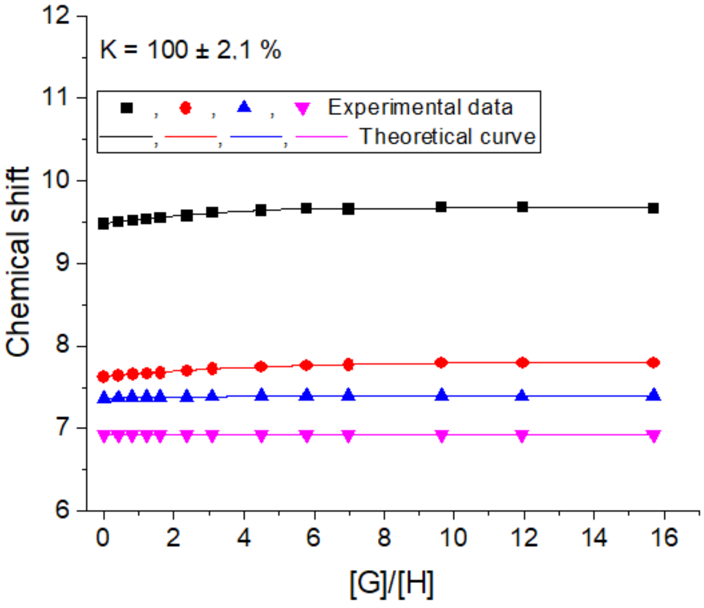

Fig. S18. ^{1}HNMR titration binding isotherms of receptor **1** in DMSO-d_6 upon addition of increasing amounts of TBABr in the presence of 3 eq. KPF_6.

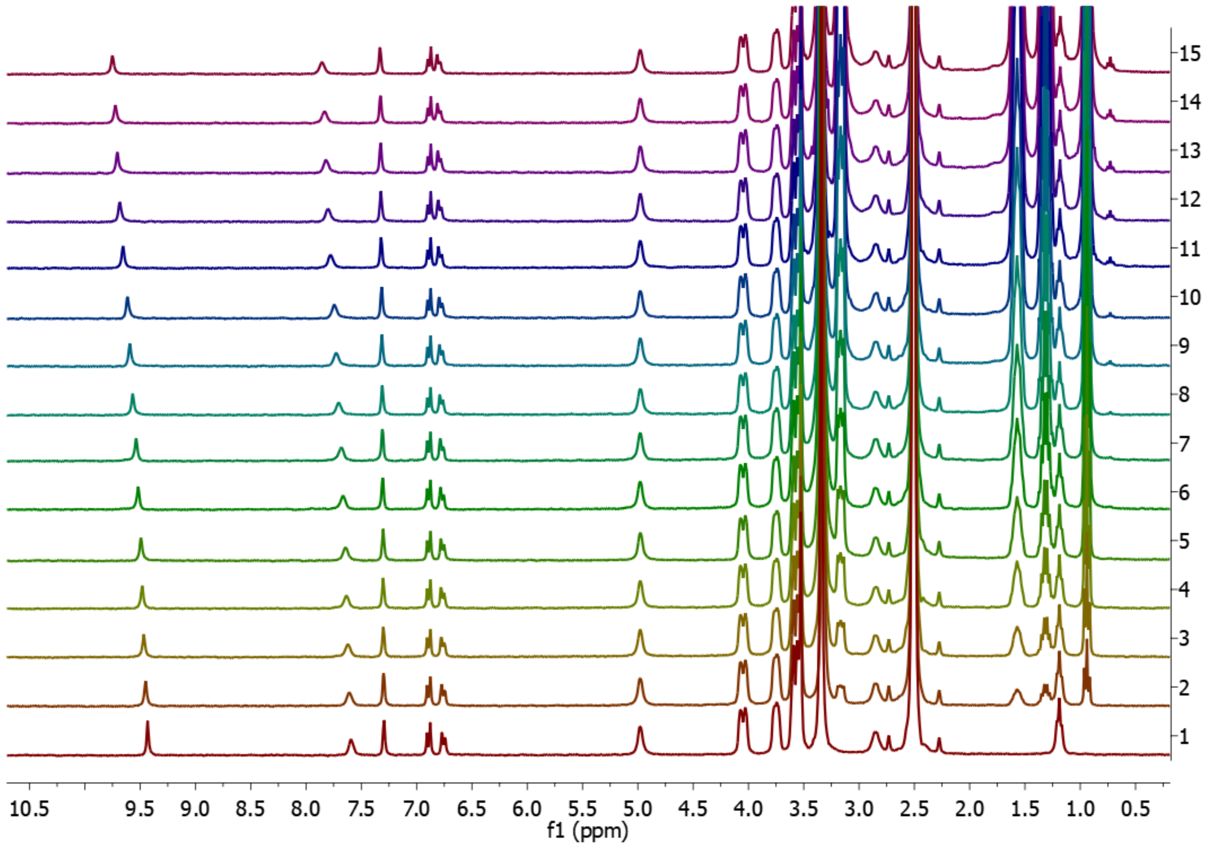

Fig. S19. ^{1}H NMR spectra recorded upon titration of receptor **1** in DMSO-d_6 with $TBANO_2$.

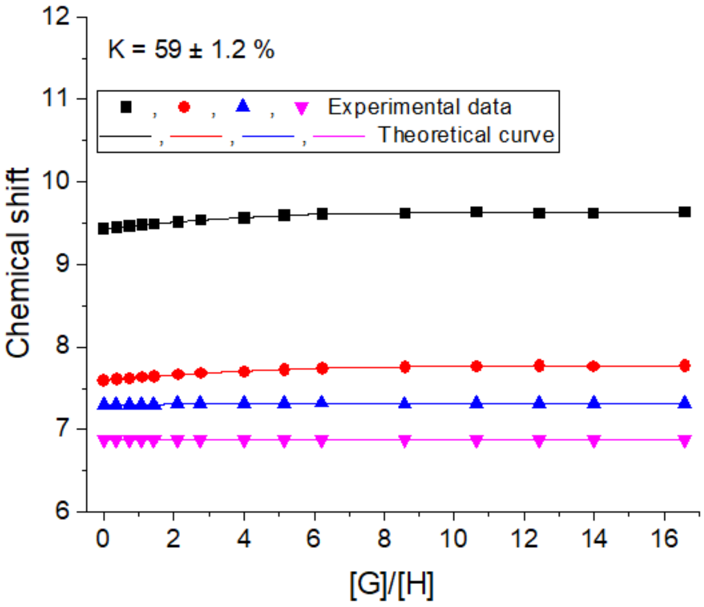

Fig. S20. ^{1}H NMR titration binding isotherms of receptor **1** in DMSO-d_6 upon addition of increasing amounts of $TBANO_2$.

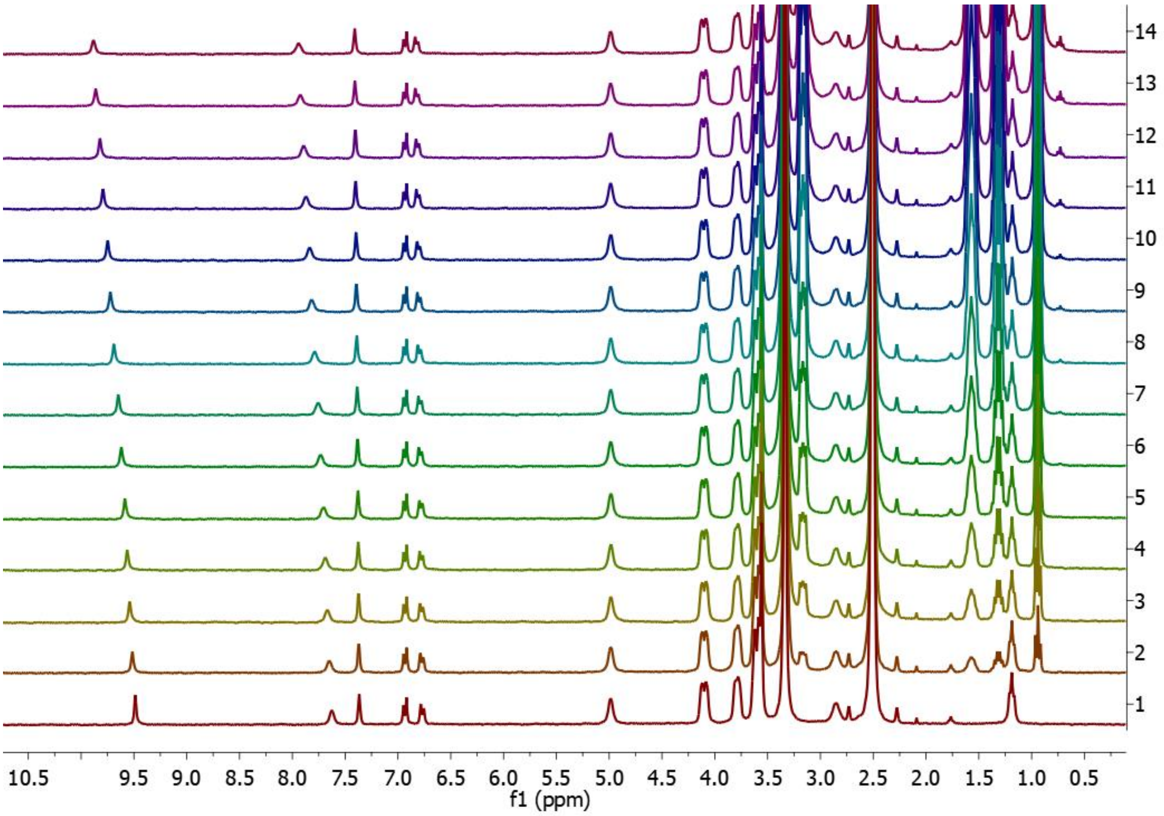

Fig. S21. ^{1}H NMR spectra recorded upon titration of receptor **1** in DMSO-d_6 with $TBANO_2$ in the presence of 3 eq. KPF_6.

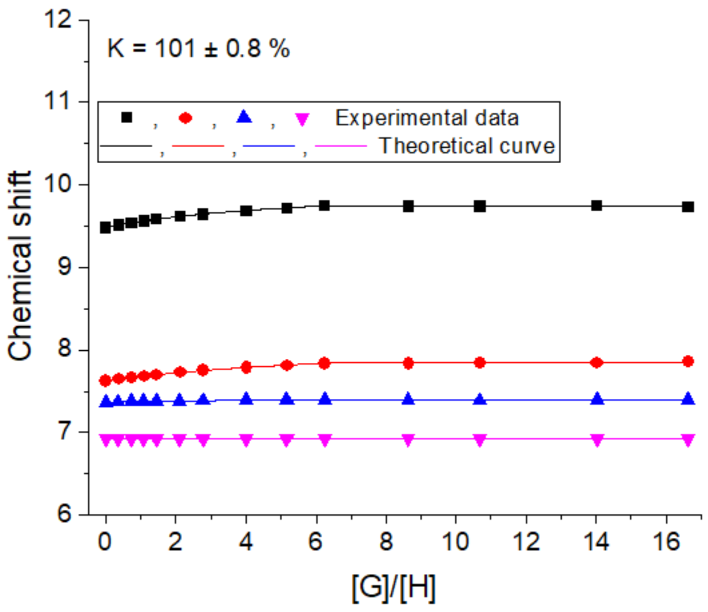

Fig. S22. ^{1}HNMR titration binding isotherms of receptor **1** in DMSO-d_6 upon addition of increasing amounts of $TBANO_2$ in the presence of 3 eq. KPF_6.

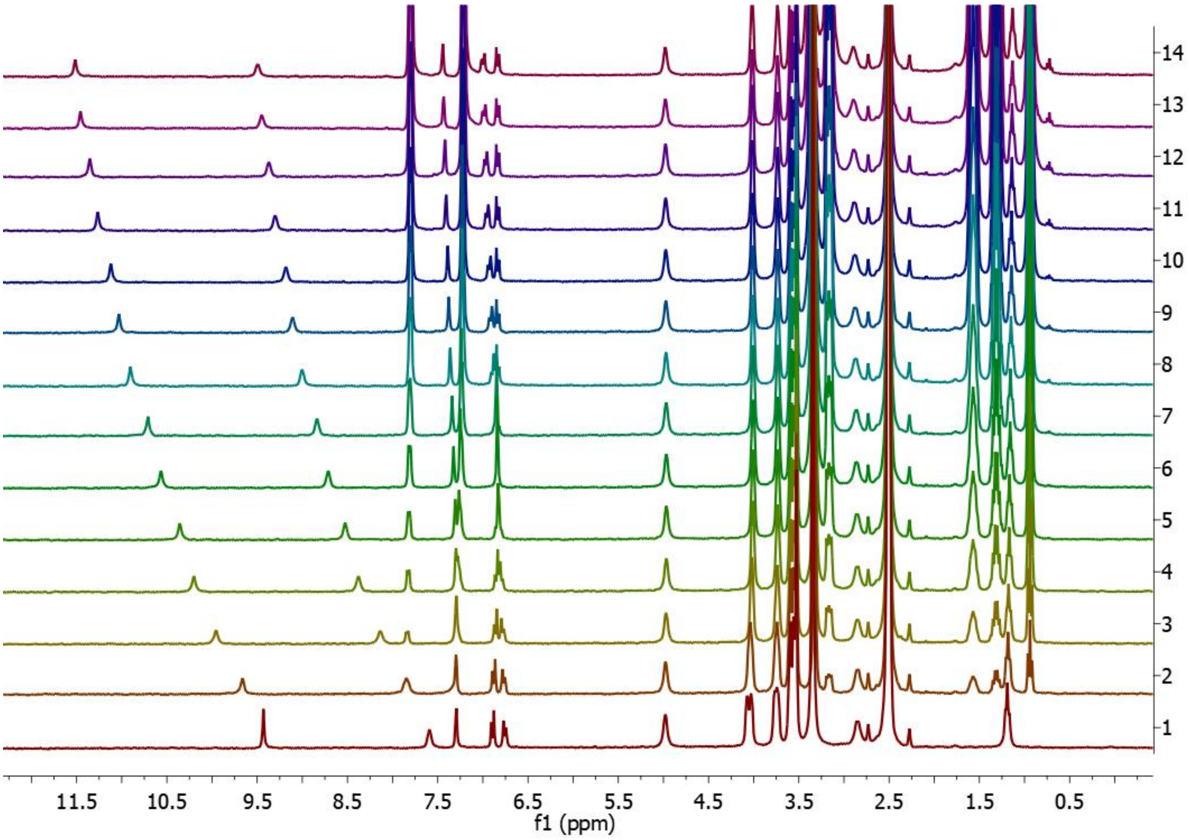

Fig. S23. ^{1}H NMR spectra recorded upon titration of receptor **1** in DMSO-d_6 with TBAPhCOO.

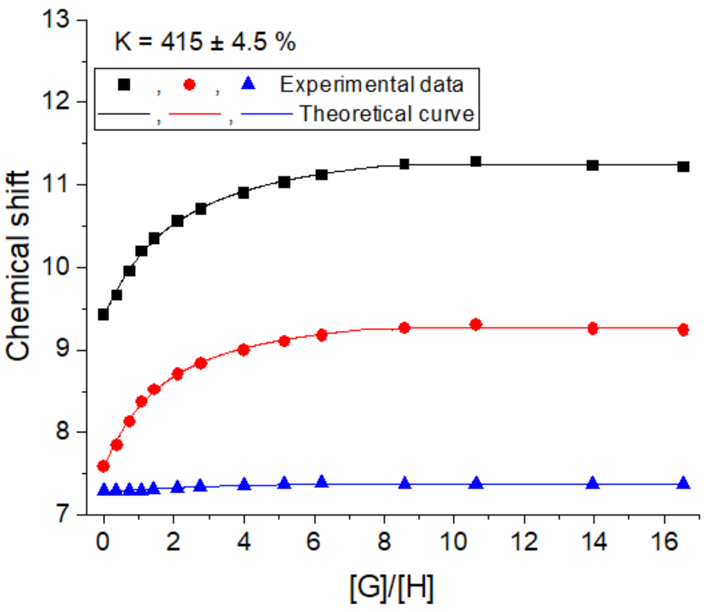

Fig. S24. ^{1}H NMR titration binding isotherms of receptor **1** in DMSO-d$_6$ upon addition of increasing amounts of TBAPhCOO.

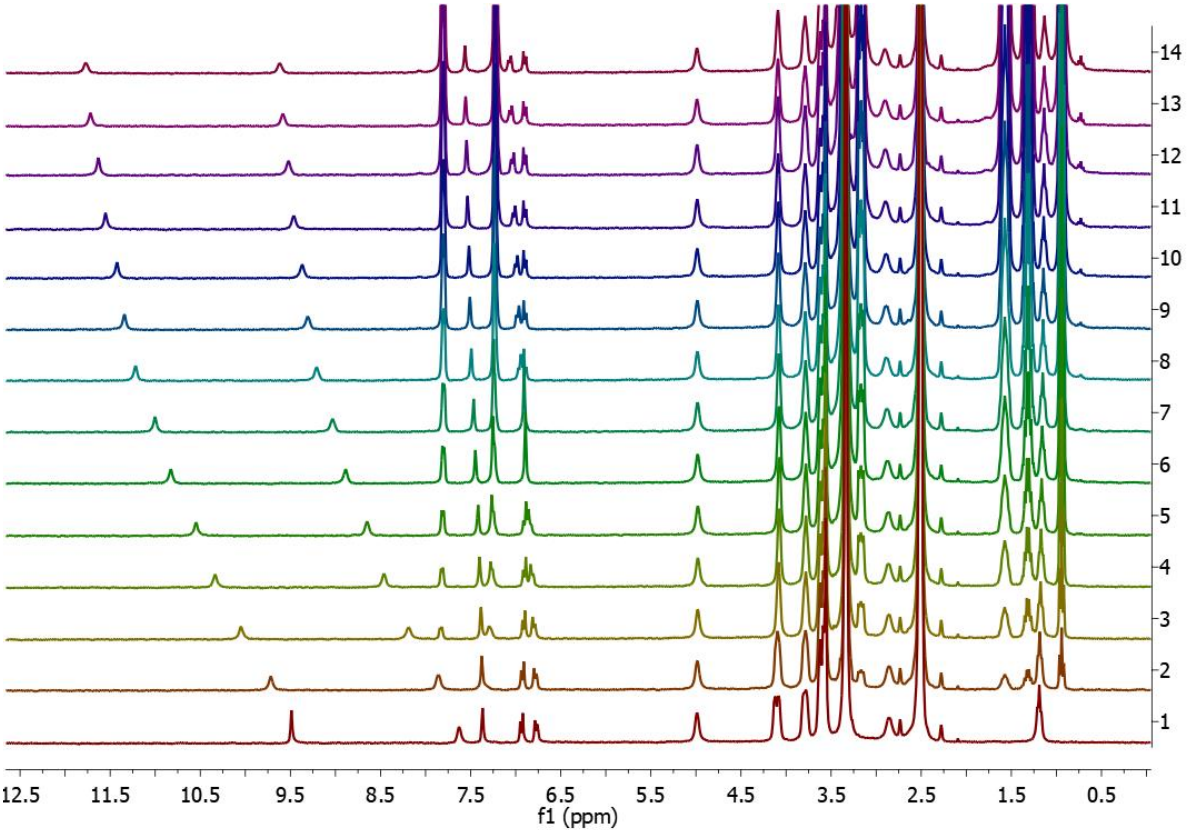

Fig. S25. ^{1}H NMR spectra recorded upon titration of receptor **1** in DMSO-d$_6$ with TBAPhCOO in the presence of 3 eq. KPF$_6$.

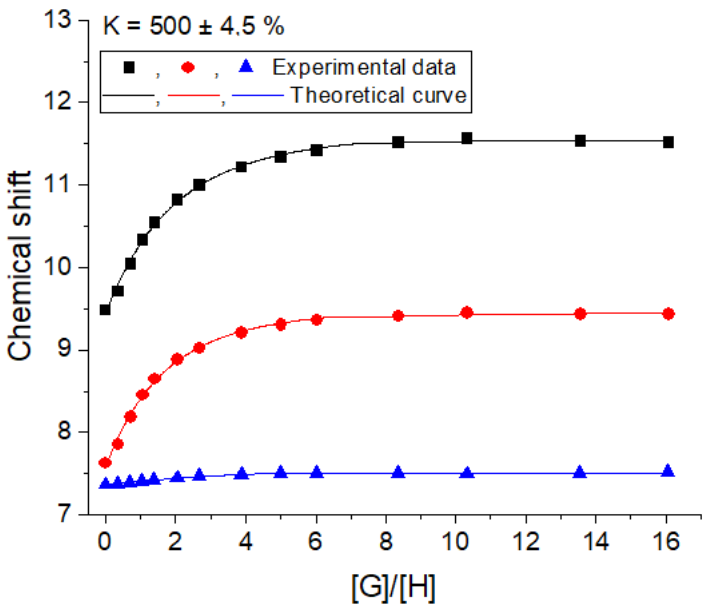

Fig. S26. ^{1}HNMR titration binding isotherms of receptor **1** in DMSO-d$_6$ upon addition of increasing amounts of TBAPhCOO in the presence of 3 eq. KPF_6.

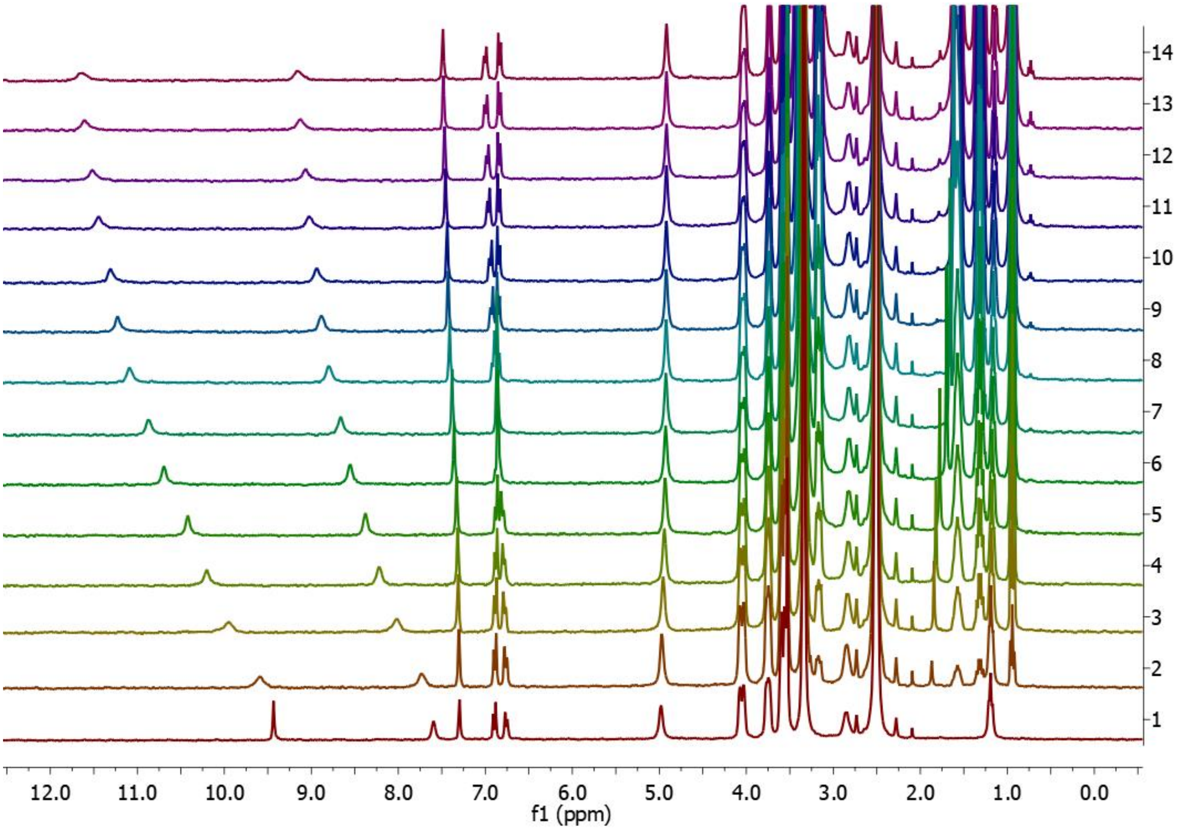

Fig. S27. ^{1}H NMR spectra recorded upon titration of receptor **1** in DMSO-d$_6$ with TBACH_3COO.

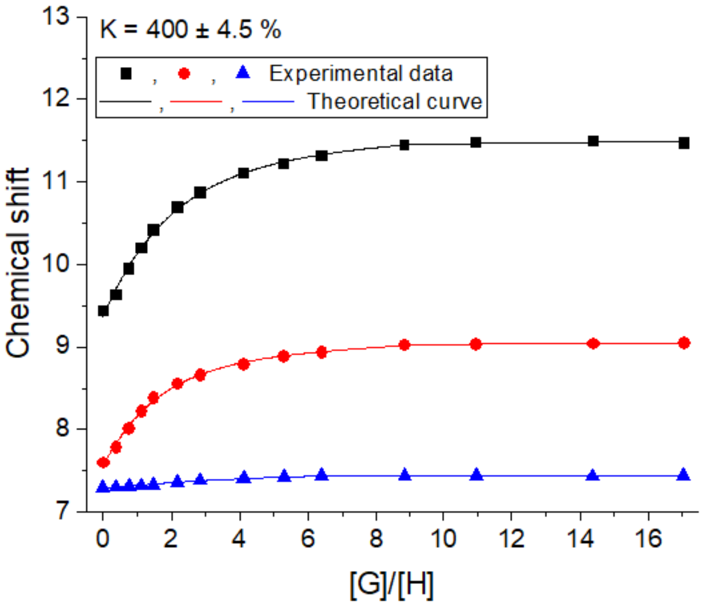

Fig. S28. ^{1}H NMR titration binding isotherms of receptor **1** in DMSO-d_6 upon addition of increasing amounts of TBACH_3COO.

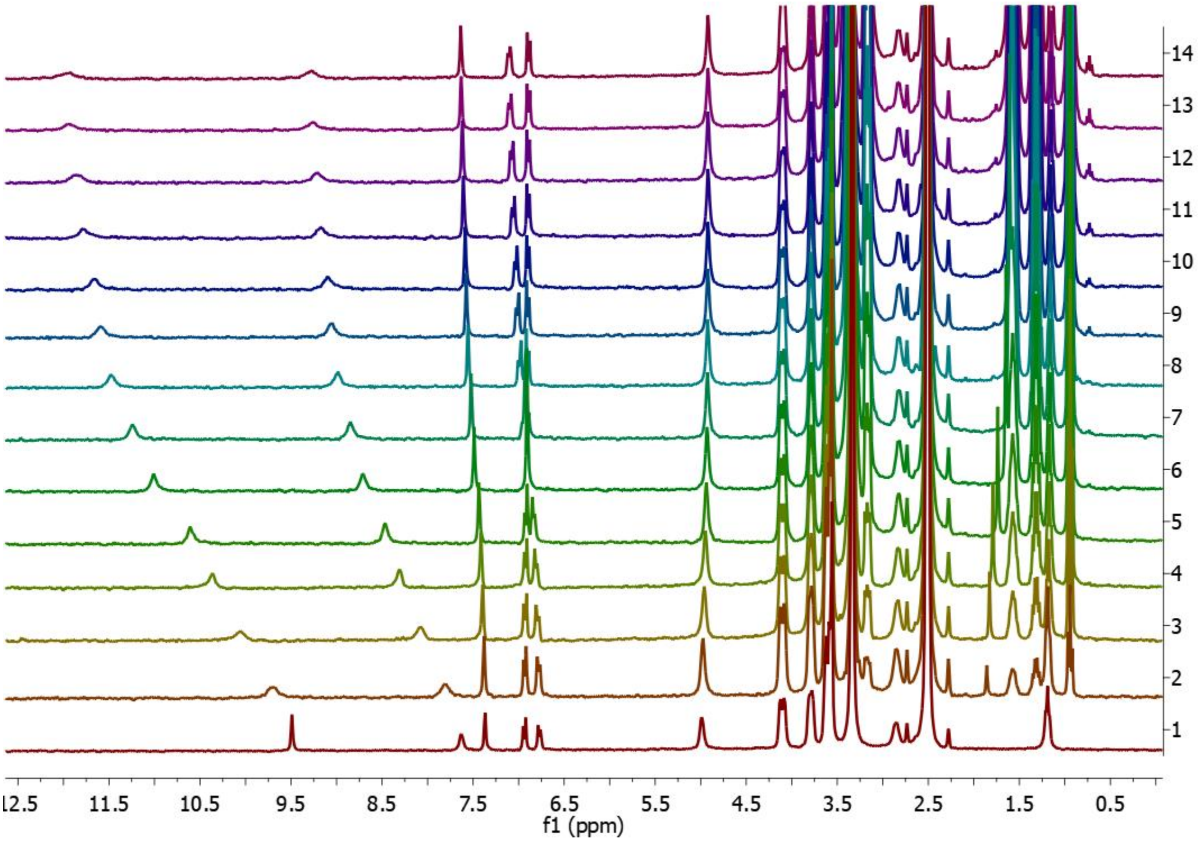

Fig. S29. ^{1}H NMR spectra recorded upon titration of receptor **1** in DMSO-d_6 with TBACH_3COO in the presence of 3 eq. KPF_6.

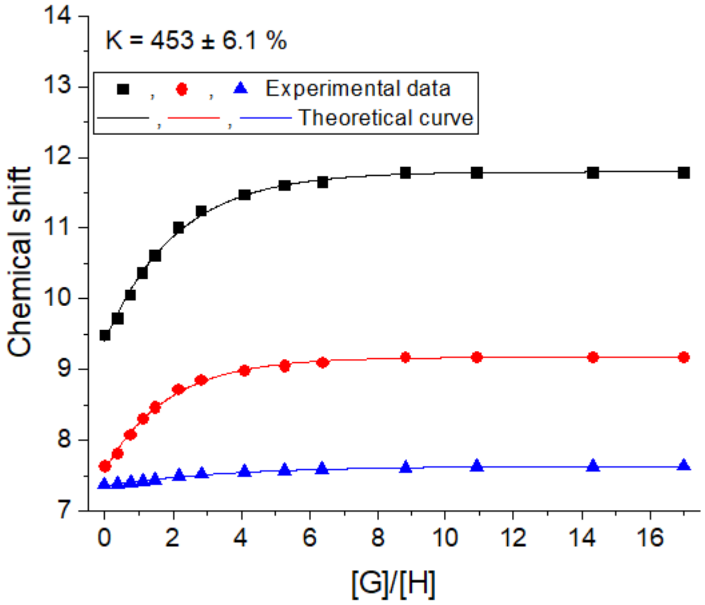

Fig. S30. ^{1}HNMR titration binding isotherms of receptor **1** in DMSO-d$_6$ upon addition of increasing amounts of TBACH$_3$COO in the presence of 3 eq. KPF$_6$.

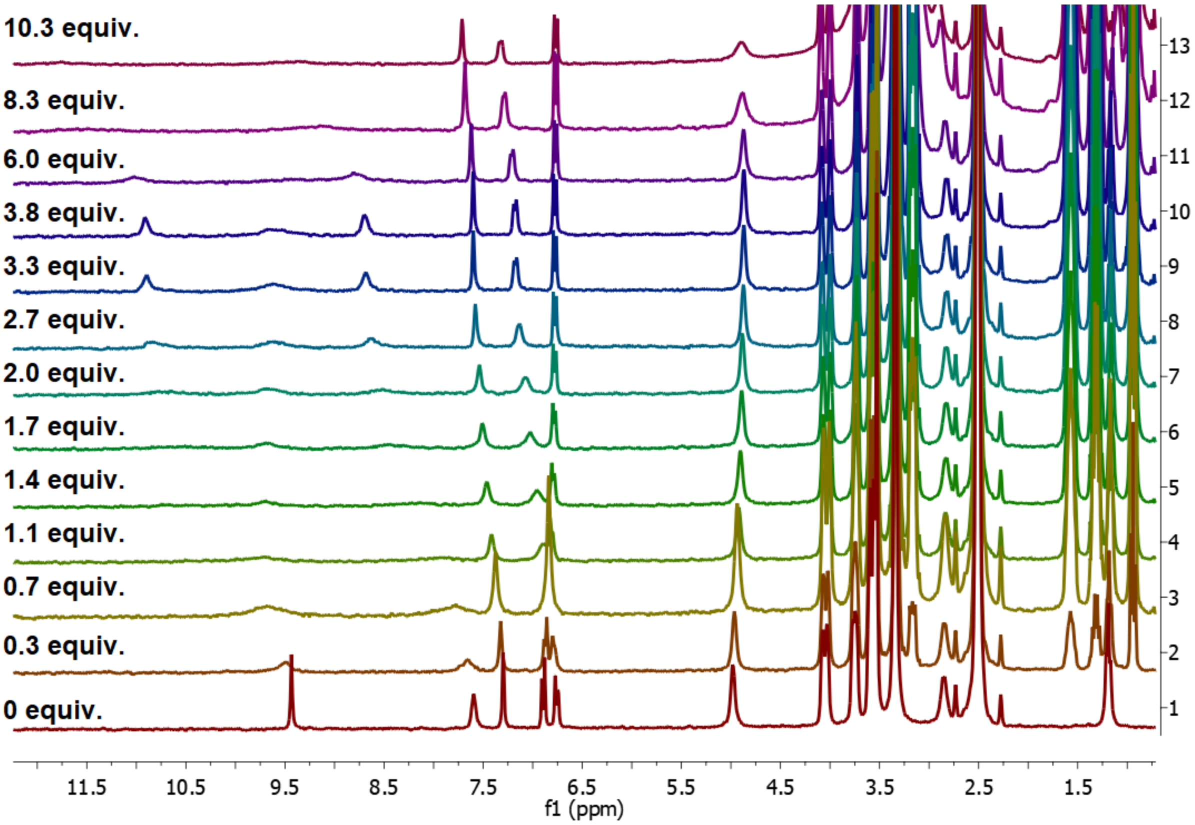

Fig. S31. ^{1}H NMR spectra recorded upon titration of receptor **1** in DMSO-d$_6$ with TBA$_2$SO$_4$.

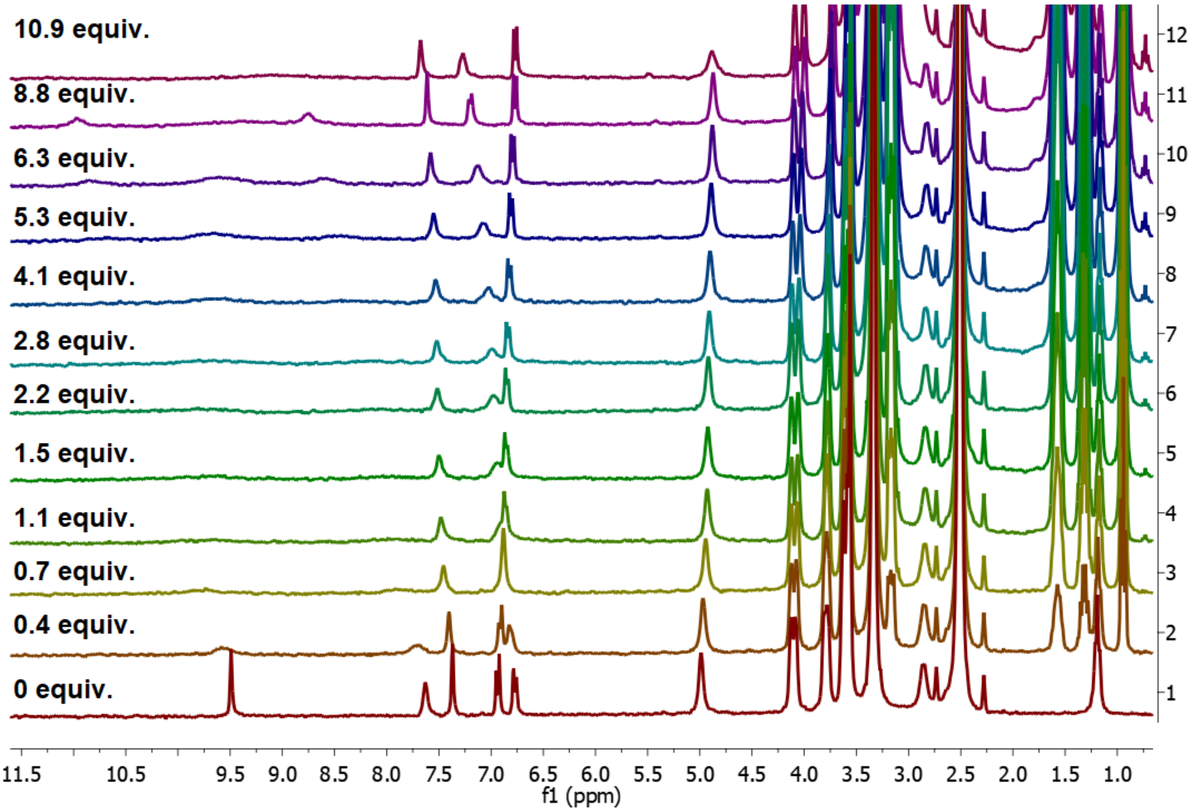

Fig. S32. ^{1}H NMR spectra recorded upon titration of receptor **1** in DMSO-d_6 with TBA_2SO_4 in the presence of 3 eq. KPF_6.

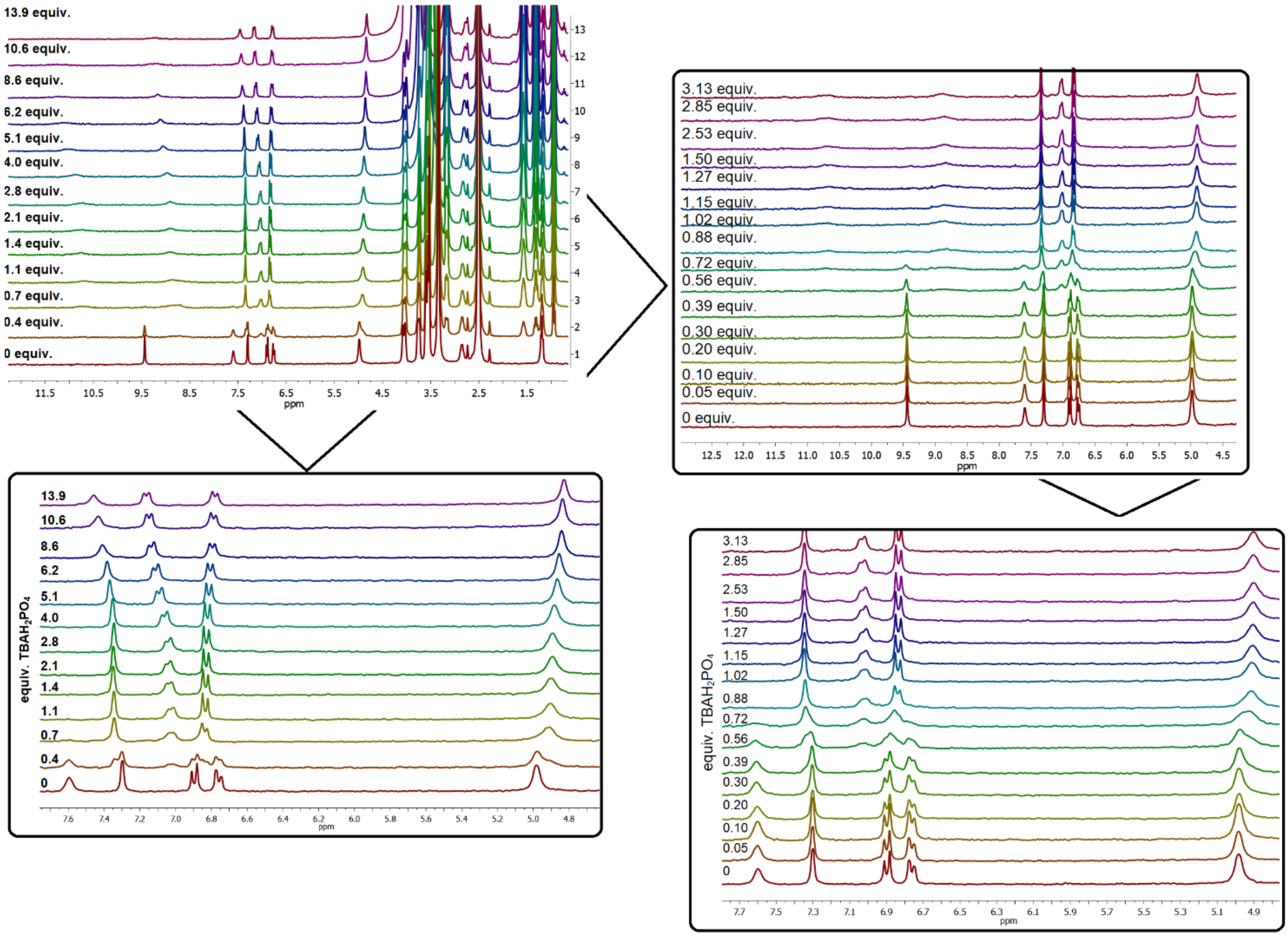

Fig. S33. ^{1}H NMR spectra recorded upon titration of receptor **1** in DMSO-d_6 with $TBAH_2PO_4$. Left figure – titration performed in the range from 0.0 equiv. to 13.9 equiv., right figure - titration performed in the range from 0.0 equiv. to 3.13 equiv. (Signals corresponding to amide and phenyl protons).

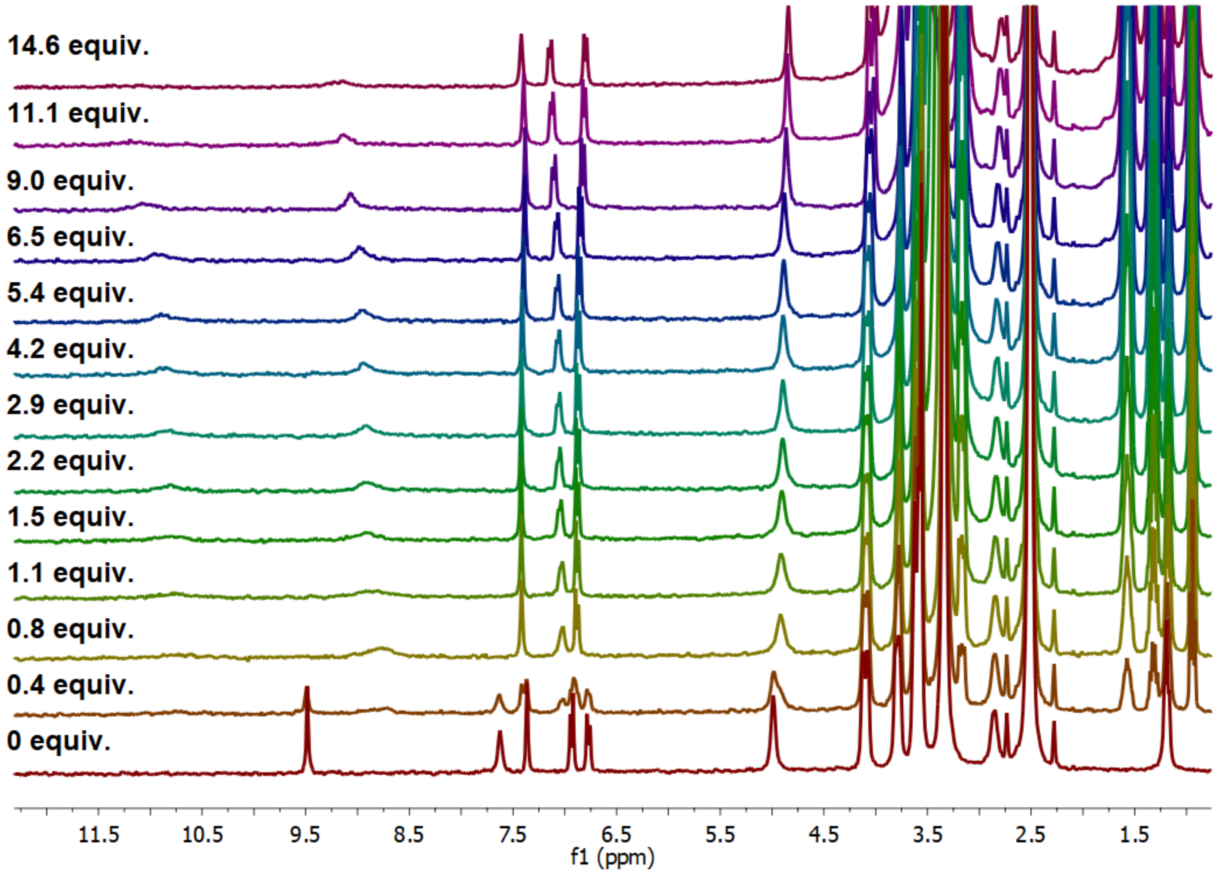

Fig. S34. ^{1}H NMR spectra recorded upon titration of receptor **1** in DMSO-d$_6$ with TBAH_2PO_4 in the presence of 3 eq. KPF_6.

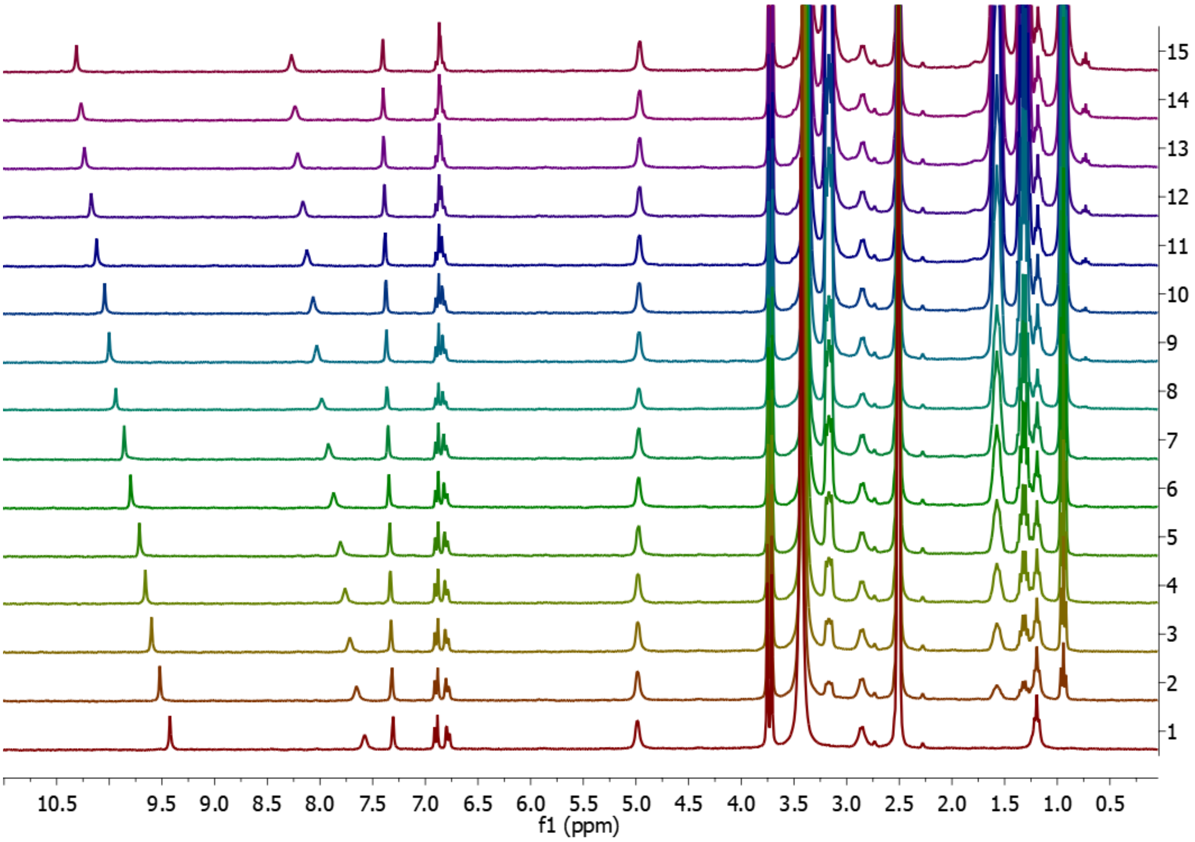

Fig. S35. ^{1}H NMR spectra recorded upon titration of receptor **2** in DMSO-d$_6$ with TBACl.

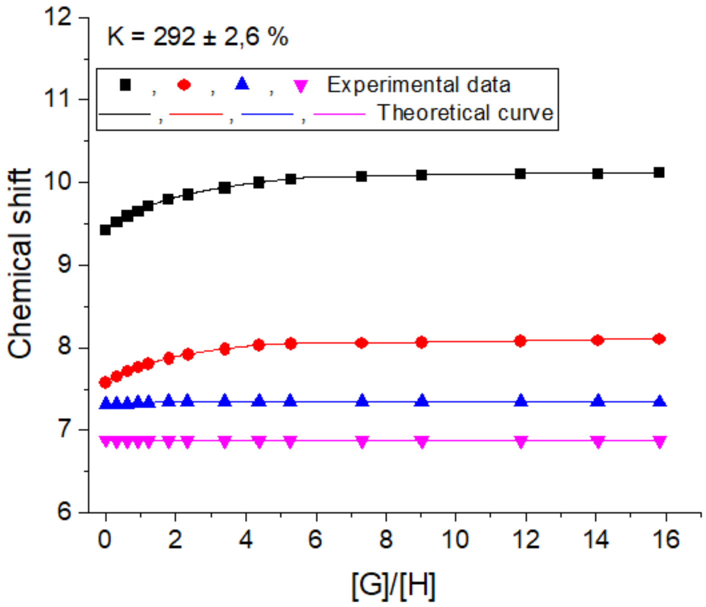

Fig. S36. ^{1}H NMR titration binding isotherms of receptor **2** in DMSO-d$_6$ upon addition of increasing amounts of TBACl.

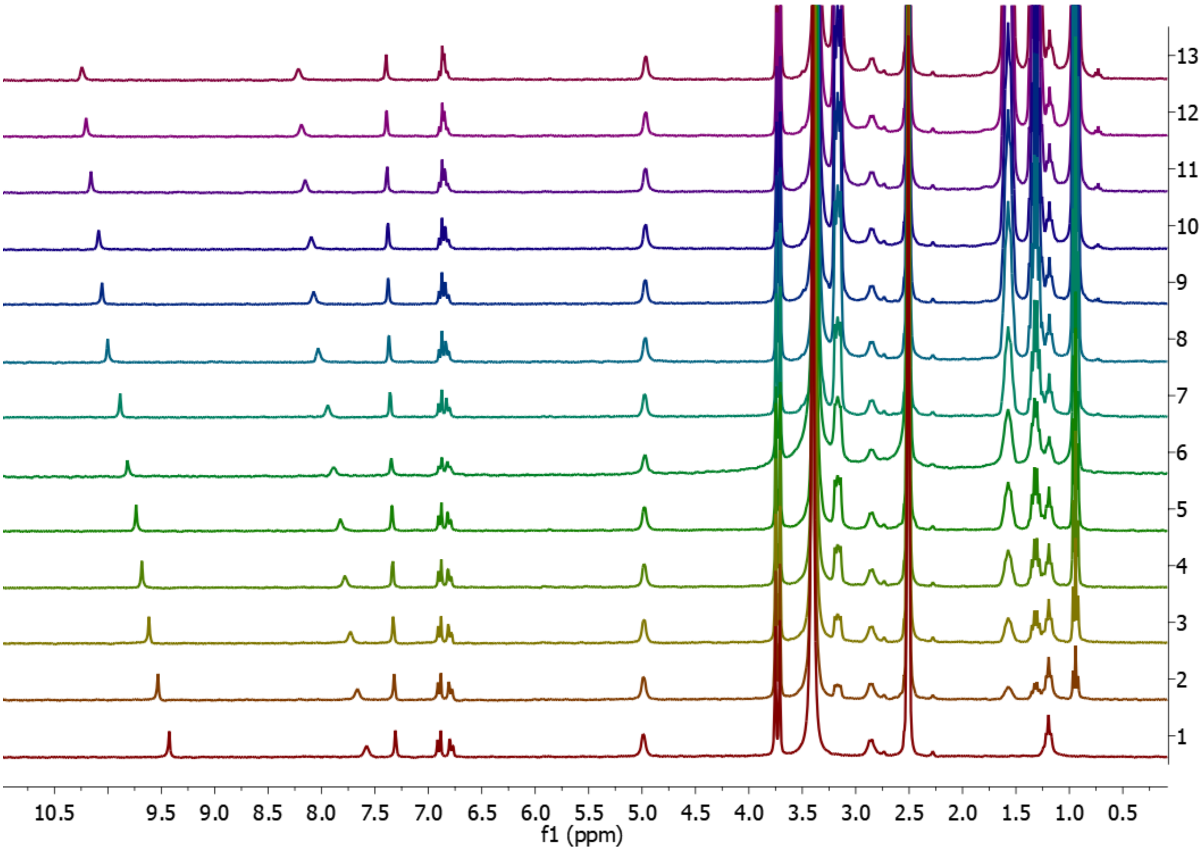

Fig. S37. ^{1}H NMR spectra recorded upon titration of receptor **2** in DMSO-d$_6$ with TBACl in the presence of 3 eq. KPF_6.

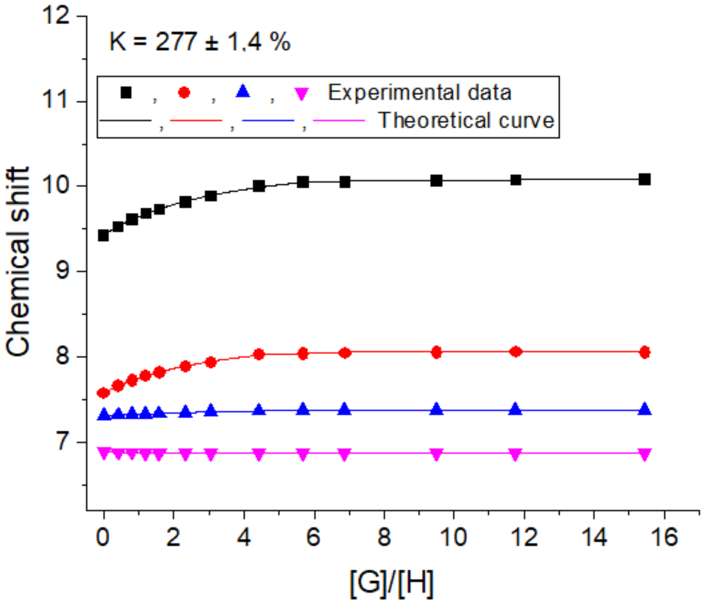

Fig. S38. ^{1}HNMR titration binding isotherms of receptor **2** in DMSO-d$_6$ upon addition of increasing amounts of TBACl in the presence of 3 eq. KPF_6.

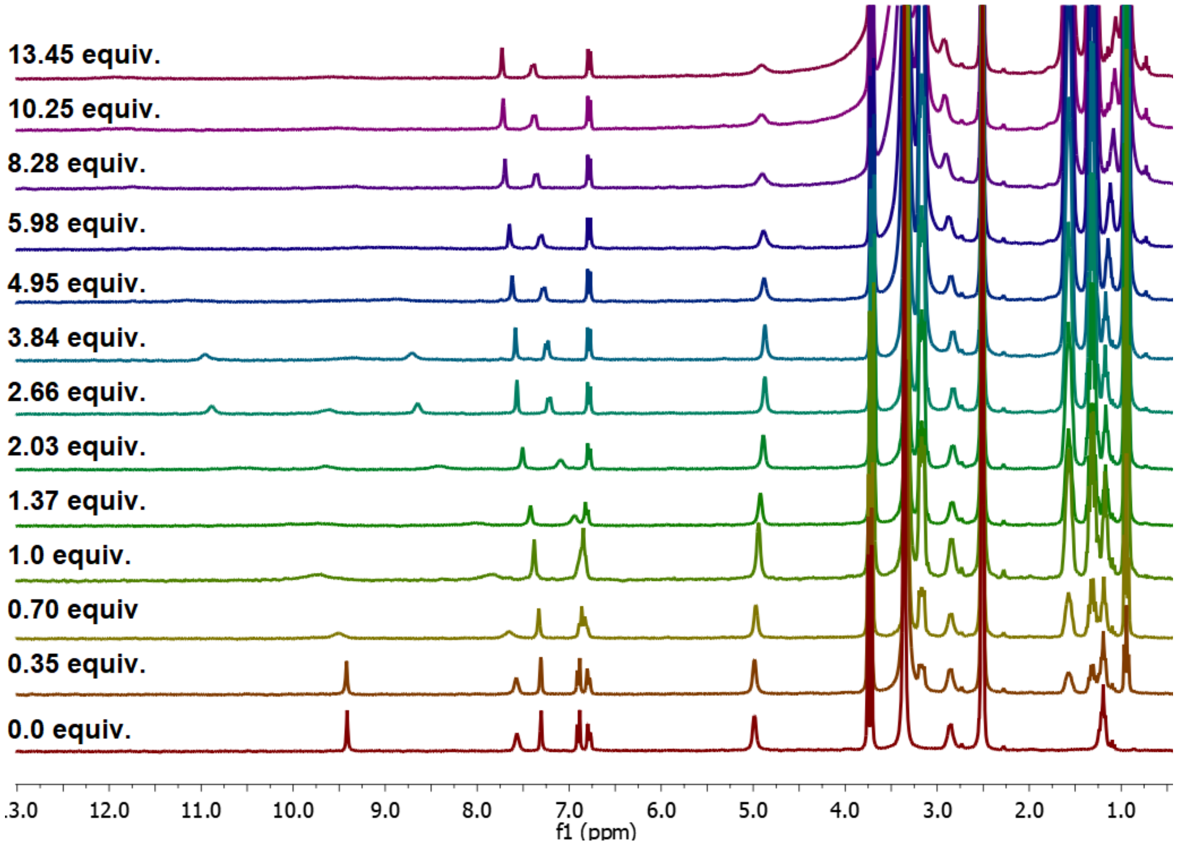

Fig. S39. ^{1}H NMR spectra recorded upon titration of receptor **2** in DMSO-d_6 with TBA_2SO4.

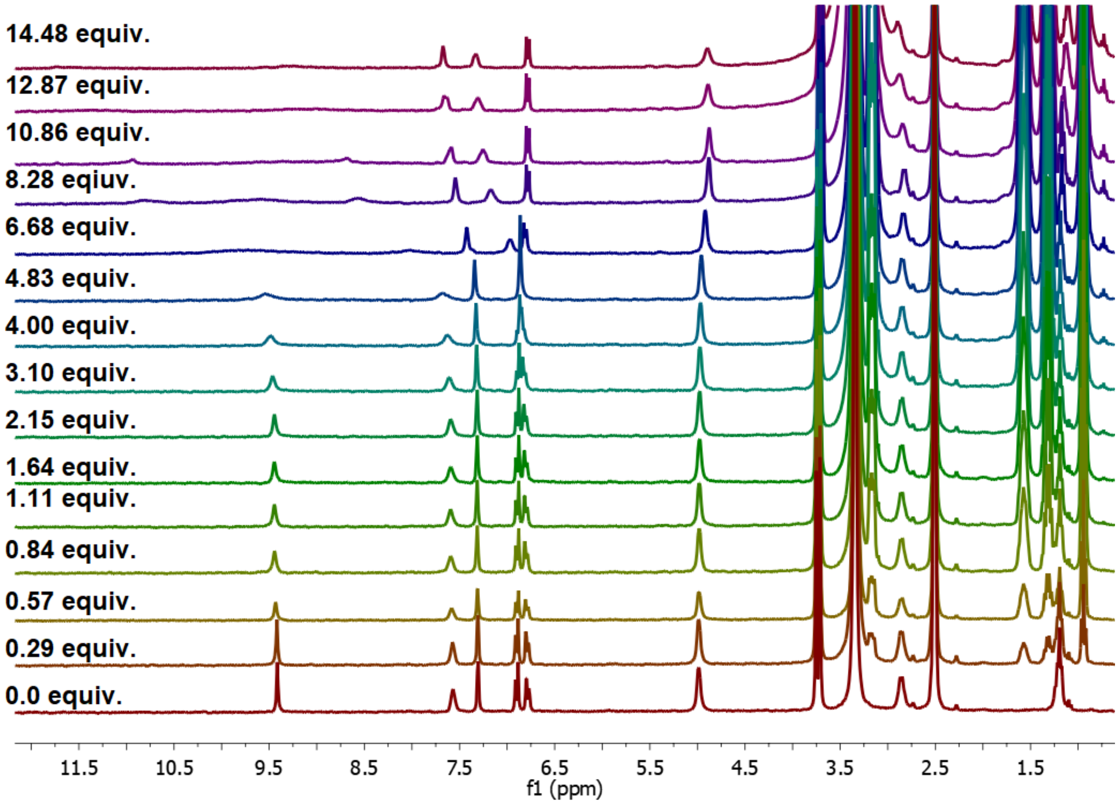

Fig. S40. ^{1}H NMR spectra recorded upon titration of receptor **2** in DMSO-d_6 with TBA_2SO_4 in the presence of 3 eq. KPF_6.

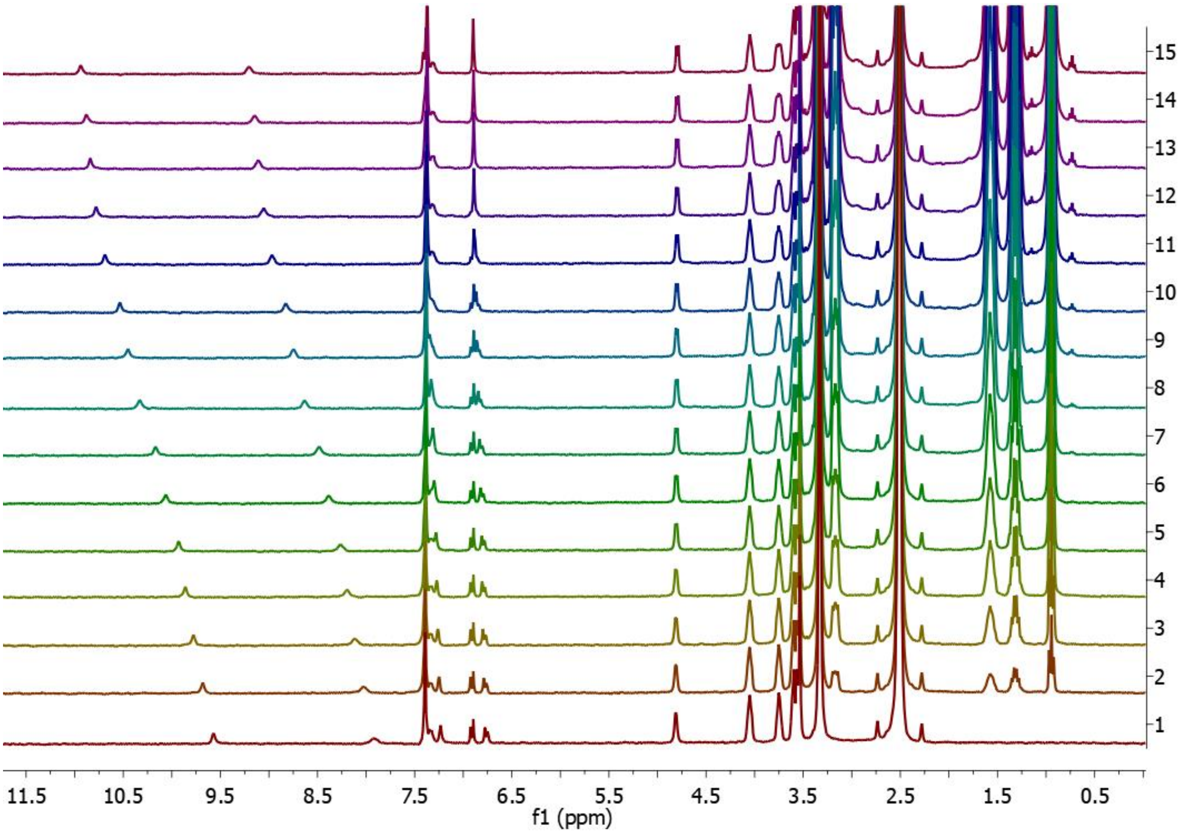

Fig. S41. ^{1}H NMR spectra recorded upon titration of receptor **3** in DMSO-d$_6$ with TBACl.

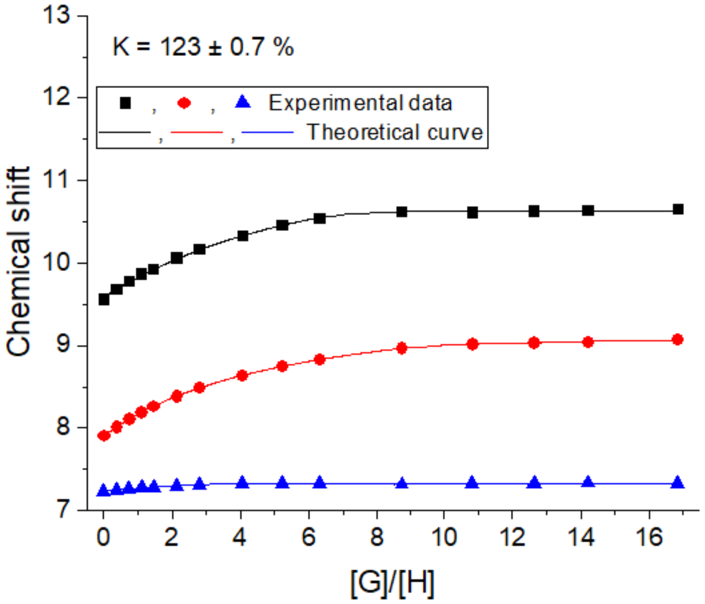

Fig. S42. ^{1}H NMR titration binding isotherms of receptor **3** in DMSO-d$_6$ upon addition of increasing amounts of TBACl.

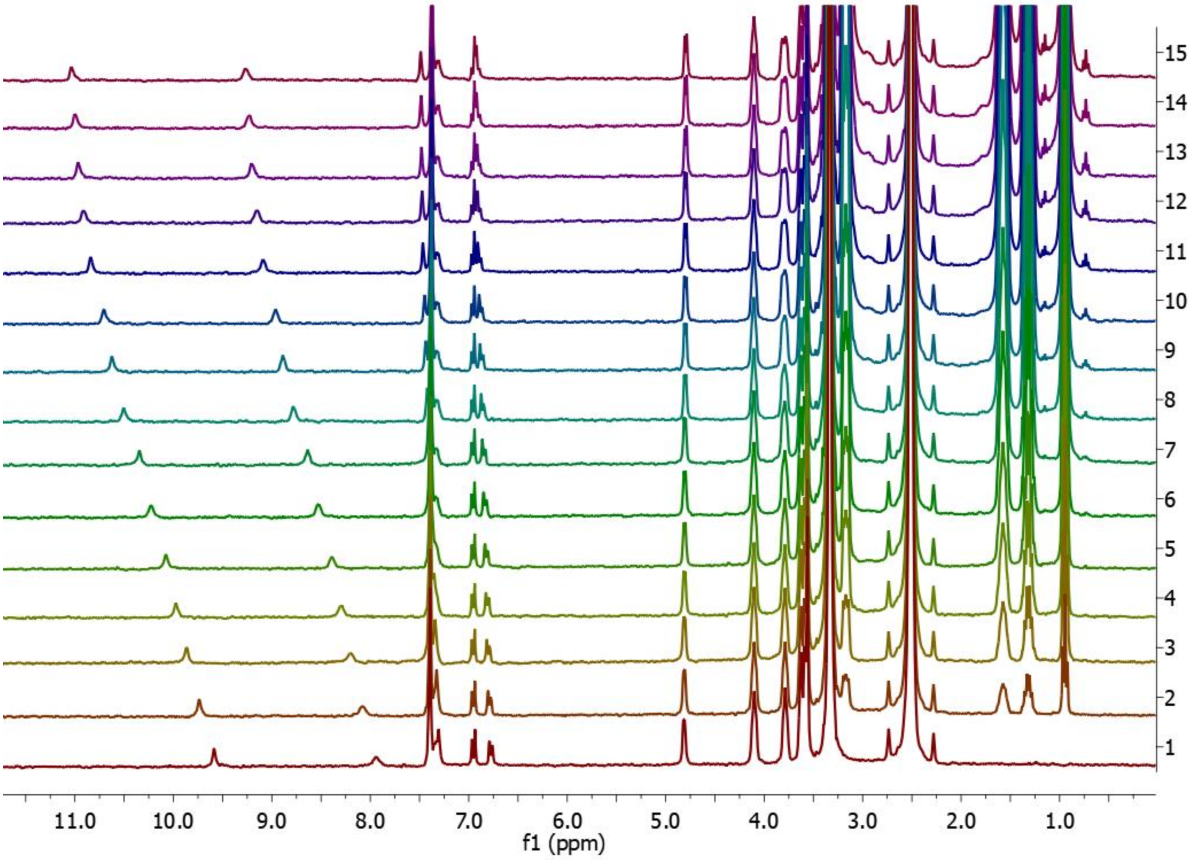

Fig. S43. ^{1}H NMR spectra recorded upon titration of receptor **3** in DMSO-d_6 with TBACl in the presence of 1 eq. KPF_6.

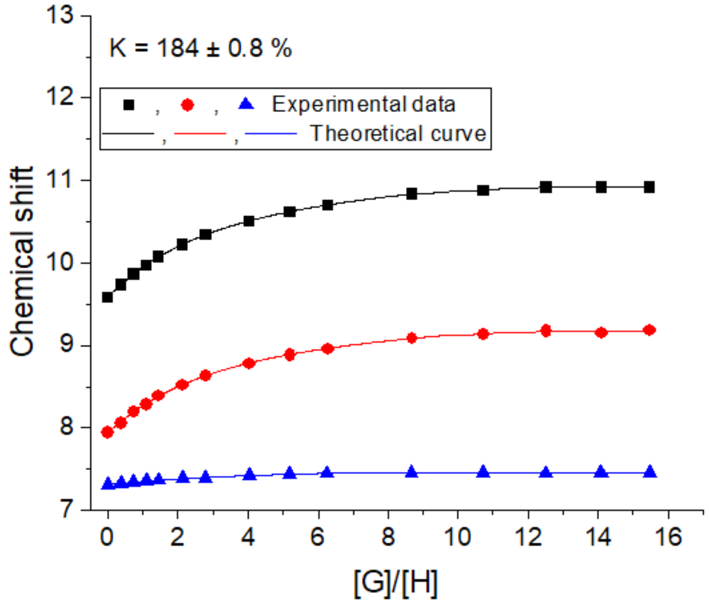

Fig. S44. ^{1}HNMR titration binding isotherms of receptor **3** in DMSO-$d_6$upon addition of increasing amounts of TBACl in the presence of 1 eq. KPF_6.

4. UV-vis titration experiments

UV-vis titration experiments were performed on a Thermo Spectronic Unicam UV 500 spectrophotometer in CH_3CN solution at 298K. To 10 mm cuvette was added 2.5 mL of freshly prepared (receptor **1** – c = 1.2x10^{-5}M , receptor **2** – c = 1.4x10^{-5} M, receptor **3** - c = 2.1x10^{-5} M) solution of studied receptor and in case of ion pair binding studies 1 mol equivalent of cation (KPF_6 or $NaClO_4$) was added prior titrations. Small aliquots of ca. 1.1×10^{-3} M TBAX solution containing receptor **1**, receptor **2** or receptor **3** at the same concentration as in cuvette, were added and a spectrum was acquired after each addition. The resulting titration data were analyzed using BindFit (v0.5) package, available online at http://supramolecular.org.

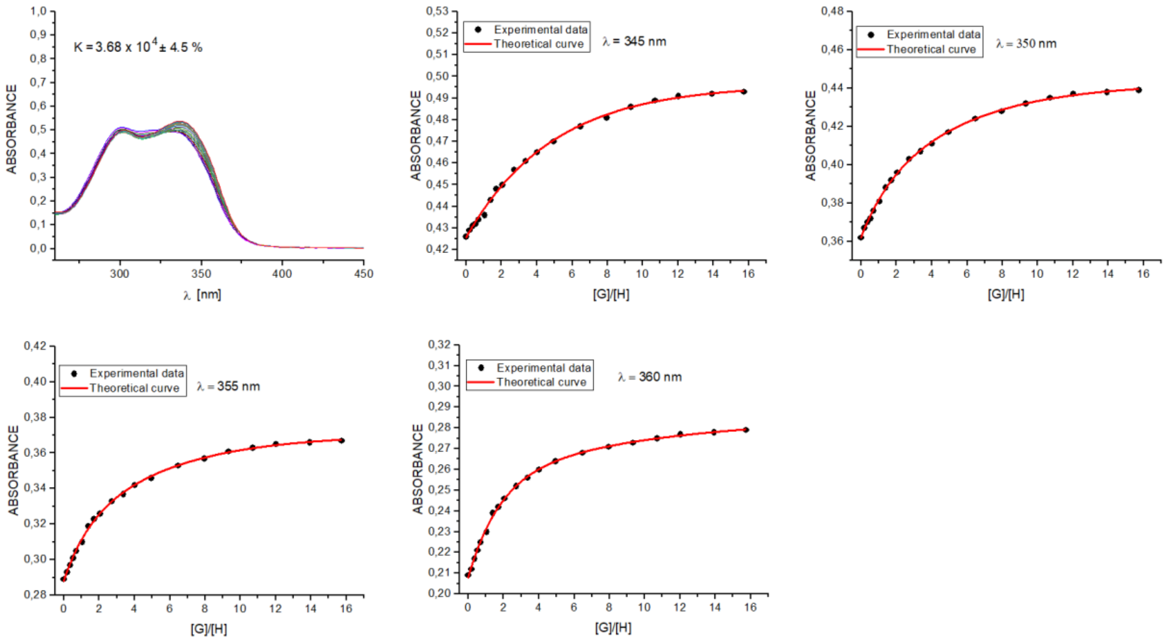

Fig. S45. UV-vis titration of receptor **1** with TBACl in CH_3CN and selected binding isotherms.

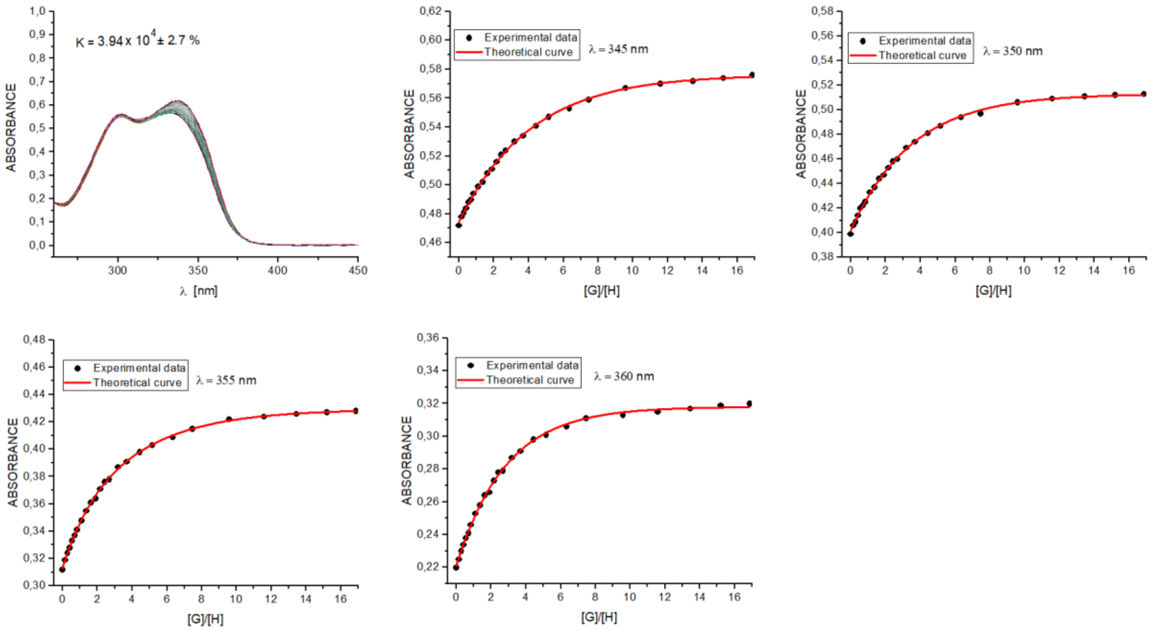

Fig. S46. UV-vis titration of receptor **1** with TBACl in the presence of 3 equivalent of $NaClO_4$ in CH_3CN and selected binding isotherms.

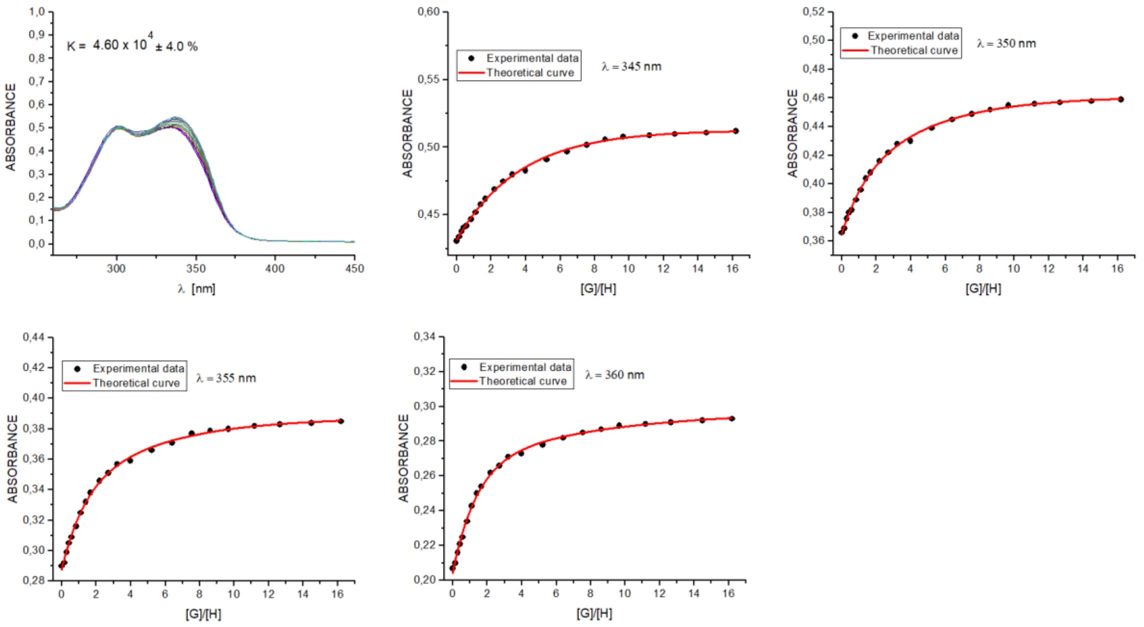

Fig. S47. UV-vis titration of receptor **1** with TBACl in the presence of 3 equivalent of KPF_6 in CH_3CN and selected binding isotherms.

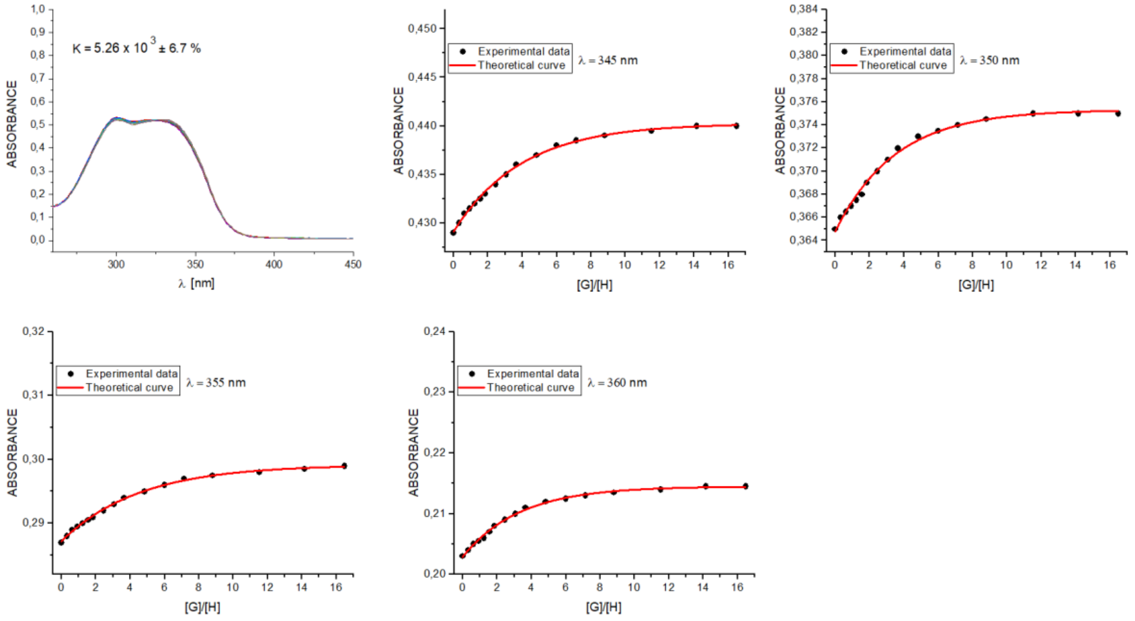

Fig. S48. UV-vis titration of receptor **1** with $TBANO_3$ in CH_3CN and selected binding isotherms.

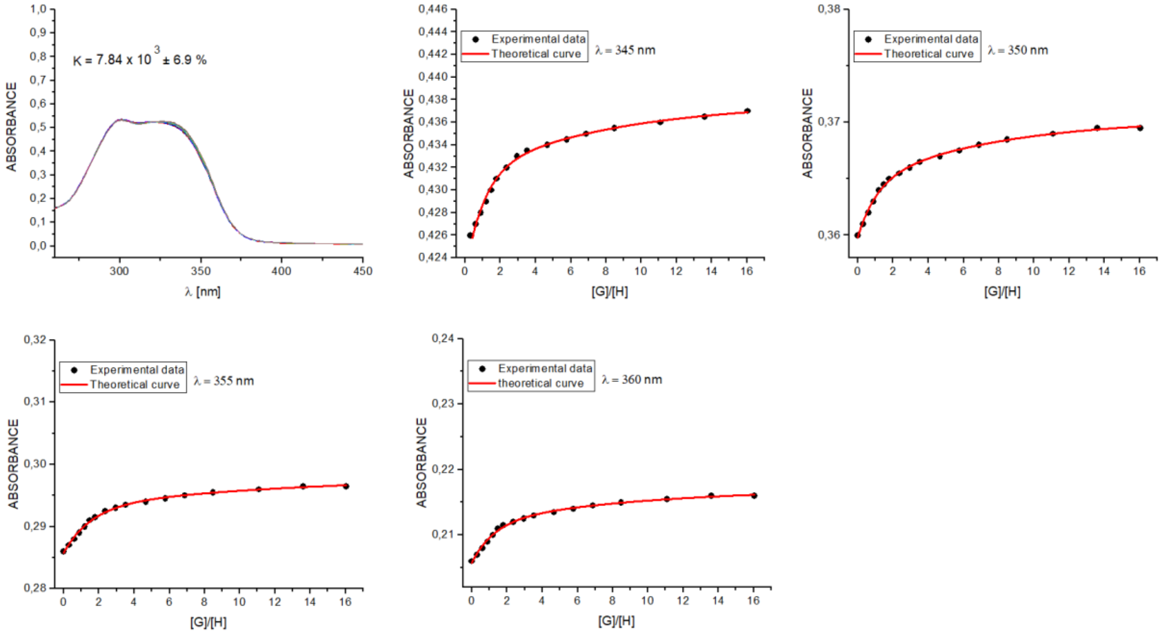

Fig. S49. UV-vis titration of receptor **1** with $TBANO_3$ in the presence of 3 equivalent of KPF_6 in CH_3CN and selected binding isotherms.

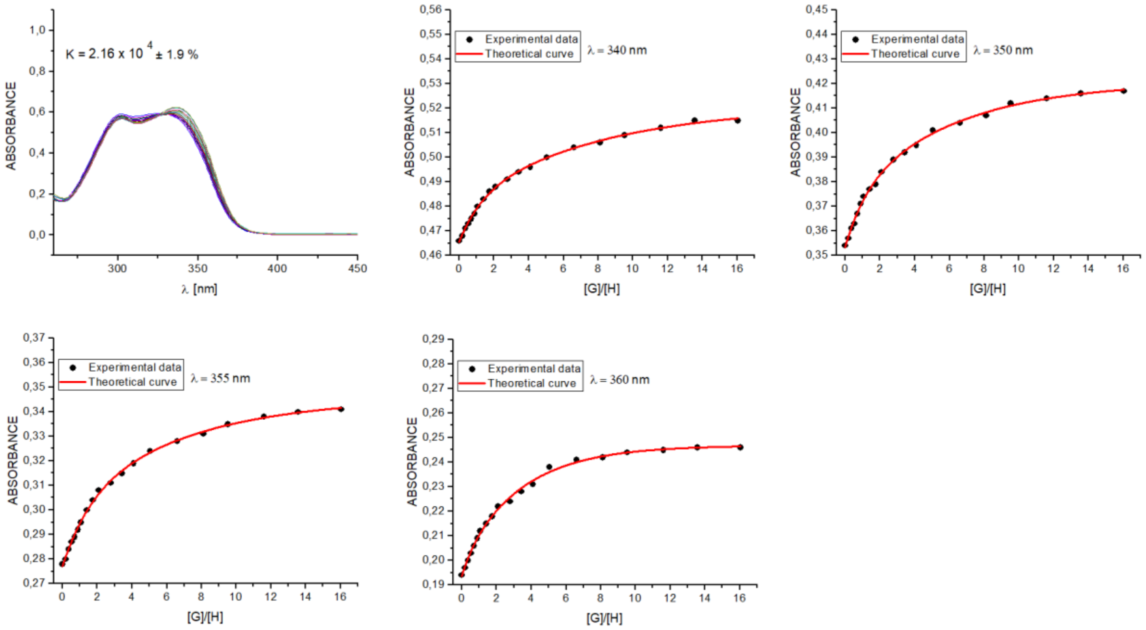

Fig. S50. UV-vis titration of receptor **1** with $TBANO_2$ in CH_3CN and selected binding isotherms.

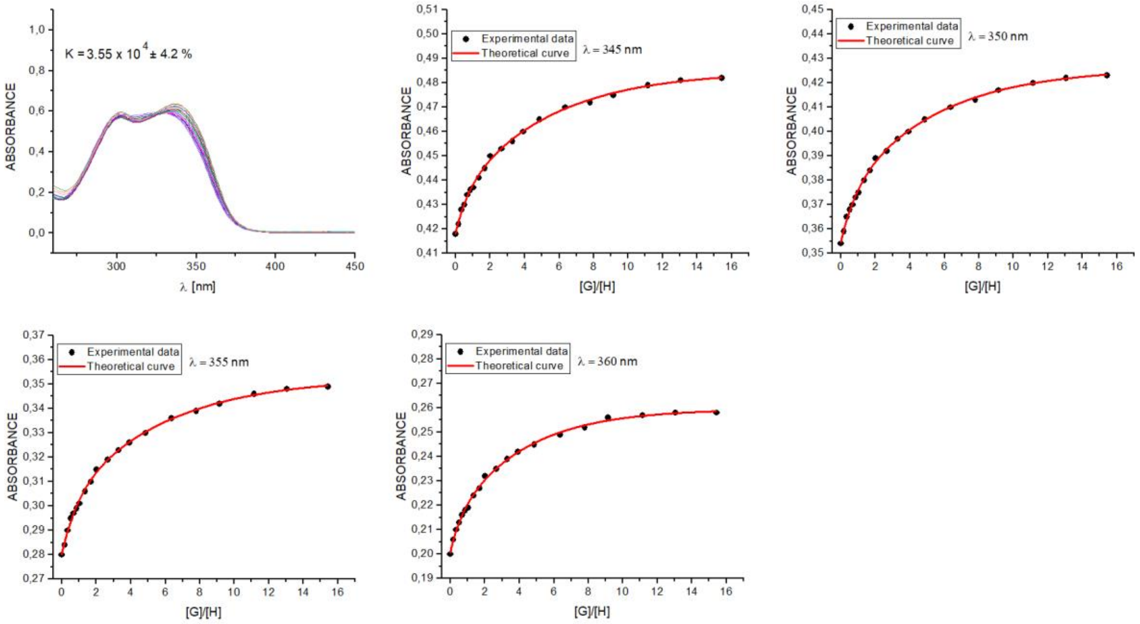

Fig. S51. UV-vis titration of receptor **1** with $TBANO_2$ in the presence of 3 equivalent of KPF_6 in CH_3CN and selected binding isotherms.

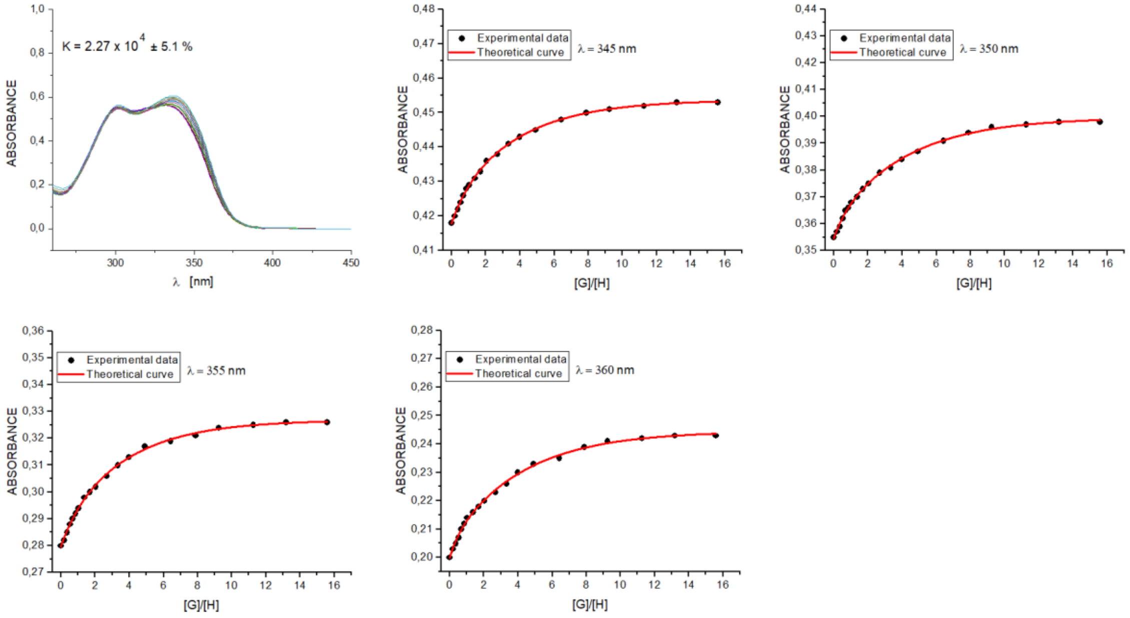

Fig. S52. UV-vis titration of receptor **1** with TBABr in CH_3CN and selected binding isotherms.

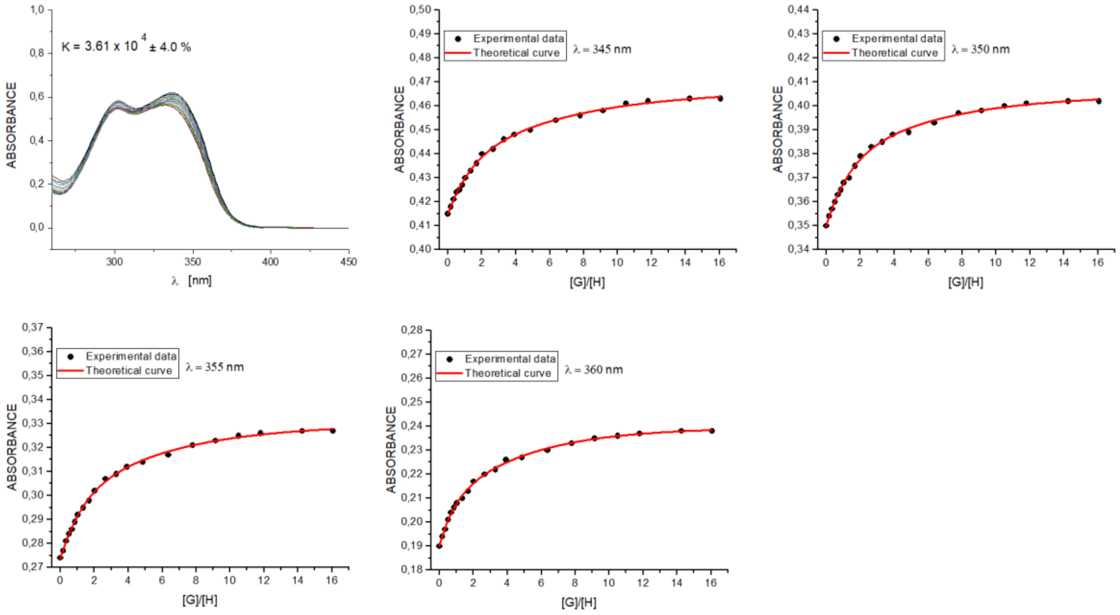

Fig. S53. UV-vis titration of receptor **1** with TBABr in the presence of 3 equivalent of KPF_6 in CH_3CN and selected binding isotherms.

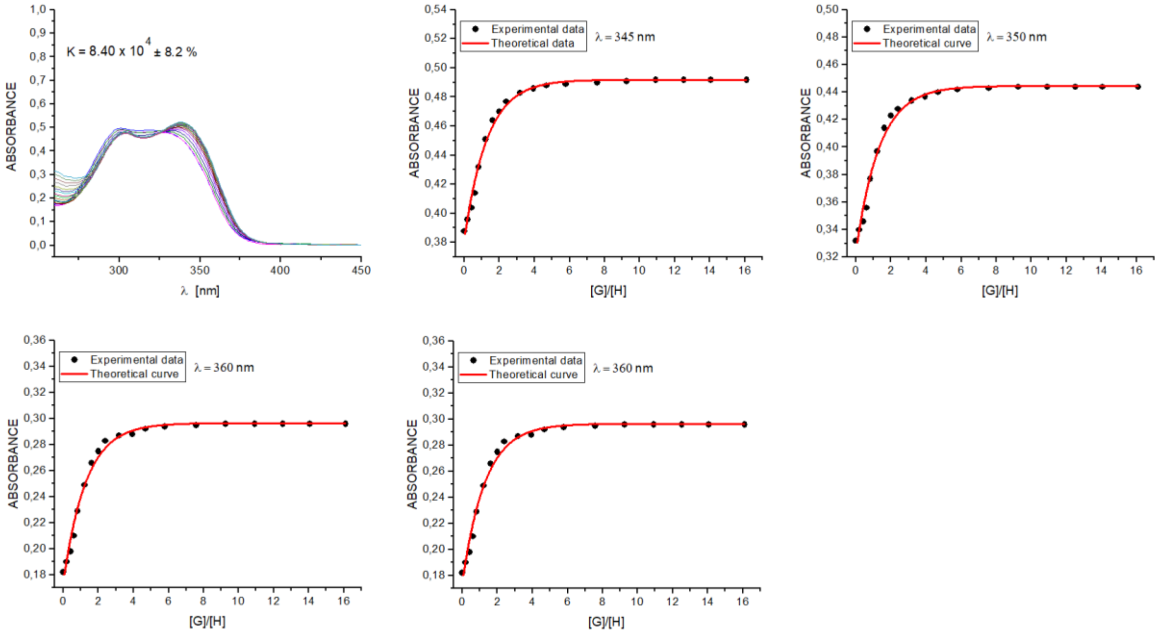

Fig. S54. UV-vis titration of receptor **1** with TBAPhCOO in CH_3CN and selected binding isotherms.

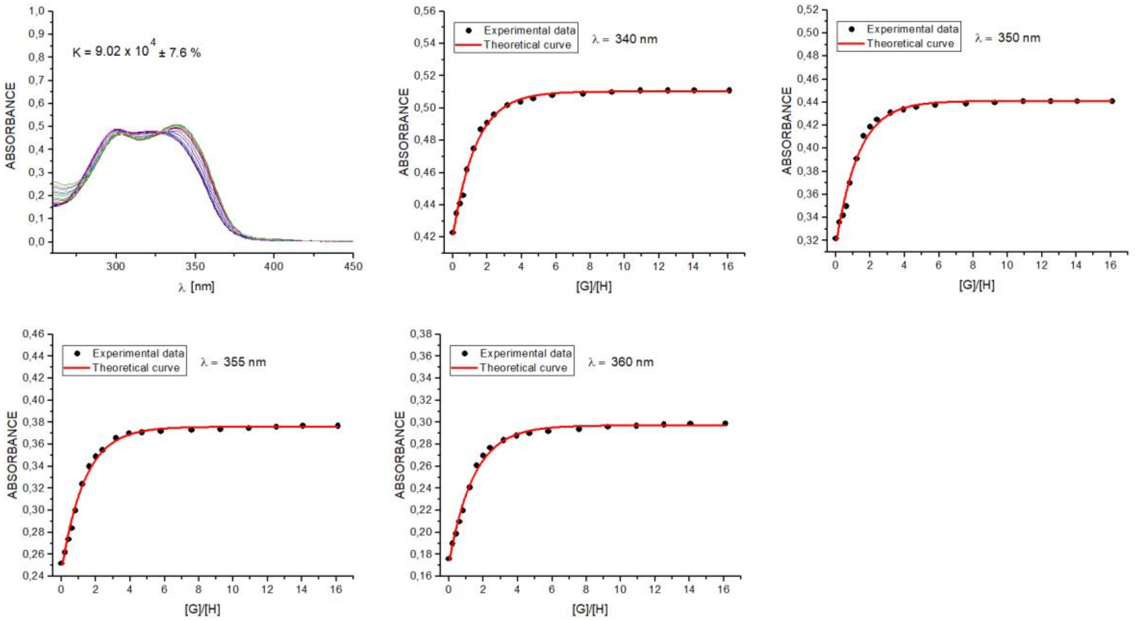

Fig. S55. UV-vis titration of receptor **1** with TBAPhCOO in the presence of 3 equivalent of KPF_6 in CH_3CN and selected binding isotherms.

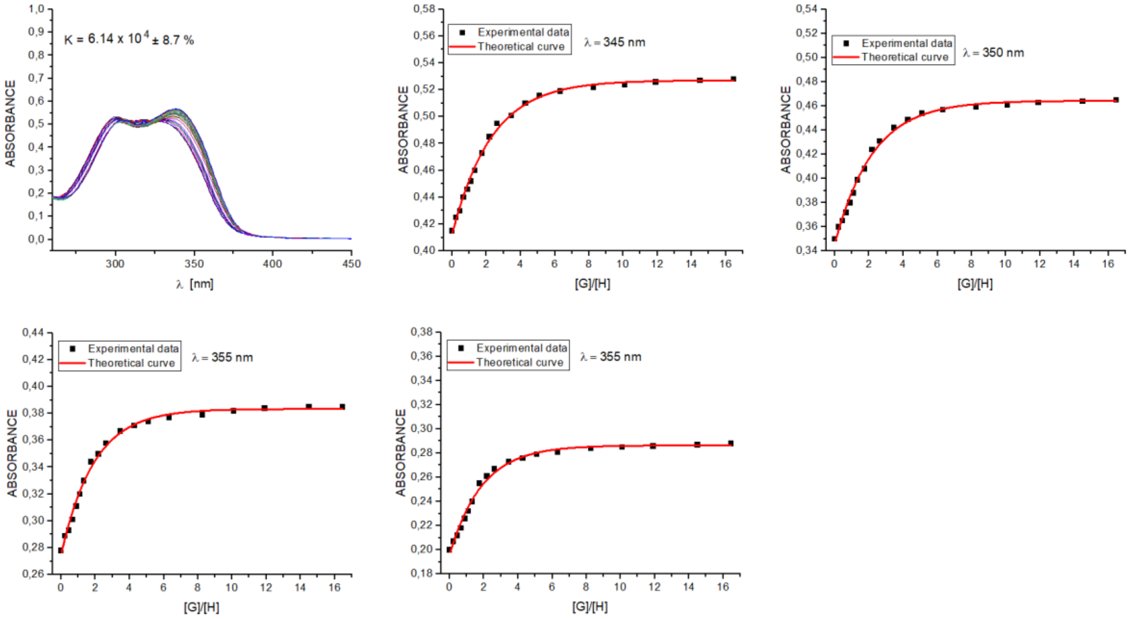

Fig. S56. UV-vis titration of receptor **1** with TBACH_3COO in CH_3CN and selected binding isotherms.

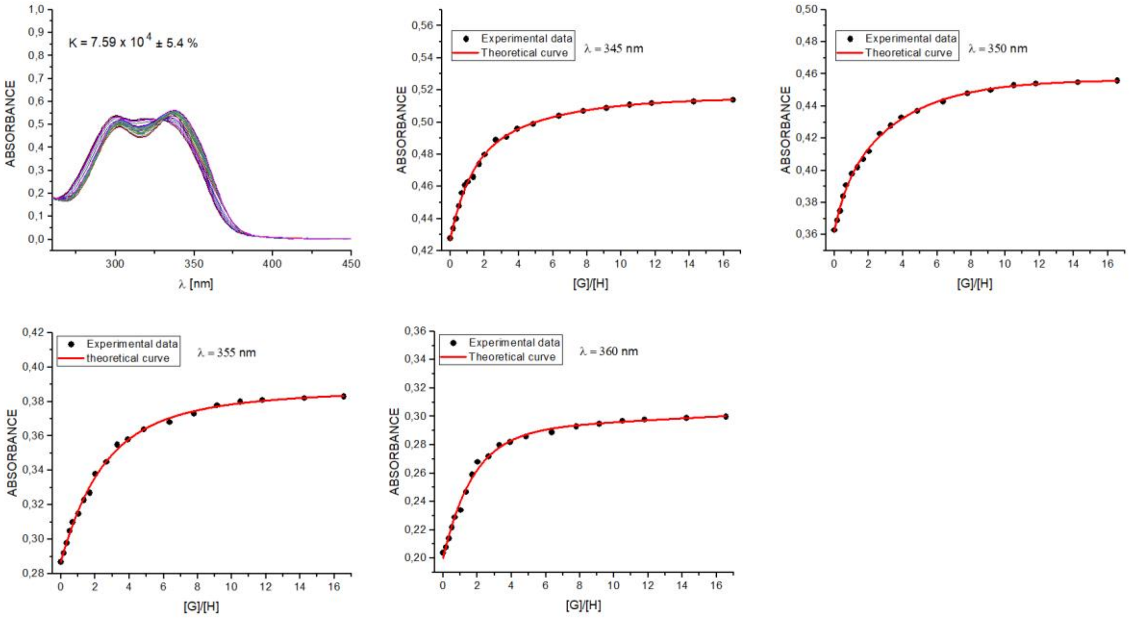

Fig. S57. UV-vis titration of receptor **1** with TBACH_3COO in the presence of 3 equivalent of KPF_6 in CH_3CN and selected binding isotherms.

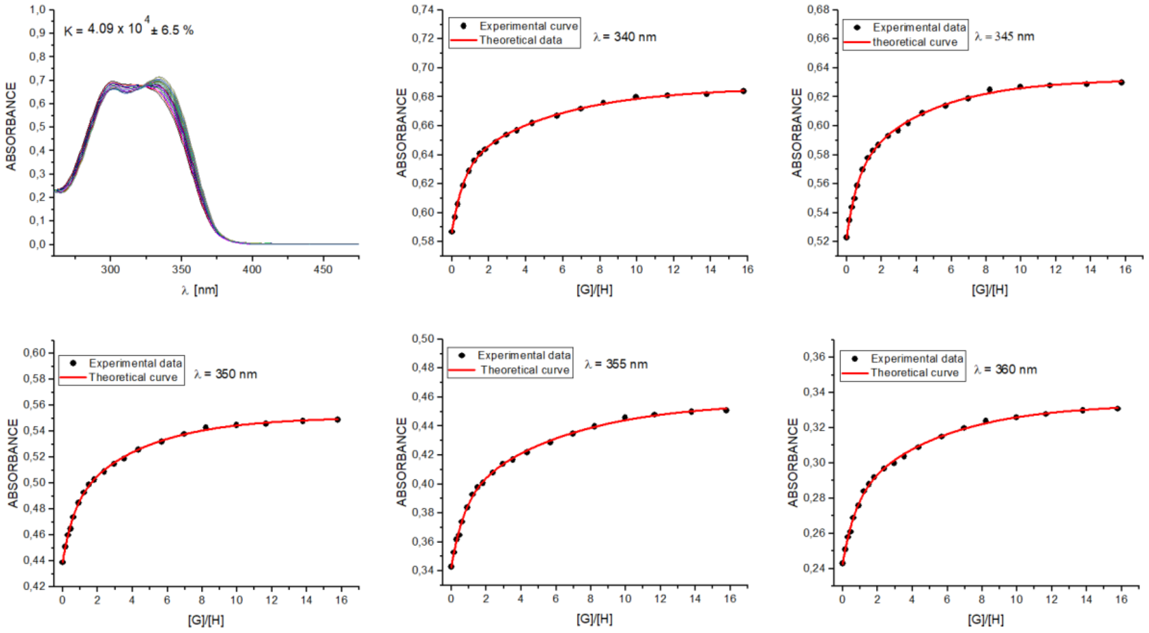

Fig. S58. UV-vis titration of receptor **2** with TBACl in CH_3CN and selected binding isotherms.

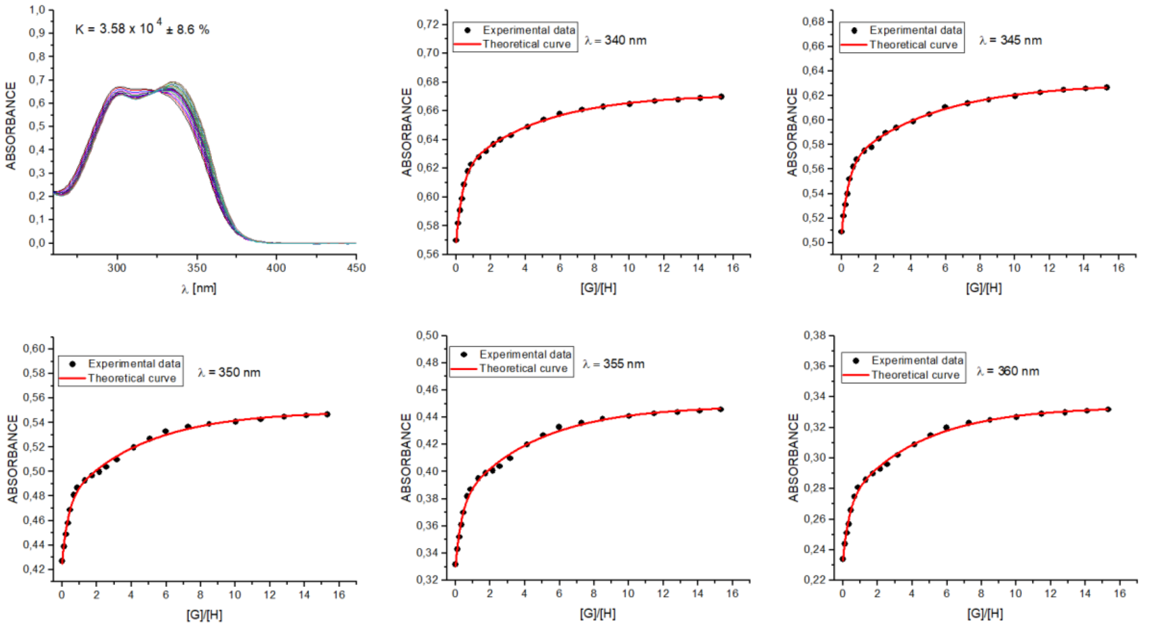

Fig. S59. UV-vis titration of receptor **2** with TBACl in the presence of 3 equivalent of KPF_6 in CH_3CN and selected binding isotherms.

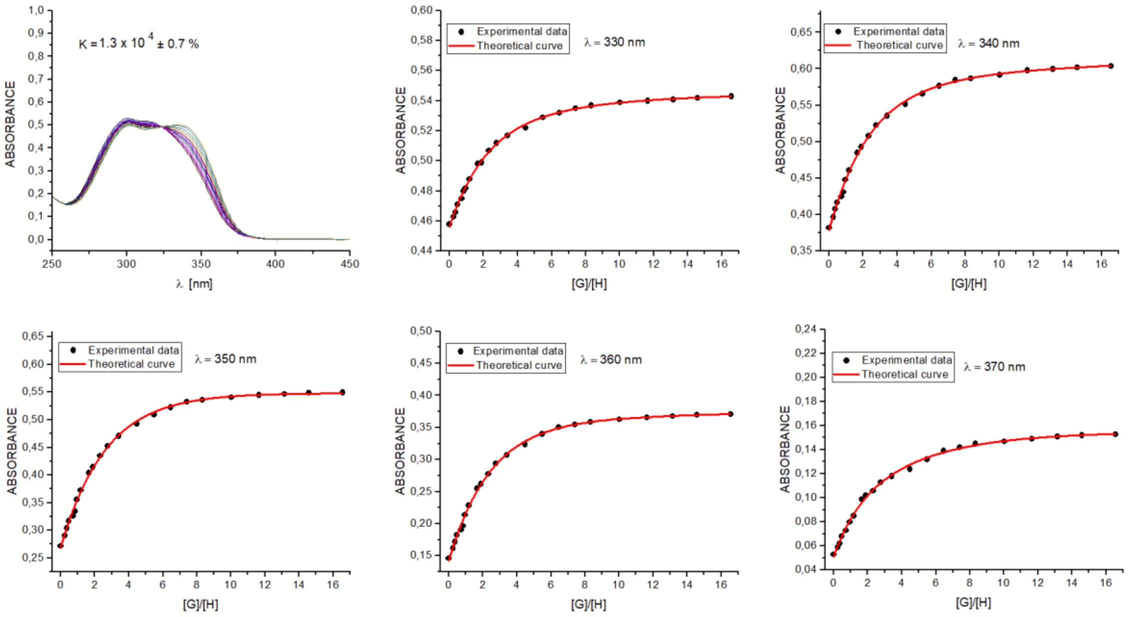

Fig. S60. UV-vis titration of receptor **3** with TBACl in CH_3CN and selected binding isotherms.

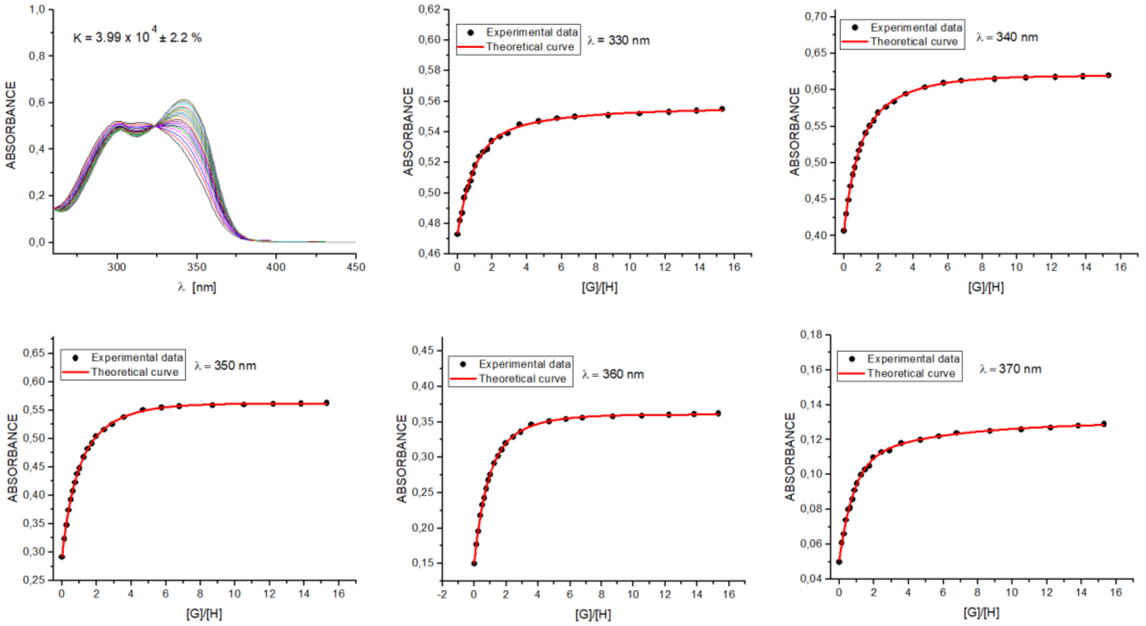

Fig. S61. UV-vis titration of receptor **3** with TBACl in the presence of 1 equivalent of KPF_6 in CH_3CN and selected binding isotherms.

5. DOSY, ROESY and HSQC experiments

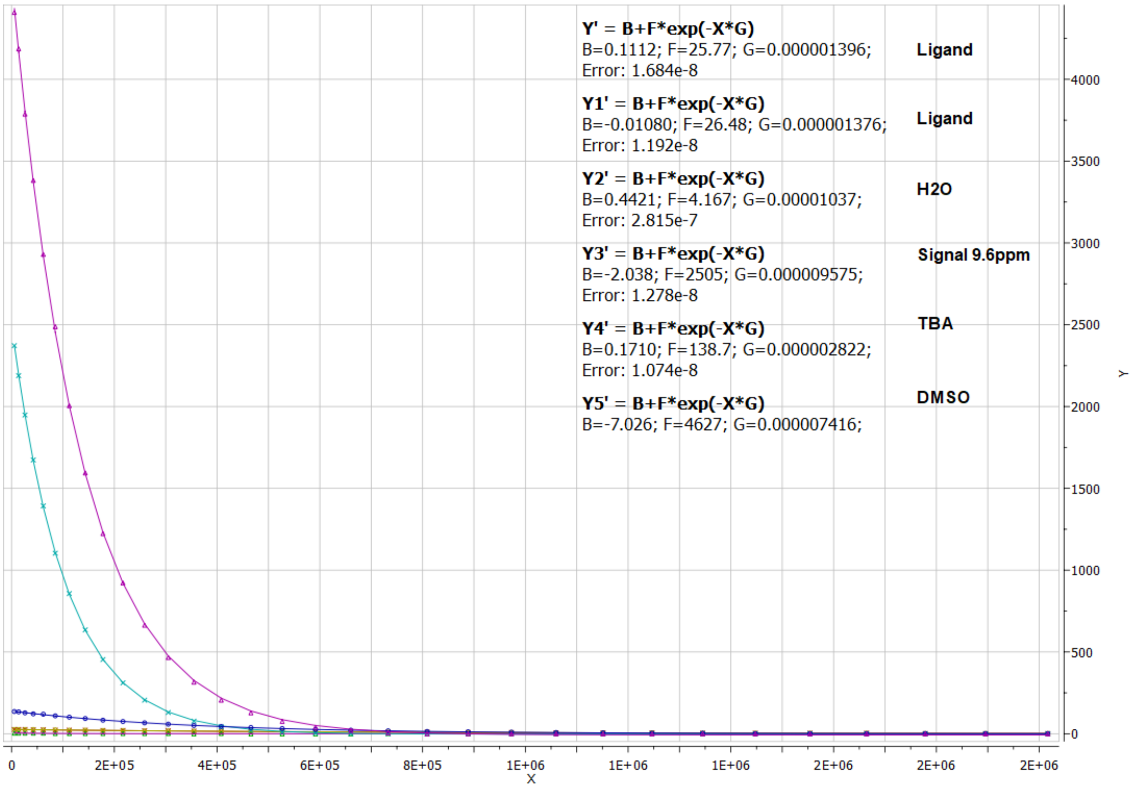

Fig. S62. DOSY experiment of Receptor **2** (1.5 mM) + 2.5 eq. TBA_2SO_4 in DMSO-d_6.

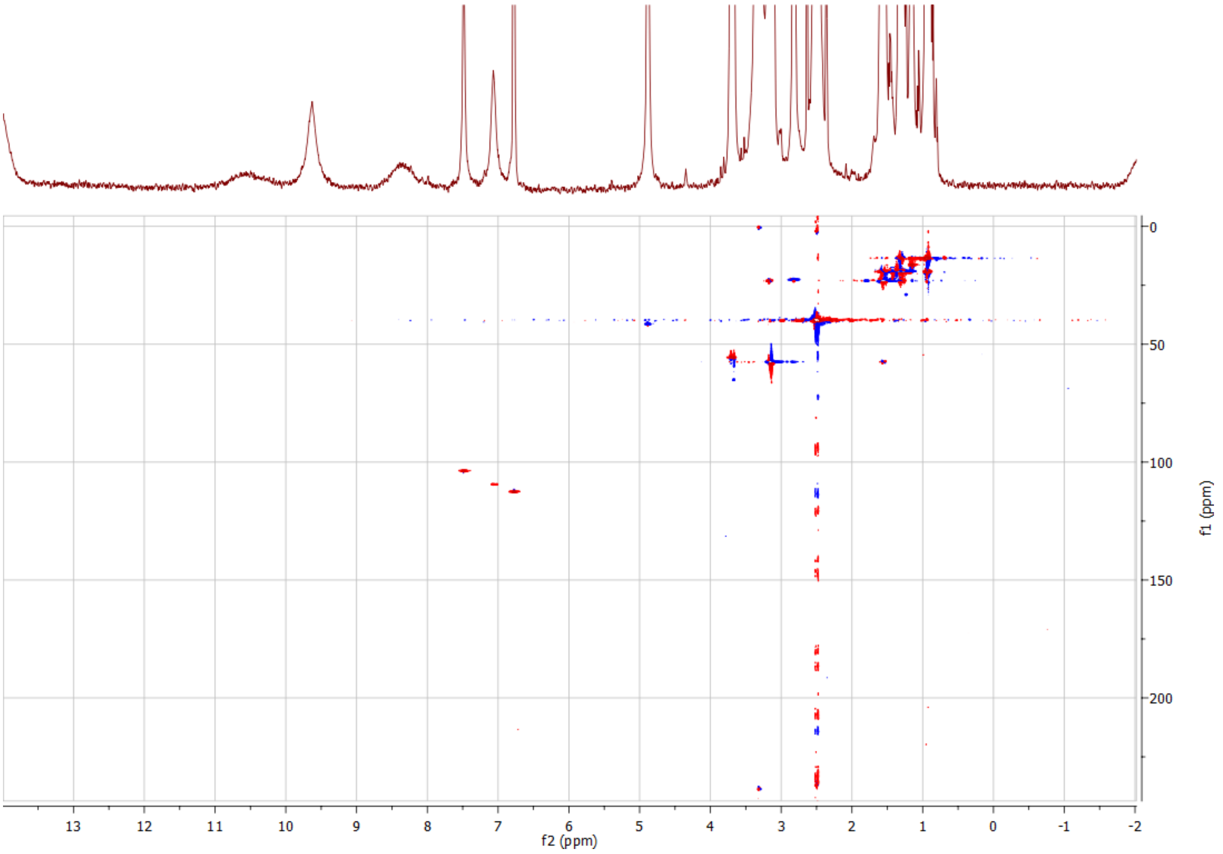

Fig. S63. ROESY NMR spectrum of Receptor **2** (1.5 mM) + 2.5 eq. TBA_2SO_4 in DMSO-d_6.

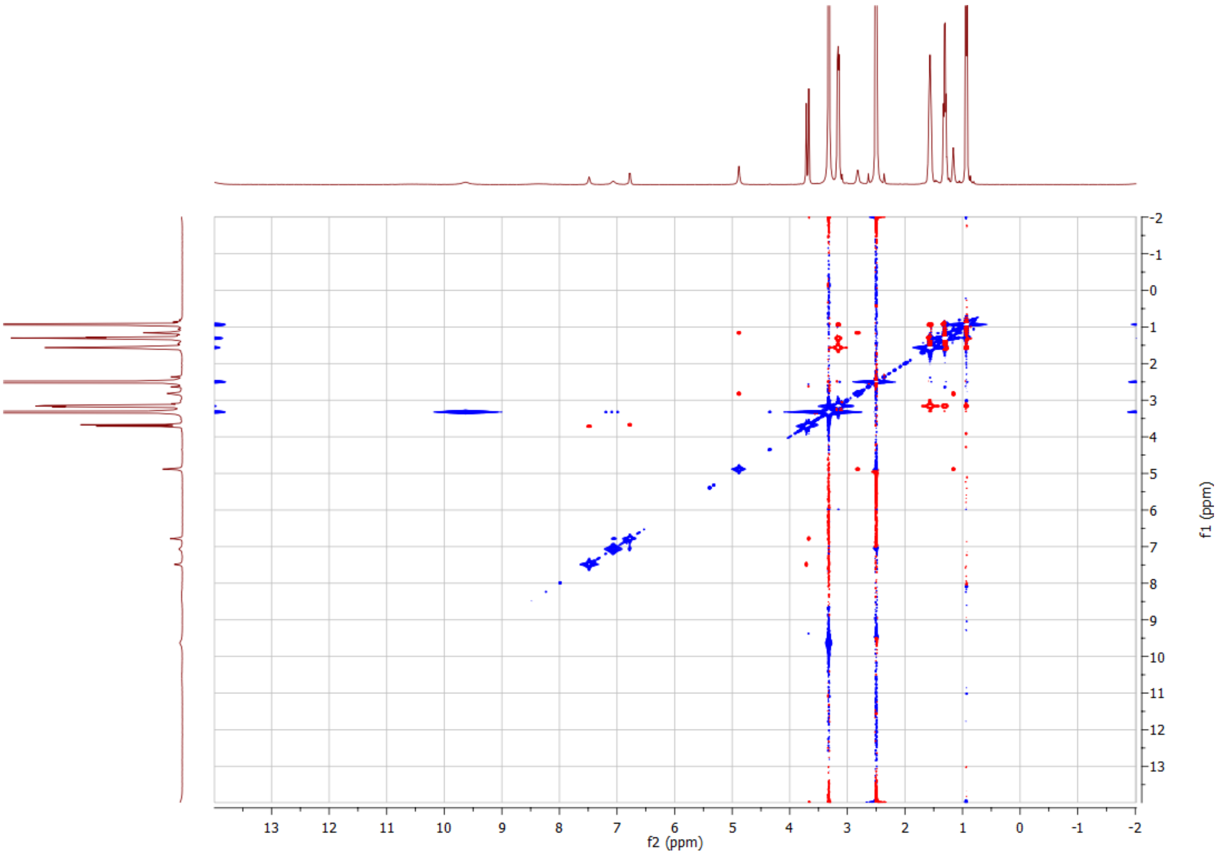

Fig. S64. HSQC NMR spectrum of Receptor **2** (1.5 mM) + 2.5 eq. TBA_2SO_4 in DMSO-d_6.

6. Extraction experiments

General procedure for Solid Liquid Extraction (SLE): A solution of receptor **1** in acetonitrile (1 ml, 5 mM) was intensive shaking overnight with potassium solid salts. Then 0,5 mL of acetonitrile solution phase was taken and 20-fold diluted deionized water. The concentration of anions in aqueous phase was determined by high performance ion chromatography (HPIC).

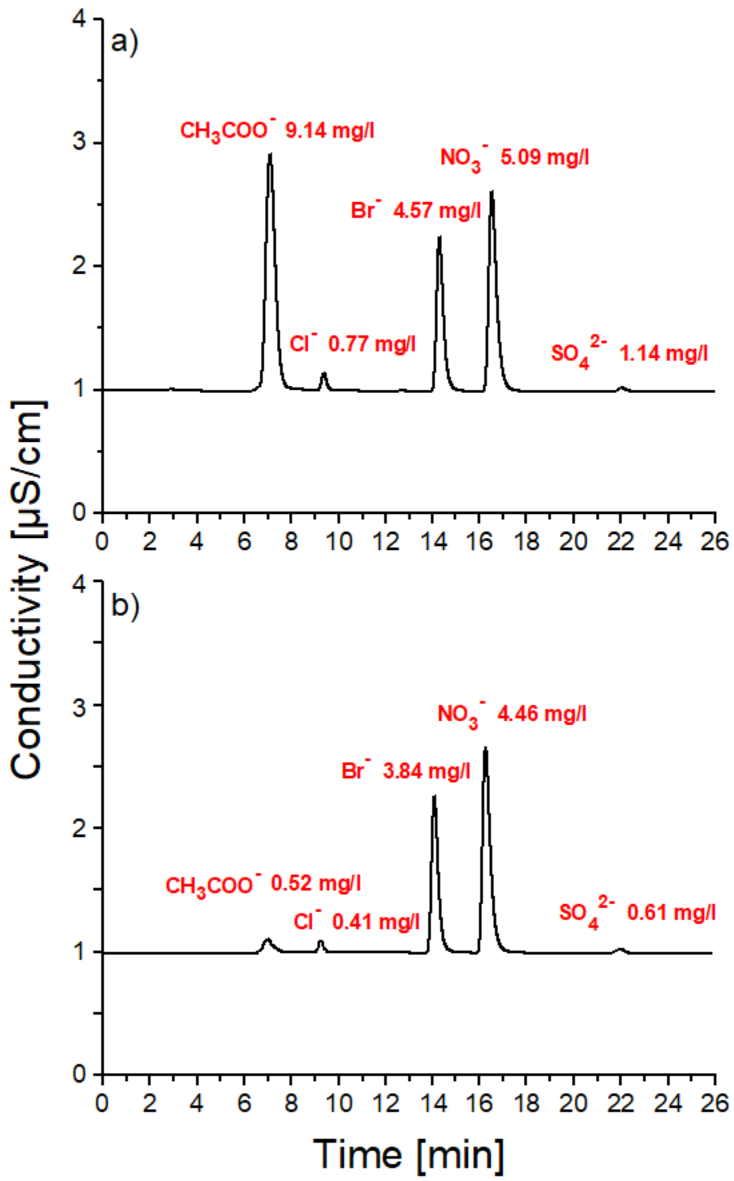

Fig. S65. Chromatograms obtained after SLE of potassium salts (20-fold dilution) (a) with receptor **1** (5mM) in CH_3CN (b) with CH_3CN.

General procedure for Liquid Liquid Extraction (LLE): A solution of receptor in chloroform (2 ml, 5 mM) was shaken vigorously with aqueous mixture (no pH adjustment, pH depending of the salts used; above pH 8 there is no phase separation probably due to the receptor deprotonation. This eliminates direct use of basic salts such as hydrogen phosphates or phosphates) of suitable salts 1 mM each (2 ml) for overnight. Then 1 mL of aqueous phase was taken and tenfold diluted. The concentration of anions in aqueous phase was determined by high performance ion chromatography (HPIC).

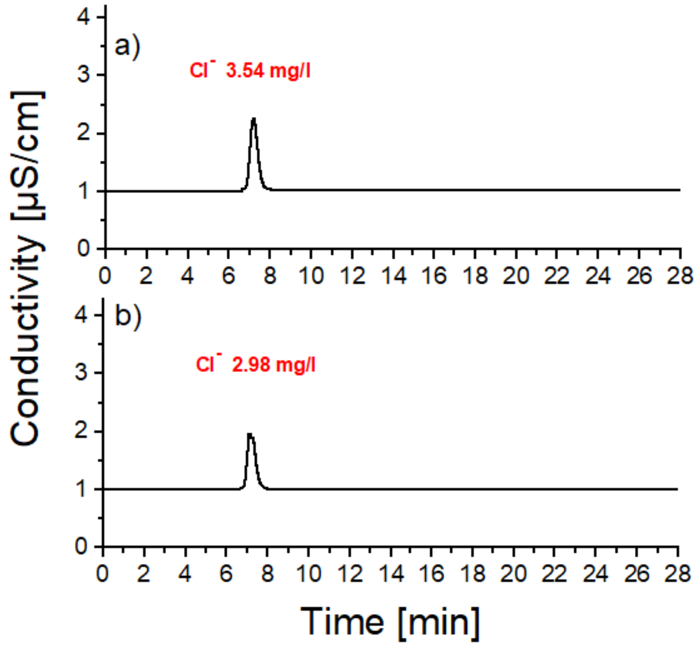

Fig. S66. Chromatograms obtained during extraction experiments after tenfold dilution: (a) source phase, (b) after extraction an aqueous solution of KCl (1 mM) with 5 mM of receptor **1** in $CHCl_3$.

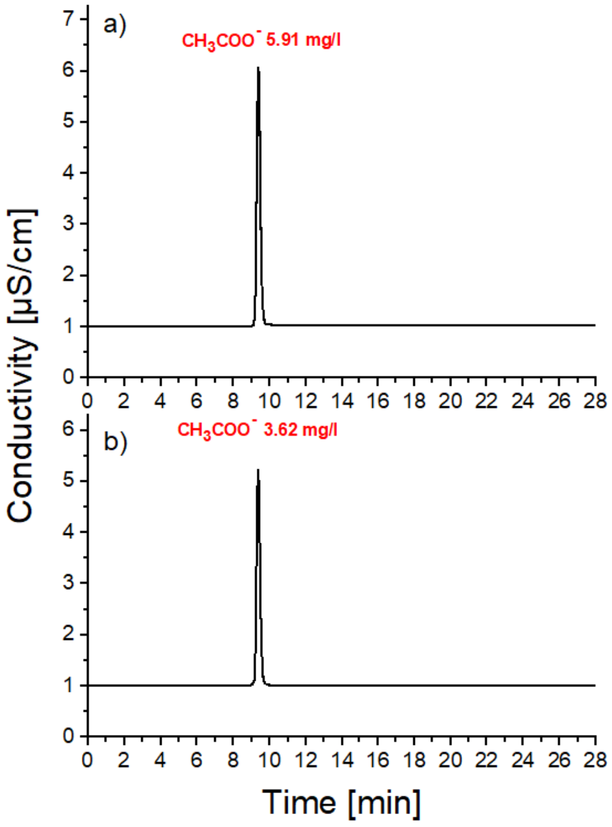

Fig. S67. Chromatograms obtained during extraction experiments after tenfold dilution: (a) source phase, (b) after extraction an aqueous solution of CH_3COOK (1 mM) with 5 mM of receptor **1** in $CHCl_3$.

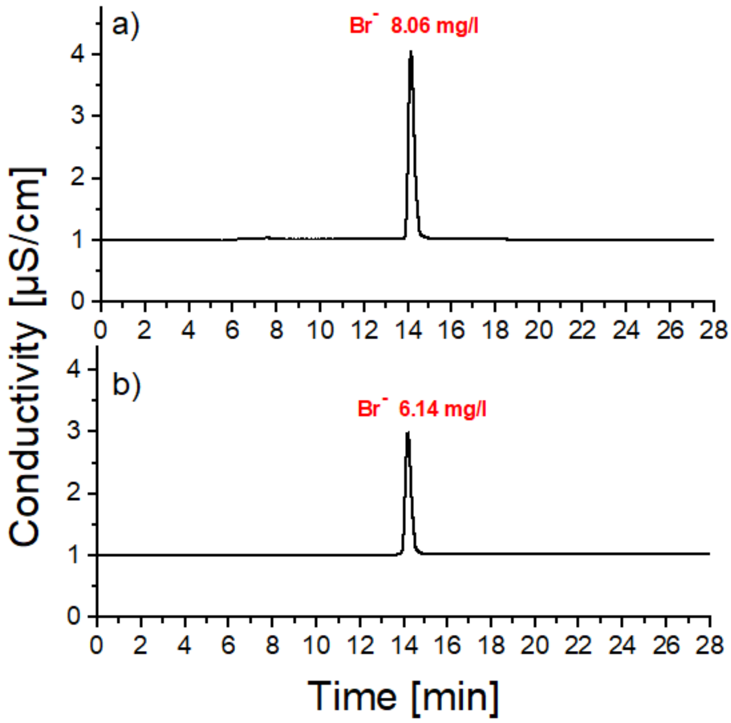

Fig. S68. Chromatograms obtained during extraction experiments after tenfold dilution: (a) source phase, (b) after extraction an aqueous solution of KBr (1 mM) with 5 mM of receptor **1** in $CHCl_3$.

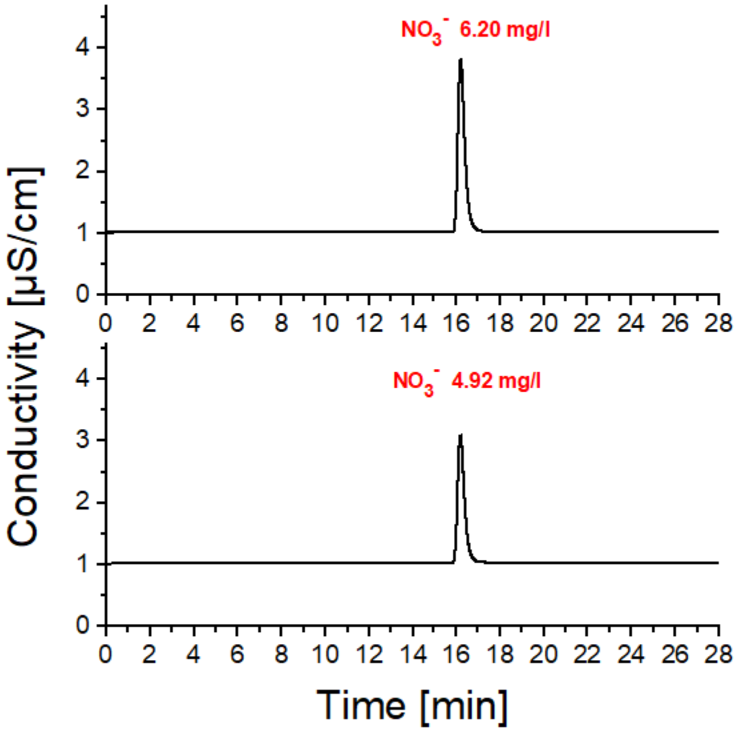

Fig. S69. Chromatograms obtained during extraction experiments after tenfold dilution: (a) source phase, (b) after extraction an aqueous of KNO_3 (1 mM) with 5 mM of receptor **1** in $CHCl_3$.

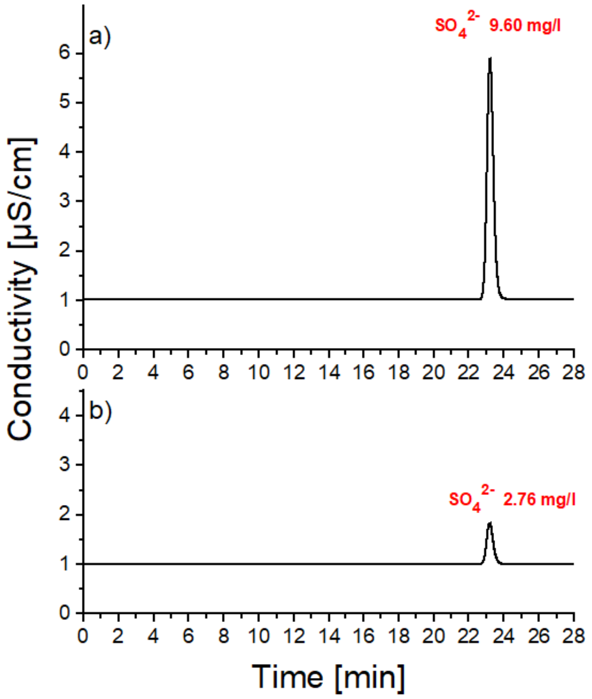

Fig. S70. Chromatograms obtained during extraction experiments after tenfold dilution: (a) source phase, (b) after extraction an aqueous of K_2SO_4 (1 mM) with 5 mM of receptor **1** in $CHCl_3$.

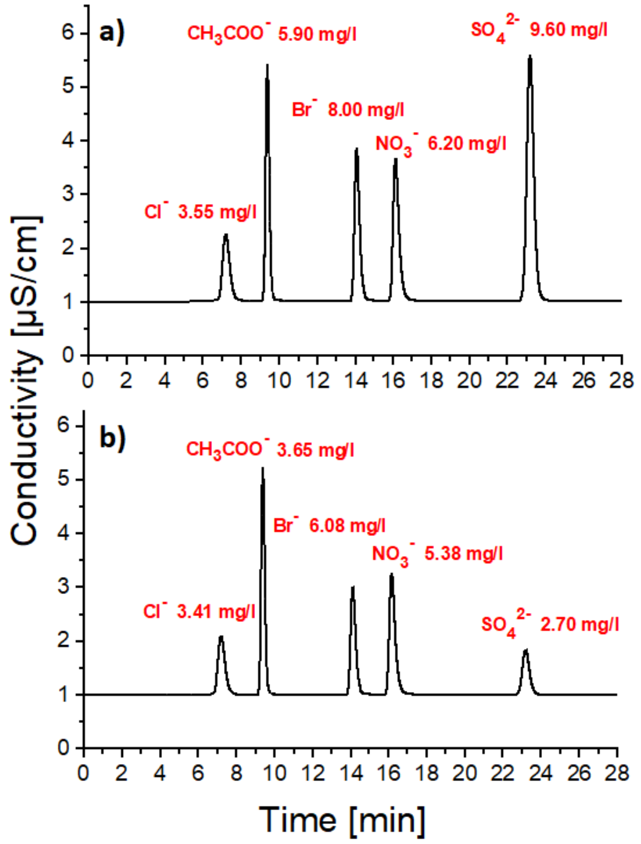

Fig. S71. Chromatograms obtained during extraction experiments after tenfold dilution: (a) source phase, (b) after extraction with 5 mM of receptor **1** in $CHCl_3$.

Table S1. Summarized data for competitive LLE. Source phase: aqueous mixture of potassium salts (1 mM each); Organic phase: Receptor **1**, **2** or **3** (5 mM) in $CHCl_3$.

Anion	Source phase [mg/l]	After extraction with receptor 1 [mg/l]	After extraction with receptor 2[a] [mg/l]	After extraction with receptor 3 [mg/l]
Cl^-	3.55	3.41	3.50	3.52
CH_3COO^-	5.90	3.65	5.76	5.80
Br^-	8.00	6.08	7.96	7.88
NO_3^-	6.20	5.38	5.98	6.02
SO_4^{2-}	9.60	2.70	9.36	9.22

[a] receptor **2** is poorly soluble in chloroform, suspension of **2** in $CHCl_3$ was used in LLE and no complete phase separation was noted.

7. Crystal data

1 + Na_triFlac. The X-ray measurement of **1 + Na_triFlac** was performed at 130.0(5) K on a Bruker D8 Venture PhotonII diffractometer equipped with a TRIUMPH monochromator and a MoKα fine focus sealed tube (λ = 0.71073 Å). A total of 2930 frames were collected with Bruker APEX3 program [S1]. The frames were integrated with the Bruker SAINT software package [S2] using a narrow-frame algorithm. The integration of the data based on two domains and using a triclinic unit cell yielded a total of 208133 reflections up to 2θ equal 50.96°, of which 21096 were independent (average redundancy 9.866, completeness = 98.4%, R_{sig} = 4.70%) and 93633 were greater than $3\sigma(F^2)$. The final cell constants of a = 13.9030(10) Å, b = 19.1752(13) Å, c = 24.2017(16) Å, α = 106.491(2)°, β = 98.390(2)°, γ = 105.570(2)°, V = 5784.7(7) Å^3, are based upon the refinement of the XYZ-centroids of 9814 reflections above 20 $\sigma(I)$ with 5.726° < 2θ < 50.59°. Data were corrected for absorption effects using the Multi-Scan method (TWINABS) [S3]. The ratio of minimum to maximum apparent transmission was 0.861. The calculated minimum and maximum transmission coefficients (based on crystal size) are 0.938 and 0.990.

The structure was solved and refined using SHELXTL Software Package [S4,S5] $P\bar{1}$, with Z = 2 for the formula unit, $C_{102.01}H_{140.02}F_{6.91}N_{7.59}Na_{2.30}O_{39.28}$. The refinement was performed for merged data (multiplicity = 1) limited up to 2θ = 50.1° with R_{sig} = 0.0449 giving 20322 independent reflections. The final anisotropic full-matrix least-squares refinement on F^2 with 1807 variables converged at $R1$ = 6.99%, for the observed data and $wR2$ = 20.84% for all data. The goodness-of-fit was 1.051. The largest peak in the final difference electron density synthesis was 0.399 e$^-$/Å^3 and the largest hole was -0.396 e$^-$/Å^3 with an RMS deviation of 0.061 e$^-$/Å^3. On the basis of the final model, the calculated density was 1.312 g/cm^3

and $F(000)$, 2414 e^-. The all appropriate crystal data and refinement parameters are collected in **Table S2**.

The measured sample was oligocrystalline containing a few slightly rotated domains. Due to partial overlap of the diffraction spots integration of the reflections and scaling were based on two main domains followed by the merging data to HKLF4 format with the refined twin fractions yielding 0.7012:0.2988.

The structure is severely disordered and contains in its asymmetric part one tripodal ligand and non-stoichiometric amount of sodium trifluoroacetate with some solvent species (ethyl acetate, acetonitrile, methanol, water). Numbering scheme of atoms in the ligand is presented in **Figure S72.**

. Both crown ether moieties in the **site A** and **site B** are occupied in 100% by sodium ions. The **site C** contains Na^+ ions with overall occupancy of ca. 0.3. The cation is disordered over two positions with refined ratio yielding 0.237(2):0.065(2). In the **site A** sodium ion is additionally coordinated by O8B atom from neighboring ligand and by disordered, over three positions [occupancy ratio: 0.801(3):0.145(3):0.055(3)], 100% of trifluoroacetate anion. In the **site B** sodium ion is additionally coordinated by O8A atom from neighboring ligand and either by 0.698(2) of acetonitrile solvent or 0.302(2) of trifluoroacetate ion. In the close proximity of the anion site, ethyl acetate solvent is present, disordered over three possible positions with refined occupancy ratio yielding 0.410(3):0.382(3):0.108(3), with the overall occupancy of this site fixed at 0.9. Finally, in the **site C** not fully occupied sodium ion is additionally coordinated by O2OB atom. This O moiety belongs to carbonyl group of the trifluoroacetic anion occurring in the structure with ca. 30% nonstoichiometric amount - the same as coordinated Na^+ cation. Sodium moiety in in the **site C** is also coordinated by carbonyl atoms of disordered over two positions ethyl acetate solvent [occupancy ratio: 0.237(2):0.065(2)]. When the sodium cation is absent the crown ether ring is filled with methyl group of acetonitrile solvent – the one coordinating Na^+ ion in **site B**. In addition such empty ring in the **site C** also contains methyl groups of disordered over two positions ethyl acetate molecule [occupancy ratio: 0.605(2):0.093(2)], thus total occupancy of ethyl acetate located close to crown ether moiety in **site C** is equal 100%, however the solvent is disordered over four possible positions. In the close proximity of this solvent molecule there is partial occupancy water moiety present disordered over two positions with arbitrary assigned occupancies equal 0.25 and 0.30. The fractional occurrence of Na^+ ion in the **site C** results in disorder of aliphatic chain of the crown ether which is alternatively distributed over three locations with occupancy ratio yielding 0.553(3):0.062(3):0.384(3). The structure contains also fully ordered trifluoroacetate anion which links amide binding domains located in **site A** and **site B**. Moreover, in the structure there is one fully ordered ethyl acetate molecule and there are two sites with disordered solvent molecules. In one of the cases there is a combination of ethyl acetate disordered over two positions with acetonitrile all of

them with refined occupancy yielding 0.641(3):0.271(3)0:0.088(3). The last solvent site contains a combination of disordered molecules: ethyl acetate, acetonitrile ,methanol located in six positions close to main residues (ethyl acetate and acetonitrile) and water. Their occupancies, in the order of appearance are as follows: 0.798(5), 0.798(5), 0.202(5), 0.202(5), 0.1, 0.1, 0.1, 0.1, 0.1. The values with esd in parentheses stands for moieties with refined occupancies.

To preserve reasonable geometry of disordered moieties number of distance and angles restraints (SIMU instructions) was used together with restrains for atomic displacement parameters (SADI instructions). In the checkCIF report two Alert type B are present, corresponding to too short distance between H atoms. One of this alert results from slightly crowded ordered central part of the ligand, whereas the second denotes possible O-H…O hydrogen bond between methanol and ethyl acetate.

All major component, non-hydrogen atoms with occupancies larger than 50% were refined anisotropically. Most of hydrogen atoms were placed in calculated positions and refined within the riding model. Positions of six hydrogen atoms of amide fragments engaged in hydrogen bonds were refined. The temperature factors of all hydrogen atoms were not refined and were set to be equal to either 1.2 or 1.5 times larger than U_{eq} of the corresponding heavy atom. Hydrogen atoms of hydroxy group in methanol solvent molecules as well as all H atoms in water molecules were not assigned. The atomic scattering factors were taken from the International Tables [S6]. Molecular graphics was prepared using Mercury CSD 2020 program [S7]. Thermal ellipsoids parameters, with numbering corresponding to Figure 1X, are presented at 50% probability level in **Figure S73**. Packing diagrams of **1 + Na_triFlac** crystal structure are shown in **Figure S74**.

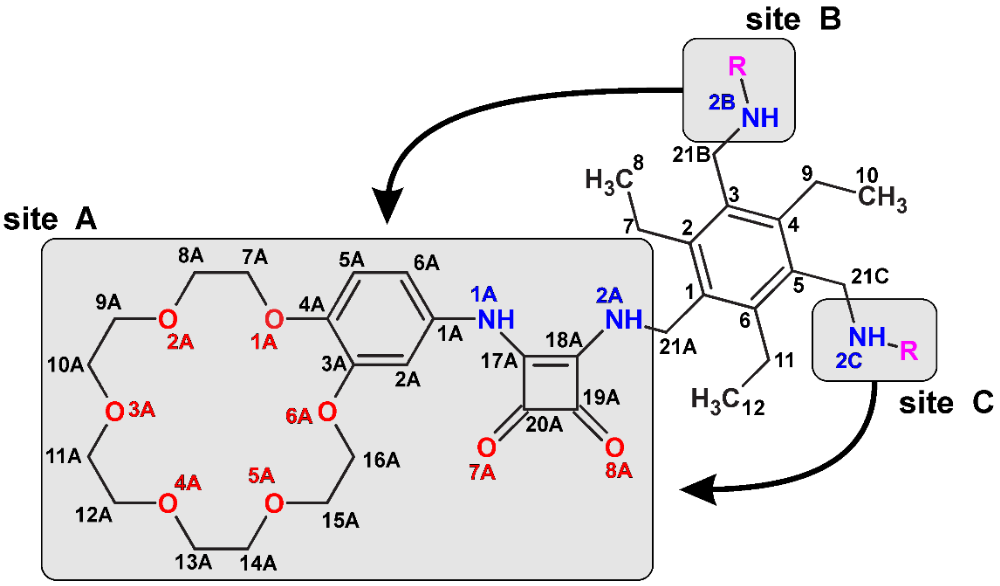

Fig. S72. Numbering scheme of atoms in the ligand used during crystal structure refinement.

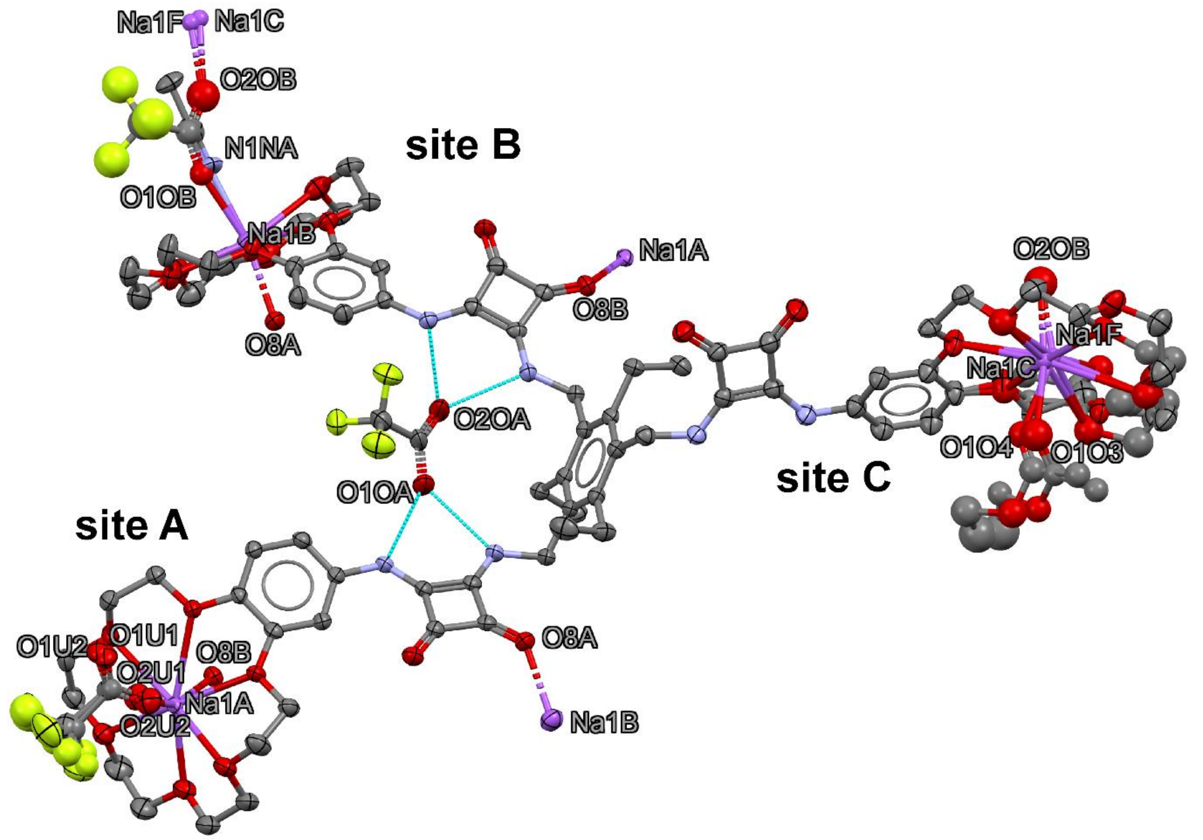

Fig. S73. Atomic displacement parameters at 50% probability level with numbering present for selected atoms for **1 + Na_triFlac**. Hydrogen atoms omitted for clarity.

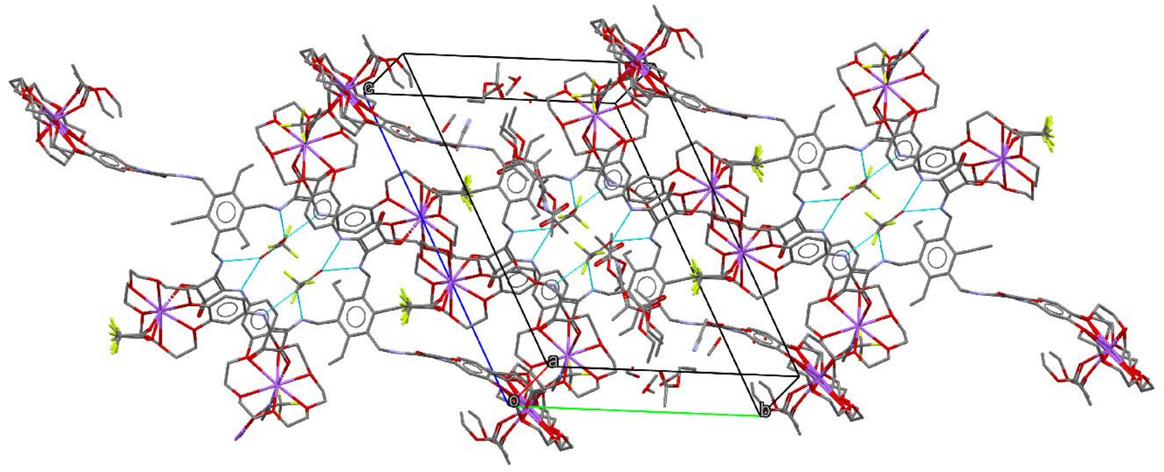

Fig. S74. Packing diagrams of **1 + Na_triFlac**. Hydrogen atoms omitted for clarity.

Table S2. Data collection and structure refinement for **1 + Na_triFlac**.

**Formula**	$C_{102.01}H_{140.02}F_{6.91}N_{7.59}Na_{2.30}O_{39.28}$, corresponding to approx.:<br>**1** +*2.30* × [**sodium trifluoroacetate**] +<br>*4.61* × [**ethyl acetate**] +<br>*1.58* × [**acetonitrile**] +<br>*0.80* × [**methanol**]* +<br>*0.65* × **H_2O***	
**M_x/ g·mol^{-1}**	2288.94	
***T*/ K**	130.0(5)	
**λ/ Å**	0.71073	
**Crystal size**	0.086 × 0.307 × 0.569	
**Space group**	$P\bar{1}$	
**Unit cell dimensions**	a = 13.9030(10) Å<br>b = 19.1752(13) Å<br>c = 24.2017(16) Å	α = 106.491(2)°<br>β = 98.390(2)°<br>γ = 105.570(2)°
**V/ Å^3, Z**	5784.7(7), 2	
**D_x/ g·cm^{-3}**	1.312	
**μ/ mm^{-1}**	0.114	
**F(000)**	2414	
**θ_{min}, θ_{max}**	2.86°, 25.05°	
**Index ranges (merged data)**	$-16 \leq h \leq 16$, $-23 \leq k \leq 22$, $0 \leq l \leq 29$	
**Reflections collected/ independent**	208133/ 21096 **<br>R_{int} = 0.0674**	
**Completeness**	99.1%	
**Absorption correction**	Multi-Scan	
**T_{max}, T_{min}**	0.990, 0.938	
**Structure solution technique**	direct methods	
**Refinement method**	Full-matrix LSQ on F^2	
**Data / restraints / parameters**	20322 / 603 / 1807	
**GOF on F^2**	1.051	

**Final *R* indices**	13630 data; $I > 2\sigma(I)$<br>$R1 = 0.0699$, $wR2 = 0.1894$<br>all data<br>$R1 = 0.1085$, $wR2 = 0.2084$
**Extinction coefficient**	0.0013(4)
$\Delta\rho_{max}$, $\Delta\rho_{min}$	0.397, -0.396 e·Å^{-3}

* Oxygen moieties with no hydrogen atoms assigned

** Values corresponding to $2\theta = 50.96°$

8. References

S1. *APEX3*, Bruker AXS Inc., Madison, WI, **2017**.

S2. *SAINT*, Bruker AXS Inc., Madison, WI, **2017**.

S3. *TWINABS*, Bruker AXS Inc., Madison, WI, **2012**.

S4. Sheldrick, G. M. SHELXT - Integrated Space-Group and Crystal-Structure Determination. *Acta Crystallogr., Sect. A*: *Found. Adv*. **2015**, *71*, 3–8.

S5. Sheldrick, G. M. Crystal Structure Refinement with SHELXL. *Acta Crystallogr., Sect. C: Struct. Chem*. **2015**, *71*, 3–8.

S6. Cowley, J. M. *International Tables for Crystallography*, ed. A. J. C. Wilson, Kluwer, Dordrecht, The Netherlands, **1992**, *vol. C*, pp. 223–245.

S7. Macrae, C. F.; Sovago, I.; Cottrell, S. J.; Galek, P. T. A.; McCabe, P.; Pidcock, E.; Platings, M.; Shields, G. P.; Stevens, J. S.; Towler, M.; P. A., Wood Mercury 4.0: from visualization to analysis, design and prediction, *J. Appl. Cryst.*, **2020**, *53*, 226-235.
